# Non-specific lipid transfer proteins in maize

**DOI:** 10.1186/s12870-014-0281-8

**Published:** 2014-10-28

**Authors:** Kaifa Wei, Xiaojun Zhong

**Affiliations:** School of Biological Sciences and Biotechnology, Minnan Normal University, Zhangzhou, 363000 China

## Abstract

**Background:**

In plant, non-specific lipid transfer proteins (nsLTPs) are small, basic proteins that have been reported to be involved in numerous biological processes such as transfer of phospholipids, reproductive development, pathogen defence and abiotic stress response. To date, only a tiny fraction of plant nsLTPs have been functionally identified, and even fewer have been identified in maize [*Zea mays* (Zm)].

**Results:**

In this study, we carried out a genome-wide analysis of nsLTP gene family in maize and identified 63 nsLTP genes, which can be divided into five types (1, 2, C, D and G). Similar intron/exon structural patterns were observed in the same type, strongly supporting their close evolutionary relationship. Gene duplication analysis indicated that both tandem and segmental duplication contribute to the diversification of this gene family. Additionally, the three-dimensional structures of representative nsLTPs were studied with homology modeling to understand their molecular functions. Gene ontology analysis was performed to obtain clues about biological function of the maize nsLTPs (ZmLTPs). The analyses of putative upstream regulatory elements showed both shared and distinct transcriptional regulation motifs of *ZmLTP*s, further indicating that ZmLTPs may play roles in diverse biological processes. The dynamic expression patterns of *ZmLTPs* family across the different developmental stages showed that several of them exhibit tissue-specific expression, indicative of their important roles in maize life cycle. Furthermore, we focused on the roles of maize nsLTPs in biotic and abiotic stress responses. Our analyses demonstrated that some *ZmLTPs* exhibited a delayed expression pattern after the infection of *Ustilago maydis* and differentially expressed under drought, salt and cold stresses, and these may be a great help for further studies to improve the stress resistance and tolerance in maize breeding.

**Conclusions:**

Our results provide new insights into the phylogenetic relationships and characteristic functions of maize nsLTPs and will be useful in studies aimed at revealing the global regulatory network in maize development and stress responses, thereby contributing to the maize molecular breeding with enhanced quality traits.

**Electronic supplementary material:**

The online version of this article (doi:10.1186/s12870-014-0281-8) contains supplementary material, which is available to authorized users.

## Background

As its name implies, plant lipid transfer protein (LTPs) were termed because of their functions that transfer phospholipids and fatty acids between membranes *in vitro* [1]. They were also named non-specific LTPs (nsLTPs) due to the character of non-specific binding to different lipids. Plant nsLTPs are small, basic proteins, usually about 6.5 ~ 10.5 kDa in size, characterized by an eight cysteine motif (8CM) backbone with the general form C-Xn-C-Xn-CC-CXC-Xn-C-Xn-C [2]. Almost all nsLTPs carry an N-terminal signal peptide in their nascent polypeptides. Thus, these proteins are likely secreted to the cell exterior for functioning. Many nsLTPs also possess a sequence for the post-translational addition of a glycosylphosphatidylinositol (GPI)-anchor, which attaches the protein to the exterior side of the plasma membrane [3]. The conserved features of plant nsLTPs include four defined disulfide bonds formed by eight Cys residues. Furthermore, the crystal structures of plant nLTPs were comprised of four or five alpha helices (α-helices), with a central hydrophobic cavity where the lipid binding takes place [4].

Based on the molecular weight (Mw) of the mature proteins, plant nsLTPs can be classified into two main types, nsLTP1 (9 kDa) and nsLTP2 (7 kDa) [5]. According to the sequence similarity, Boutrot et al. classified 49 out of the 52 rice *nsLTPs* and 45 out of the 49 Arabidopsis *nsLTPs* into nine types (I, II, III, IV, V, VI, VII, VIII, and Y) [2]. Recently, *nsLTPs* have been categorized into four major and several minor types by sequence similarity, intron position and spacing between the cysteine residues, as well as a potential glycophosphatidylinositol modification site within their encoded proteins [6]. *In vitro* studies showed that plant nsLTPs have the ability to facilitate the inter-membrane exchange and transfer of various amphiphilic molecules including phospholipids, glycolipids, steroids, acyl-CoAs and fatty acids. Several structures of plant nsLTPs have been resolved by X-ray and NMR spectroscopic techniques [4,7,8]. The typical fold of nsLTP1 is characterized by four to five α-helices connected by four disulfide bridges, partly wrapped by a long C-terminal segment. The overall structure delimits a large central hydrophobic cavity, where the alkyl moiety of lipids is inserted into. The backbone folds of nsLTP1 and nsLTP2 show structural similarities, however, drastic changes in their central hydrophobic cavity. Compared with these two earliest nsLTP types, the three-dimensional model of Arabidopsis DIR1 (AtDIR1) follows the general nsLTPs fold with five α-helices stabilized by four disulfide bonds around a central tunnel-shaped cavity. It was reported that most of the putative functions of nsLTPs are related to their ability to bind lipids in their hydrophobic cavity [9–11].

In the ensuing years, some members of nsLTPs have been functionally identified in plant species, including the involvement of cuticular waxes and cutin syntheses [12]. A recent study demonstrated that LTPg1, a Type G nsLTP from Arabidopsis, contribute either directly or indirectly to cuticular lipid accumulation [13]. In addition, nsLTPs are expressed in diverse organs and cells, including callus, germinating and maturing seeds, leaves, roots, stems, ovaries, anthers, and pollens [6,14]. The localization of nsLTP transcripts in anthers has been well reported in Arabidopsis and rice, and abundant Type III nsLTPs (also termed Type C nsLTPs) were expressed specifically in the anther tapetum, with levels peaking at the developmental stage of maximal pollen-wall exine synthesis [15]. A lipid transfer protein, OsC6, is widely distributed in anther tissues such as the tapetal cytoplasm, required for postmeiotic anther development in rice [16]. With respect to plant defense, nsLTPs are also recognized to be pathogenesis-related proteins and constitute the PR-14 family [17]. Some of them were demonstrated to share structural similarities with oomycetous elicitins and act as competitors with elicitins for specific receptors on membrane [18,19]. In addition, nsLTPs participate in long-distance signaling during pathogen defense [9]. Studies on purified nsLTPs confirmed their roles in abiotic stress tolerance [20]. Transcript levels of *nsLTPs* increased in response to drought, salt and cold stresses in many cases [21]. Stabilization of membranes, cuticle deposition and/or changes in cell wall organization have been claimed as their putative roles in the responses to these stress factors. In tobacco, nsLTP genes up-regulated during drought-induced cuticular wax deposition [22]. Overexpression of the pepper CALTP1 gene in Arabidopsis increased its tolerance to NaCl and drought stresses at various vegetative growth stages [23]. Arabidopsis *LTP3* act as a target of MYB96 to be involved in plant tolerance to freezing and drought stresses [24].

In previous studies, only a small portion of *nsLTPs* from maize have been well characterized. These include, *MZm3-3*, which is expressed specifically in the tapetum during male gametogenesis [25]; *Zm*-LTP (*ZmLTP1.2*; GRMZM2G010868), which binds to calmodulin (CaM) in a Ca^2+^-independent manner [26]; *ZmLTP3* (*ZmLTP1.1*; GRMZM2G126397), which is induced by mannitol, salt and SA treatments [27]; *BETL-9* (*ZmLTPd6*; GRMZM2G087413), which is transcribed in the outer surface of the developing endosperm [28]. As the genome of inbred line B73 has been sequenced completely, it is the time to initiate functional annotation and to perform more comprehensive analysises of maize nsLTP gene family. To date, genome-wide overview of the maize nsLTP gene family was yet to be reported. Therefore, as the first step to elucidate the functions of ZmLTPs, a genome-wide study for this gene family is necessary. In this study, we identified 63 genes encoding 77 putative nsLTPs in the maize genome that can be classified into five types. Detailed analyses including primary sequence, phylogenetic relationships, gene structure, chromosome location, gene duplication and divergence, three-dimensional structure, gene ontology, promoter and expression profilings were performed. Based on microarray, RNA-sequencing and real-time quantitative PCR (qRT-PCR), we analyzed the expression patterns of ZmLTP genes in different tissues and developmental stages, and took a further step towards understanding plant responses to biotic and abiotic stresses. Our analysis provides novel insights on ZmLTP gene family to support further functional research on nsLTP gene family in plants, particularly the proteins that may have important functions in response to biotic and abiotic stresses.

## Results and discussion

### Identification of nsLTP members in maize and sorghum

In order to identify the complete and non-redundant nsLTPs in maize, an accurate scan of the maize proteome was performed. Initially, 130 potential ZmLTPs were identified in the maize genome following the removal of those sequences with an incomplete 8CM domain. Then, each of the deduced protein sequences was manually assessed through the analysis of the cysteine residue patterns. Subsequently, 40 proline-rich or hybrid proline-rich proteins, which were characterized by a high proportion of proline, histidine and glycine residues in the sequence comprised between the signal peptide and the 8CM, were removed (Additional file 1: Table S1). Next, two α-amylase/trypsin inhibitors, three prolamin storage proteins and three 2S albumin storage proteins were also discarded. Additionally, five transcript forms lacking the GPI-anchor signal peptide (GRMZM2G174680_P02, GRMZM2G083725_P02, GRMZM2G006047_P01, GRMZM2G005991_P02, GRMZM2G116167_P02) were not taken into consideration. As a result, 77 proteins were confirmed as maize nsLTPs which were encoded by 63 nsLTP genes (Additional file 2: Table S2). Additionally, the same approach was used to identify nsLTPs in sorghum, which resulted in the identification of 58 nsLTP genes (Additional file 3: Table S3 and Additional file 4: Table S4).

### Sequence analysis and classification of putative maize nsLTPs

Previously, Edstam et al. have proposed that the plant nsLTPs can be divided into four major and several minor types according to sequence similarity, intron position and spacing between the cysteine residues [6]. In one of the major types, Type G, the transcripts encode a C-terminal signal sequence in addition to the N-terminal one, leading to a post-translational modification where a GPI-anchor is added to the protein. The classification of the identified type G ZmLTPs was based on the presence of a GPI modification site, as well.. In the second round of classification, the remaining sequences were grouped according to the identity matrix calculated from the multiple sequence alignments. When compared with the classification proposed by Edstam et al., we found that 73 out of the 77 ZmLTPs could be classified into five types (1, 2, C, D and G). Among the nsLTPs that were present in four flowering plants (maize, sorghum, rice and Arabidopsis), the variation in the number of nsLTPs across types and species was detected (Additional file 5: Table S5). In type G, 26, 24, 27, and 29 nsLTPs were found in maize, sorghum, rice and Arabidopsis, respectively. Whereas, only 2 nsLTP genes were distributed to Type E in Arabidopsis. Noticeably, a similar number of nsLTP genes were found in Type 1 and Type 2. It is thus conceivable that this difference may be due to gene duplication and loss. The main characteristic of plant nsLTPs is the presence of eight cysteine residues in a highly conserved region (C-Xn-C-Xn-CC-Xn-CXC-Xn-C-Xn-C). In order to establish a specific 8CM consensus for each obtained nsLTP type, we conducted a multiple sequence alignment using the 8CMs from the 77 ZmLTPs (Additional file 6: Figure S1). The amino acid sequence alignment of the 8CMs of ZmLTPs reveals a variable number of inter-cysteine amino acid residues (Table 1).

**Table 1:** **Some characteristics for the different types of non-specific lipid transfer proteins found in maize**

**Type**	**Number of members**	**GPI-anchored**	**Spacing pattern**										
1	8	No	C	X_9,10_	C	X_14,15,17_	CC	X_18,19_	CXC	X_21-24_	C	X_13_	C
2	9	No	C	X_7_	C	X_12,13_	CC	X_8,9_	CXC	X_23_	C	X_6_	C
C	2	No	C	X_9_	C	X_14,19_	CC	X_9_	CXC	X_12_	C	X_6_	C
D	16	No	C	X_9,10,13,14_	C	X_11,12,14,16-18_	CC	X_9,11,12_	CXC	X_11,21-24,26_	C	X_6-10_	C
G	26	Yes	C	X_5,6,9,10,12_	C	X_8,13-18,21_	CC	X_12-14,18_	CXC	X_21,22,24-27,29,41_	C	X_8-10,13,20_	C

Character “X” represents any amino acid, and the Arabic numeral following “X” stands for the numbers of amino acid esidues.

In this study, all the characteristics of the 77 ZmLTPs were summarized in Additional file 2: Table S2. 96% of the nsLTP precursors were initially synthesized with a signal peptide of 16-46 amino acids. The putative subcellular targeting of the 77 ZmLTP pre-protein sequences was analyzed. As expected, most of the proteins are predicted to be secreted except for ZmLTPg21, which have been predicted to be cytoplasm protein (Additional file 2: Table S2). At the pre-protein level, the ZmLTPd1 and ZmLTPd4 deduced proteins are identical, as are the ZmLTPd2 and ZmLTPd10 deduced proteins. After cleavage of their signal peptide, the ZmLTPg20.1 and ZmLTPg20.2 mature proteins are identical. Therefore, before potential post-translational modifications, the 63 ZmLTP genes encode 74 different mature proteins. 29 ZmLTPs were found to have one or more phosphorylation sites and most of the phosphorylated sites were located on serine residues at the C-terminal (Additional file 7: Table S6). To clearly understand the sequence characteristics of ZmLTPs, we further analyzed the pI (isoelectric point) values, Mw values, and CXC motifs of all available ZmLTPs. As shown in Additional file 2: Table S2, maize nsLTPs are small and their molecular masses usually range from 6,854 Da to 11,107 Da except for Type G. Type 1 and Type D nsLTPs were mostly 9 kDa proteins and Type 2 and Type C nsLTPs were 7 kDa proteins. The Mw value of Type G nsLTPs was much higher than that of other types due to the presence of supernumerary amino acid residues located at the C-terminal of the deduced mature proteins. Judging from the pI value, Type 1, 2, C and D nsLTPs are mostly alkaline proteins. The majority of Type G nsLTPs are weakly alkaline or acidic (Additional file 2: Table S2). The average molecular mass and the theoretical pI are 8,963 Da and 9.27 pI, respectively. As for the CXC motif, most residues at the X position in Type 1 nsLTPs are hydrophilic, while in Type 2, C, D and G, the X position is usually occupied by a hydrophobic residue (Additional file 6: Figure S1). These conserved hydrophobic or hydrophilic residues may play significant roles in the biological functions of ZmLTPs [29].

### Phylogenetic analysis of the maize, sorghum, rice and Arabidopsis nsLTPs

To analyze the phylogenetic relationship of the nsLTPs among maize, sorghum, rice and Arabidopsis, 274 nsLTPs from these four species were analyzed (Additional file 8: Figure S2). We performed a multiple sequence alignment of the 8CM domain sequences from maize, sorghum, rice and Arabidopsis and then generated a phylogenetic tree by the neighbor-joining method. Previously, Edstam et al. has divided the plant nsLTPs into ten types [6]. On the basis of the comparison between the previous dataset and ours, the six groups classified here were in agreement with the Type 1, 2, C, D, E and G of nsLTPs. As shown in Additional file 8: Figure S2, members in Type 1 and 2 formed specific clades, indicating that the genes in these major nsLTP types share a common ancestor. The sequences from the minor Type C and E also formed separated clades. Since relatively high bootstrap values were observed in the internal branches of Type 1 and Type C, it clearly showed the derivation of statistically reliable pairs of possible homologous proteins sharing similar origin from a common ancestor. It was also worth noting that Type E nsLTPs seem to be monophyletic, and this may mean that the monocotyledon plants discarded these genes during the evolutionary divergence between monocots and dicots.

### Intron-exon structure of the maize nsLTP gene family

As a kind of evolutionary relic, the intron-exon arrangement carries the imprint of the evolution of a gene family. Investigation of ZmLTP gene structures revealed low diverse distribution of intronic regions amid the exonic sequences, and 31 ZmLTP genes (36 Alternative splicing forms) were predicted to be interrupted by 1-2 introns positioned 4 to 67 bp downstream of the codon encoding the eighth cysteine in the 8CM (Additional file 9: Figure S3). Alternative splicing forms of the same gene usually had similar intron–exon structures, indicating that the distinct proteins from a single transcript may share similar functions. Additionally, it is interesting to find a similar exon/intron pattern in each group. For instance, the nsLTP genes in Type 1, 2 and C lacked an intron, while all members in Type G contained 1-3 introns. Except for *ZmLTPd6*, *ZmLTPd13*, *ZmLTPd1*4 and *ZmLTPd15*, no intron was detected in the coding regions of Type D genes. Our comparative analysis with the gene structures of *AtLTPs* and *OsLTPs* indicated that the exon/intron structures of *ZmLTPs* are similar to those of Arabidopsis and rice (Additional file 10: Figure S4).

### Chromosomal localization and gene duplication of ZmLTP genes

**Figure 1:**
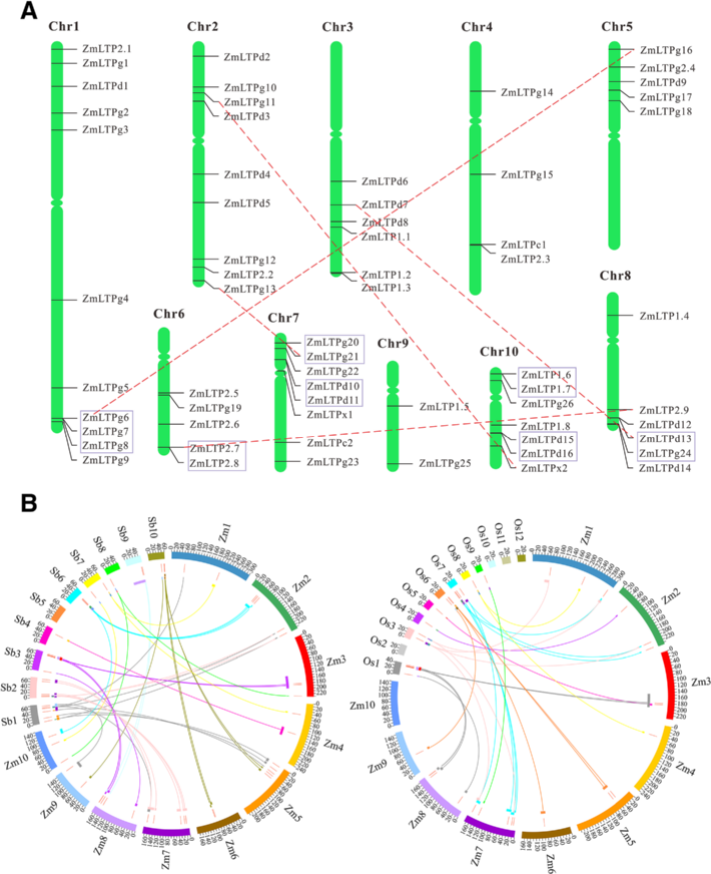
**Genome distribution and synteny analysis of nsLTP genes from maize and its relatives (rice and sorghum). (A)** Distribution of 63 ZmLTP genes on ten maize chromosomes. Chromosomal distances are given in Mb. Chromosome numbers are indicated at the top of each bar. The gene names on the right side of each chromosome correspond to the approximate locations of each nsLTP gene. The tandem duplicated gene clusters are marked in boxes, and segmental duplication genes are connected by dashed lines in red. **(B)** Synteny analysis of nsLTP genes from maize and its relatives (rice and sorghum). Positions of putative nsLTP genes are shown using red line. Boxes represent the syntenic blocks. Colors are assigned to the syntenic regions according to the colors of the corresponding chromosomes. Innermost colored lines show synteny between nsLTP genes.

*In silico* mapping of the gene loci showed that 63 *ZmLTPs* were unevenly assigned to maize ten chromosomes (Figure 1A). Chromosome 1 contained the maximum number of *ZmLTPs* (11), while the minimum number (2) were presented on Chromosome 9. However, several chromosomes lacked *ZmLTPs* in some regions, such as the long arm of chromosome 5 as well as the short arms of Chromosome 3, 6 and 9. The exact position (in bp) of each *nsLTP* on maize chromosome is given in Additional file 2: Table S2.

Gene duplication is generally believed to be a major driving force in evolutionary innovation, giving rise to genomic complexity. Maize originated from an ancient allotetraploid and has undergone several rounds of whole-genome duplication events during its gene evolution [30,31]. Segmental and tandem duplications are known to be the main causes leading to gene family expansions. In this study, a total of seven tandem repeats (*ZmLTPg6/ZmLTPg7/ZmLTPg8*; *ZmLTP2.7/ZmLTP2.8*; *ZmLTPg20/ZmLTPg21*; *ZmLTPd10/ZmLTPd11*; *ZmLTPd12/ZmLTPd13*; *ZmLTP1.6/ZmLTP1.7* and *ZmLTPd15/ZmLTPd16*) were identified in the maize genome. One significant cluster of three Type G genes was found on Chromosome 1. What’s more, six direct repeat tandems were identified on chromosome 6, 7, 8 and 10. Genes in the same cluster were closely related to one another. For instance, ZmLTPd15 and ZmLTPd16 protein sequences shared 95% similarity and the two genes were physically located next to each other in the same chromosomal region. In addition, five sister pairs (*ZmLTPg6*/*ZmLTPg16*, *ZmLTPg11*/*ZmLTPx2*, *ZmLTPg13*/*ZmLTPg21*, *ZmLTPd7*/*ZmLTPd13* and *ZmLTP2.7*/*ZmLTP2.9*) appeared to be generated from segmental duplication events due to their positions on the same duplicated gene blocks (Figure 1A and Additional file 11: Table S7). Furthermore, we analyzed the evolution of nsLTP genes among maize, sorghum and rice (Figure 1B and Additional file 11: Table S7). 22 out of 63 maize nsLTP genes had collinear genes in rice, while 35 had syntenic members in sorghum. According to this analysis, two paired nsLTP genes (*ZmLTPg21*/*OsLTPg17* and ZmLTPg13/OsLTPg17) were located in genomic regions with synteny between the maize and rice genomes. Seven paired nsLTP genes (*ZmLTPg16*/*SbLTPx1*, *ZmLTPg11*/*SbLTPg18*, *ZmLTPx2*/*SbLTPg18*, *ZmLTP2.9*/*SbLTP2.5*, *ZmLTPg21*/*SbLTPg10*, *ZmLTPg13*/*SbLTPg10* and *ZmLTPg13*/*SbLTPg4*) were located in genomic regions with synteny between the maize and sorghum genomes, indicating that these genes may be derived from a common ancestor. Interestingly, one of the sister pairs (*ZmLTPg6*/*ZmLTPg16*) had a unique, syntenic *nsLTPs* in sorghum, which indicated that they might be generated from segmental duplication after the divergence of maize and sorghum.

### Divergence rate of the maize nsLTP genes

The ratio (Ka/Ks) of nonsynonymous substitution rate (Ka) versus synonymous substitution rate (Ks) is widely used as an indicator of selective pressure [32]. As shown in Table 2, the Ka/Ks ratios of 5 duplicated gene pairs were less than 1, indicating that they seemed to be under purifying selection; however, the Ka/Ks ratios of 9 duplicated gene pairs were more than 1, suggesting that they subjected to positive selection. Positive selection was thought to be one of the major forces for the emergency of new motifs/functions in protein after gene duplication, and the divergence of the duplicated genes was driven by positive selection [33]. Based on the substitution rates analysis, we found that 64.29% of the duplicated gene pairs had strong positive selection pressure, implying that positive selection contributed to further gene diversification in the *ZmLTP* family. In addition, the divergent times between the duplicated gene pairs were analyzed (Table 2). The duplication events for the 5 segmental duplications were estimated to have occurred approximately between 9.43 and 34.06 million years ago (Mya), while the duplication events for the 9 tandem duplications occurred approximately between 9.13 and 50.46 Mya. Fossil data along with the phylogenetic studies estimated that the different grass families diverged from a common ancestor 50 to 70 Mya [34]. The divergence times on the nodes of the tree were estimated with the non-parametric rate smoothing method of Sanderson, assuming that maize and rice diverged 50 Mya [35]. Duplication events for one tandem duplicated gene pair (*ZmLTPg20*/*ZmLTPg21*) occurred around 50.46 Mya, after origin of grasses and before divergence of rice and maize (within last 70 to 50 million years). The ancestor of maize and sorghum diverged about 12 Mya and subsequently a whole genome triplication (WGT) event occurred in the maize approximately 5 Mya [34]. Duplication events for 10 gene pairs (*ZmLTPg11*/*ZmLTPx2*, *ZmLTPg13*/*ZmLTPg21*, *ZmLTPd10*/*ZmLTPd11*, *ZmLTP2.7*/*ZmLTP2.9*, *ZmLTPd7*/*ZmLTPd13*, *ZmLTP1.6*/*ZmLTP1.7*, *ZmLTP2.7*/*ZmLTP2.8*, *ZmLTPd12*/*ZmLTPd13*, *ZmLTPg6*/*ZmLTPg7* and *ZmLTPg6*/*ZmLTPg8*) occurred within last 39.91 to 18.12 million years, after divergence of rice and maize, but before maize and sorghum were separated from each other. Duplication events for the other two gene pairs (*ZmLTPg6*/*ZmLTPg16* and *ZmLTPd15*/*ZmLTPd16*) occurred around last 9 million years, after maize and sorghum were separated, and before a whole genome duplication (WGD) event occurred in the maize. Therefore, evolutionary origin of the maize nsLTP genes might undertake three evolutionary stages.

**Table 2:** **Ka/Ks analysis and estimate of the absolute dates for the duplication events between the duplicated ZmLTP genes**

**Duplicated pair**	**Ka**	**Ks**	**Ka/Ks**	**Date (Mya)**	**Duplicate type**	**Purifying selection**	**Group**
*ZmLTPg6*/*ZmLTPg16*	0.091	0.123	0.7423	9.43	Segmental	Yes	Type G
*ZmLTPg11*/*ZmLTPx2*	0.127	0.236	0.5382	18.12	Segmental	Yes	Type G
*ZmLTPg13*/*ZmLTPg21*	0.334	0.379	0.8816	29.18	Segmental	Yes	Type G
*ZmLTPd10*/*ZmLTPd11*	0.359	0.519	0.6920	39.91	Tandem	Yes	Type D
*ZmLTPd15*/*ZmLTPd16*	0.022	0.119	0.1828	9.13	Tandem	Yes	Type D
*ZmLTP2.7*/*ZmLTP2.9*	0.475	0.443	1.0720	34.06	Segmental	No	Type 2
*ZmLTPd7*/*ZmLTPd13*	0.306	0.237	1.2910	18.24	Segmental	No	Type D
*ZmLTP1.6*/*ZmLTP1.7*	0.401	0.368	1.0911	28.28	Tandem	No	Type 1
*ZmLTP2.7*/*ZmLTP2.8*	0.339	0.314	1.0806	24.15	Tandem	No	Type 2
*ZmLTPd12*/*ZmLTPd13*	0.688	0.401	1.7169	30.81	Tandem	No	Type D
*ZmLTPg6*/*ZmLTPg7*	0.681	0.483	1.4094	37.17	Tandem	No	Type G
*ZmLTPg6*/*ZmLTPg8*	0.624	0.469	1.3300	36.06	Tandem	No	Type G
*ZmLTPg7*/*ZmLTPg8*	0.607	0.484	1.2533	37.24	Tandem	No	Type G
*ZmLTPg20*/*ZmLTPg21*	0.738	0.656	1.1245	50.46	Tandem	No	Type G

### The features of the three-dimensional structures of major nsLTPs

In order to better understand the non-specific binding between the hydrophobic ligands and ZmLTPs, ZmLTP1.2, ZmLTP2.1 and ZmLTPd5 were selected as representative sequences of Type 1, 2, and D for structural modeling (Additional file 2: Table S2). The crystal structures of maize ZmLTP1.6 (PDB ID: 1FK7) [7], wheat nsLTP2 (PDB ID: 1TUK) [8], AtDIR1 (PDB ID: 2RKN) [4] were selected as templates for structural modeling based on searches against the PDB using the Basic Local Alignment Search Tool (BLAST) at NCBI (http://blast.ncbi.nlm.nih.gov/) with the target Type 1, 2 and D protein sequences as baits.

**Figure 2:**
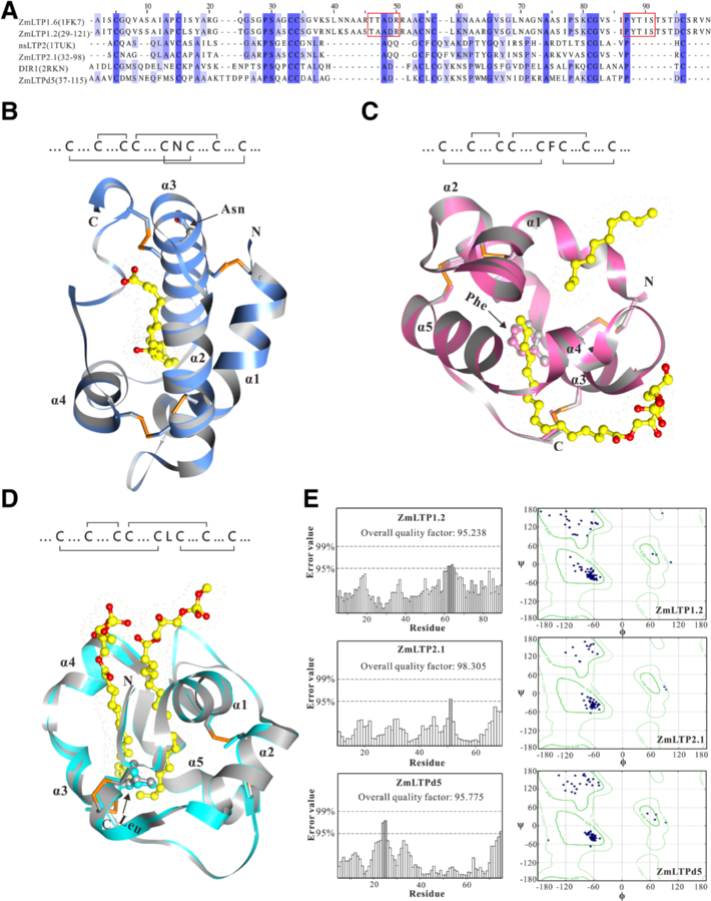
**The structure features of maize nsLTPs. (A)** Sequence alignment of maize ZmLTP1.6 (1FK7; amino acids (aa) 1-93), wheat nsLTP2 (1TUK; aa 1-67), AtDIR1 (2RKN; aa 1-77), ZmLTP1.2 (aa 29-121), ZmLTP2.1 (aa 32-98) and ZmLTPd5 (aa 37-115). Identical residues are highlighted by blue background. Consensus residues Thr-X-X-Asp-Arg and Pro-Tyr-X-Ile-Ser are marked in boxes. **(B)** Schematic representation of the cystein pairing pattern and superposition of the backbone trace of ZmLTP1.2 (blue) and ZmLTP1.6 (grey) complexed with ricinoleic acid (shown as ball-and-stick; carbons in yellow and oxygen in red). The four disulfide bonds are shown in orange. **(C)** Schematic representation of the cystein pairing pattern and superposition of the backbone trace of ZmLTP2.1 (pink) and nsLTP2 (grey) complexed with LPG (L-α-palmitoyl glycerol) lipid ligands (shown as ball-and-stick; carbons in yellow and oxygen in red). The four disulfide bonds are shown in orange. **(D)** Schematic representation of the cystein pairing pattern and superposition of the backbone trace of ZmLTPd5 (cyan) and the crystal structure of AtDIR1 (grey) in complex with two lysophosphatidyl choline molecules (shown as ball-and-stick; carbons in yellow and oxygen in red.). The four disulfide bonds are shown in orange. **(E)** ERRAT result depicting overall quality factor and Ramachandran plot of ZmLTP1.2, ZmLTP2.1 and ZmLTPd5.

Based on the ZmLTP1.6, nsLTP2 and AtDIR1 structures, the amino acids 29-121 of ZmLTP1.2, 32-98 of ZmLTP2.1, and 37-115 of ZmLTPd5 could be modeled with the sequence identities of 88.17%, 62.69%, and 51.95%, respectively. Our structural analysis showed that ZmLTP1.2 and ZmLTP1.6 have typical features of plant nsLTPs, including two conserved pentapeptides, T-T/A-A-D-R (positions 46-50) and P-Y-T-I-S (positions 87-91). It has been reported that these two consensus pentapeptides (T/S-X-X-D-R/K and P-Y-X-I-S) were important in catalysis or binding [5]. ZmLTPd5 and AtDIR1 contain high proline content (positions 25-32) that is not highlighted in nsLTP2 and ZmLTP2.1. It is noteworthy that proline-rich regions are involved in protein-protein interactions [36]. As aforementioned, X is a hydrophilic residue in the CXC motif of Type 1 nsLTPs. However, a hydrophobic residue was found at the X position in Type 2 and Type D nsLTPs. As illustrated in Figure 2A, in the CXC motif, asparagines between the two cysteines in ZmLTP1.2 and ZmLTP1.6 are replaced by a hydrophobic amino acid, phenylalanine, in ZmLTP2.1 and nsLTP2, whereas in ZmLTPd5 and AtDIR1, the X position are occupied by a hydrophobic residue, leucine. The hydrophobic residues in the CXC motif of ZmLTP2.1, nsLTP2, ZmLTPd5 and AtDIR1 are buried inside the molecule, whereas the hydrophilic residue of ZmLTP1.2 and ZmLTP1.6 are at the surface (Figure 2B, C and D). In the template protein ZmLTP1.6, the Cys residues 1-6, 2-3, 4-7, and 5-8 are paired, whereas the Cys residues 1-5, 2-3, 4-7, and 6-8 are paired in the template protein AtDIR1, similarly to the Type 2 nsLTPs. These observations suggested that the central residue of the CXC motif may govern the cysteine pairing and influence the overall fold of the protein. The crystal structure homology models were validated in terms of stereochemical quality by Ramachandran plot and the ERRAT prediction (Figure 2E).

From the superposition structure, we find that ZmLTP1.2 and ZmLTP1.6 share a common structural fold stabilized by four disulfide bonds, and the four prominent α-helices are packed against a flexible C-terminal arm formed by a series of turns, which forms a cap over the hydrophobic cavity (Figure 2B). The three-dimensional structure of ZmLTP2.1 showed that two adjacent hydrophobic cavities seem able to expand the hydrophobic cavity to accommodate the alkyl ligand (Figure 2C). The structure of ZmLTPd5 is in accordance with AtDIR in the aspects of the order and orientation. Their α-helix topology is well conserved and the four cysteine bridges superimpose well, showing high similarity in the three-dimensional structure (Figure 2D). Interestingly, the Arabidopsis mutant defective (*dir1-1*) was compromised in the production or transmission of an essential mobile signal to promote long-distance signaling [9]. Whether other Type D nsLTPs have the functions related to systemic resistance signalling remain to be further investigated.

### Gene ontology analysis of the maize nsLTPs

**Figure 3:**
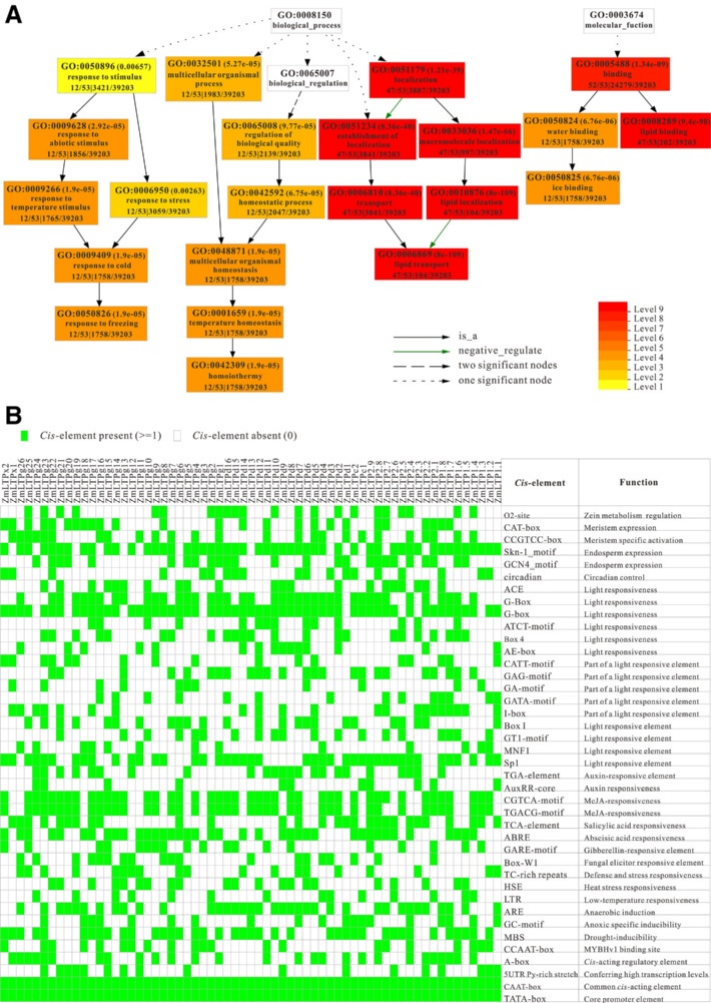
**Gene ontology and promoter analysis of** ***ZmLTPs*** **. (A)** Enriched gene ontologies in maize nsLTPs. Singular enrichment analysis was performed in AgriGO to identify enriched gene ontologies associated with high tillering lines. Each box shows the GO term number, the p-value in parenthesis, and GO term. First pair of numerals represents number of genes in input list associated with that GO term and number of genes in input list. Second pair of numerals represents number of genes associated with a particular GO term in the maize database and total number of maize genes with GO annotations in the maize database. Box colors indicate levels of statistical significance: yellow = 0.05; orange = e-05; and red = e-09. **(B)** The *cis*-elements that have been identified in more than ten ZmLTP genes. The associated *cis*-elements and their known biological functions based on the annotation in PlantCARE are shown for each ZmLTP gene.

In order to comprehend the unique aspects of maize nsLTPs, Gene Ontology (GO) enrichment analysis of the functional significance was performed. Out of 77 ZmLTP proteins, annotation could not be performed for 24 proteins. Besides, 53 ZmLTPs were defined in 22 significant GO terms (Additional file 12: Table S8). The analysis showed that 53 ZmLTPs were separated into two main categories (biological process and molecular function), which included 18 and 4 significant GO terms, respectively (Figure 3A). For the enriched biological processes, the common categories are “response to stimulus” followed by “response to abiotic stimulus” and “response to stress”, “multicellular organismal process”, “biological regulation” and “establishment of localization”. Noteworthy, about 47 ZmLTPs were shown to participate in “lipid transport” (GO: 0006869), which is concordance with the molecular role of nsLTP in transporting hydrophobic molecules *in vitro*, suggesting that ZmLTPs play an important role in carrying membrane components to the growing site of elongating cells. The enriched GO terms also include the parent term “response to abiotic stimulus” with enriched children term “response to cold”, and 22.64% (12 of 53) ZmLTPs were exhibited to participate in “response to stress stimulus” (particularly in cold). This highlights the putative association of nsLTP proteins in stress tolerance behavior of maize. In case of molecular functions, about 88.68% (47 of 53) ZmLTPs were shown to participate in “lipid binding” (GO: 0008289). One of the interesting observations was that some significantly enriched terms are involved in “water binding” and “ice binding”. To summarize, the GO analysis indicated that ZmLTPs may be involved in diverse biological processes.

### Key *cis*-elements within the promoter regions of ZmLTP genes

*In-silico* analysis of 1 kb upstream region (from translation start site) of 63 maize nsLTP genes revealed the presence of various regulatory elements, which are associated with development, abiotic or biotic stress signaling, and hormone signaling (Additional file 13: Table S9). To avoid the biases in analysis, only *cis*-elements identified in at least ten different genes were considered. As shown in Figure 3B, except for TATA-box and CAAT-box, G-box is the most frequently found type of *cis*-elements, and the regulatory element involving light responsiveness seems to be enriched in *ZmLTPs*. Nearly 80% of *ZmLTPs* contained Skn-1_motifs which might account for endosperm expression. Stress-responsive *cis*-regulatory elements identified in this study included fungal elicitor responsive element (Box-W1), defense and stress responsive element (TC-rich repeats), heat stress responsive element (HSE), low temperature responsive element (LTR), anaerobic-response element (ARE and GC-motif), MYB binding site (MBS) involving in drought-inducibility and phytohormone-responsive elements, like auxin responsive element (TGA-element and AuxRR-core), methyl jasmonate responsive element (CGTCA-motif and TGACG-motif), salicylic acid responsive element (TCA-element), abscisic acid responsive element (ABRE) and gibberellic acid-responsive element (GARE). With a few exceptions, *ZmLTPs* contained circadian elements, which may be responsible for its distinct diurnal expression pattern. Other regulatory elements, such as O2-site (involved in zein metabolism regulation), DRE (involved in responses to dehydration, low temperatures and salt), CAT-box (related to meristem expression) and CCGTCC-box (related to meristem specific activation) were also presented. Especially, the promoters of 39 *ZmLTPs* contained MBS elements ranging from 1 to 5 copies (Additional file 13: Table S9), indicating the important role of MYB transcription factors in regulating *ZmLTPs*. Some recent reports have shown that nsLTP can act as targets of MYB to regulate plant tolerance to freezing and drought stresses [24,37], suggested that the ZmLTP members participate in some abiotic stress signaling.

### The diverse roles of nsLTPs in maize development

**Figure 4:**
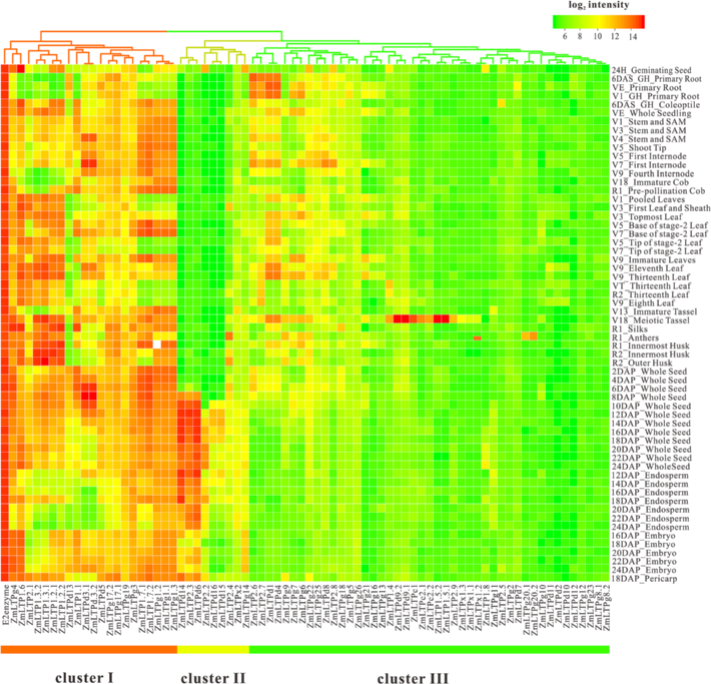
**Hierarchial clustering display of 75 ZmLTP transcripts detected on the NimbleGen maize genome array in 60 distinct tissues representing 11 major organ systems of inbred line B73.** The average log signal values were used for clustering. The color scale (representing log signal values) is shown at the upper right.

As for multigene family, the analysis of gene expression patterns often provides useful clues to decide genes function. To investigate the temporal and spatial expression patterns of the nsLTP genes in the maize life cycle, hierarchical clustering was performed to visualize a global transcription profile of the ZmLTP genes across the 11 organs during diverse developmental stages. As depicted in Figure 4, the heatmap can be apparently divided into three clusters. Cluster I contains 21 members (excluding the E2 enzyme as the reference). Cluster II has 9 members, and 45 members belong to Cluster III. Genes in Cluster I obviously have relatively high expression levels, with the mean log-signal values of each gene ranging from 9.0 to 14.9 (Additional file 14: Table S10). Conversely, Cluster III contains those genes with relatively low expression levels and the average log-signal value range is between 5.2 and 9.2. In Cluster II, genes are expressed at a moderate level with the mean log-signal values ranging from 6.1 to 8.5. To elucidate putative differentially expressed genes in specific organs or stages, the coefficient of variation (CV value; CV = S/X_mean_, where S represents the standard deviation and X_mean_ describes the mean expression level of a gene across all the tissues) of each gene in the three clusters was calculated (Additional file 14: Table S10). A house keeping gene in maize, which encodes a ubiquitin-conjugating enzyme (E2), was used as internal reference in our expression analysis [38]. The results showed a huge variation among all the genes with the CV values ranging from 1.01% to 52.97%. Cluster I has the least expression variability from 1.01% to 26.80%, indicating a most stable expression pattern relative to other ZmLTP genes. *ZmLTP1.1*, the most changeable genes in cluster I, whose homolog in rice, *Photoperiod-sensitive dwarf 1* (*Psd1*, *Os01g60740*), was previous demonstrated to encode a lipid transfer protein that may participate in regulation of plant cell division and elongation [39]. One gene (*ZmLTP1.2*), which can bind to calmodulin in a Ca^2+^-independent manner [26], displayed high expression levels at nearly all of the maize organs and/or stages of development analyzed except for root, immature cob and endosperm. A total of 12 nsLTP protein-encoding genes were found to be highly expressed in anthers. 7 *ZmLTPs* were found to be highly expressed in leaf at different developmental stages, whereas, 5 *ZmLTPs* specially accumulated at V5_Base of stage-2 Leaf, V7_Base of stage-2 Leaf, V9_Immature Leaves, V9_Eleventh Leaf and V9_Thirteenth Leaf. Cluster II showed the highest expression variability, ranging from 11.95% to 52.97%. There are 8 genes with CV values more than 15% in Cluster II, among which 3 genes (*ZmLTPd14*, *ZmLTP2.3* and *ZmLTPd6*) have CV values more than 45% and display transcript accumulation at late seed developmental stages (10 to 24 DAP) and endosperm. It has been reported that *BETL9* (*ZmLTPd6*) has its expression restricted to the endosperm transfer cells, which share their position as entrance gates to the developing seed for nutrients coming from the maternal tissues [28]. There is an increasing evidence that this protein might be involved in the defense of the developing seed against mother plant-borne pathogens [40,41]. Genes in Cluster III showed an apparent fluctuation, with the CV values ranging from 4.82% to 31.83%. In this context, Cluster III can be further divided into two subclusters, with genes in the first subcluster having higher signal intensity values than those in the second subcluster. In the first subcluster, four *ZmLTPs* (*ZmLTP2.6*, *ZmLTP2.7*, *ZmLTPd1* and *ZmLTPd4*) displayed high expression levels during root development and this finding suggested that ZmLTPs may participate in maize root development. The organ-specific expression dynamics revealed the distinct expression patterns of ZmLTP genes throughout the entire life cycle in maize. For example, two ZmLTP genes, *ZmLTPg20* and *ZmLTP2.5*, were specifically expressed in the anthers and coleoptile, respectively. There are 6 genes (*ZmLTP1.4*, *ZmLTP1.5*, *ZmLTP2.9*, *ZmLTPc1*, *ZmLTPc2* and *ZmLTPd9*), with CV values among 15.54% to 23.92% in the second subcluster, distinctly increasing their expression levels in meiotic tassel. Among them, *ZmLTPc2* is highly homologous to *MZm3-3*, which is expressed specifically in the tapetum during male gametogenesis of maize [25]. Furthermore, four nsLTP genes (*Os09g35700*, *Os01g49650*, *Os01g12020* and *Os08g43290*) were reported to be expressed in rice anther, which are putatively related to exine synthesis in sporophytic anthers of progressive developmental stages [42]. Interestingly, these four rice *nsLTPs* were the homologs of *ZmLTPc2*, *ZmLTP2.9*, *ZmLTP1.4* and *ZmLTPc1*, respectively. Synthesis of lipidic components in anthers, including the pollen exine, is essential for plant male reproductive development. Therefore, this finding further suggested that nsLTPs play significance roles in male reproductive development.

### Pathogen responses of *ZmLTPs* to the fungal infection

Plant nsLTPs are capable of inhibiting bacterial and fungal pathogens and are, therefore, thought to play an important role in plant defense [18]. To discover the ZmLTP genes involved in maize pathogen response, microarray data collected after the treatments of 12, 24 hours, 2, 4, 4.5, 8 days of *Ustilago maydis* (*U. maydis*) infection were analyzed. *U. maydis* is a ubiquitous pathogen of maize which depends on living tissue for proliferation and development. A total of 41 probe sets found on the maize 18 k GeneChip could be assigned to 36 different ZmLTP genes (Additional file 15: Table S11). The log2 ratio values (Treated/Control) were illustrated by a heatmap, showing the fold change in expression of each *ZmLTP*. As shown in Additional file 16: Figure S5, most of the *ZmLTPs* exhibited a delayed expression pattern after the infection of *U. Maydis*. 23 *ZmLTPs* distinctly increased their expression levels and accumulated mostly on the 4 and 8 days post infection, especially of *ZmLTPg1*, *ZmLTP1.1* and *ZmLTP1.7*, suggesting that these genes might participate in the pathogen response. Interestingly, some ZmLTP genes show up-regulated expression levels over time, especially of *ZmLTP2.8*, demonstrating a similar response pattern to this kind of fungus infection. Besides, the expression levels of *ZmLTPd2* and *ZmLTP2.1* show a rapid increase after infection, suggesting their high sensitivity to *U. maydis* infection. One nsLTP from Arabidopsis (AtDIR1) has been suggested to be involved in long-distance signaling during pathogen defense [9]. Therefore, it is reasonable to find a transcript accumulation of the *ZmLTPd5*, a AtDIR1 homologue from maize, after the infection of *U. maydis*. In addition to the genes up-regulated after *U. maydis* infection, there are also some ZmLTP genes show a decline of expression levels as time goes by, such as *ZmLTPg23* and *ZmLTPd6*. These genes might function in other biological processes or respond to other kinds of pathogen attacks. Taken together, these results presented here indicated that many genes in this family might participate in the pathogen response.

### Transcriptional responses of maize nsLTP genes against abiotic stresses

**Figure 5:**
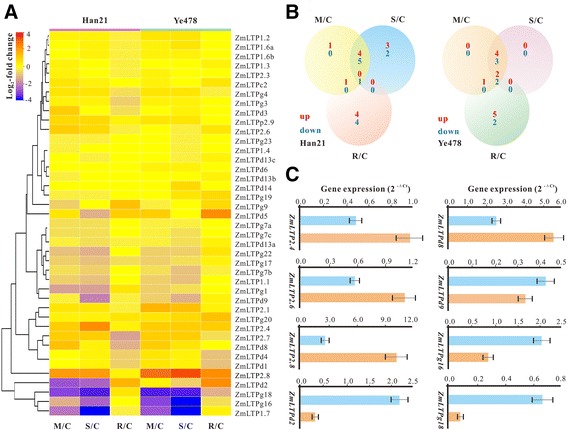
**Differential gene expression of** ***ZmLTPs*** **under drought stress. (A)** Expression profiles of 36 *ZmLTPs* under moderate drought stress (M/C), severe drought stress (S/C) and re-watering (R/C) as compared to control seedlings in Han21 and Ye478, respectively. Log2-based fold changes were used to create the heatmap. **(B)** Venn diagrams for the number of differentially expressed ZmLTP genes under moderate drought stress (M/C), severe drought stress (S/C) and re-watering (R/C) as compared to control seedlings in Han 21 and Ye478, respectively. A gene is considered differentially expressed if it is up- or down-regulated at least two-fold with a P value <0.05 under any of the given conditions. **(C)** Quantitative RT-PCR analysis for selected ZmLTP genes in drought treatments. The maize UBQ1 gene was used as endogenous control to normalize data. The values represented are the mean of two biological replicates, each with three technical replicates. Error bars represent standard deviations determined with two biological replicates.

Apart from their inducibilities upon pathogen infection, nsLTP genes are also responsive to abiotic stresses like drought, cold and salt [21,43]. To investigate the possible role of *ZmLTPs* under drought stress, the microarrary data of two inbred lines, drought-tolerant line Han21 and drought-sensitive line Ye478, under different drought treatments were used [44]. Following whole-chip data processing, 57.14% (36 of 63) ZmLTP genes, about 41 probes (some genes have two or more probe sets) in the microarray, were extracted for analysis (Additional file 15: Table S11 and Figure 5A). Using fold change >2 and *P* value <0.05, ZmLTP genes with changeable expression profiles in at least one conditions were regarded as differentially expressed genes (Figure 5B). The result showed that 14 and 13 *ZmLTPs* were differentially regulated by drought and/or re-watering treatment(s) in Han21 and Ye478 maize inbred lines, respectively. Interestingly, some *ZmLTPs* appeared to be up-regulated in Han21 under drought stress, suggesting that they may have a role in drought stress response in maize. For example, the transcript levels of *ZmLTP2.4*, *ZmLTP2.7* and *ZmLTP2.8*, were significantly up-regulated under moderate and severe drought stresses (Figure 5A). Furthermore, *ZmLTP2.6* and *ZmLTPd8* showed high-abundance transcript levels under moderate drought condition compared to untreated samples. To verify the expression patterns of these predicted genes under drought stress, 8 differentially expressed genes were selected for qRT-PCR analysis and the primers used in this assay are listed in Additional file 17: Table S12. As shown in Figure 5C, *ZmLTP2.4*, *ZmLTP2.6*, *ZmLTP 2.8* and *ZmLTPd8* were obviously up-regulated in water deficient, whereas *ZmLTPd2*, *ZmLTPd9*, *ZmLTPg16* and *ZmLTPg18* were down-regulated under drought stress. Taken together, qRT-PCR-based expression profiling for these selected genes confirmed the outcome of microarray analysis.

In addition to DNA microarray analysis, RNA-seq analysis was performed to investigate the functions of *ZmLTPs* involved in the differential drought sensitivity of maize reproductive (ovary) and vegetative tissues (leaf) under both drought and well-watered conditions [45]. Nearly 66.67% (42) of *ZmLTPs* were reliably detected by RNA-seq data (Additional file 18: Table S13). Out of 42 expressed genes, 3 genes (*ZmLTP2.8*, *ZmLTPg14* and *ZmLTP2.2*) were distinctly upregulated and 17 genes were down-regulated in the drought-stressed ovary tissue, while 10 (*ZmLTPd3*, *ZmLTP2.8*, *ZmLTPd4*, *ZmLTPd1*, *ZmLTP2.6*, *ZmLTPg2*, *ZmLTP1.1*, *ZmLTPg3*, *ZmLTPg1* and *ZmLTPd5*) were significantly up-regulated and 2 (*ZmLTP2.9* and *ZmLTPg24*) were down-regulated in the drought-stressed leaf meristem, suggesting that these ZmLTP genes may exhibit tissue-specific expression under drought stress.

Many nsLTPs in plants have been reported to be closely related to drought stress. Plants have a cuticle on the surface of their leaves that provides a protective barrier against the environmental adversities, such as drought stress. In Arabidopsis, AtLTPg4, which have close phylogenetic relationship with ZmLTPg14 in maize, play a role in wax and/or cutin monomer transport [46]. In our study, the expression level of *ZmLTPg14* was increased in drought-stressed ovary tissue, implying a possible role in drought resistance. *ZmLTP1.2*, which was reported as the target of calmodulin, was significantly down-regulated in drought-stressed ovary tissue but up-regulated in drought-stressed leaf meristem. As expected, a stresses-response gene, *ZmLTP1.1*, also increases its expression level in the drought-stressed leaf meristem, whereas it was down-regulated in the drought-stressed ovary tissue. In any case, the analysis of the expression profiles based on both microarray and RNA-seq methods proved that ZmLTPs may play important roles in maize tolerance to drought stress. While further work is required for understanding how ZmLTPs are mechanistically linked to drought stress responses in maize.

Apart from understanding the effect of drought stress on the transcript levels of various maize nsLTP genes, it is also important to conceive about the expression level of these genes under the salt stress. Root is the first organ directly exposed to salt stress. To better understand the roles of ZmLTP genes in salt stress responses, RNA-seq analysis was performed to explore the expression profiles of *ZmLTPs* in three root types of maize [primary root (PR), seminal root (SR) and crown root (CR)] under salt stress. Nearly 60.32% (38 of 63) of *ZmLTPs* were detected by RNA-seq. As shown in Additional file 19: Figure S6, 3 ZmLTP genes (*ZmLTP2.4*, ZmLTP*d3* and *ZmLTPg17*) and 4 genes (*ZmLTP2.8*, *ZmLTPd3*, *ZmLTPd11* and *ZmLTPg17*) were found to be at least two-fold up-regulated in the PR and SR under salt stress, respectively. Under salt stress, 11 ZmLTP genes (*ZmLTP1.1*, *ZmLTP1.5*, *ZmLTP2.4*, *ZmLTP2.8*, *ZmLTPd3*, *ZmLTPd9*, *ZmLTPd11*, *ZmLTPg2*, *ZmLTPg4*, *ZmLTPg5* and *ZmLTPg10*) significantly increased their expression levels in CR. It can be concluded that the gene expression profiles of *ZmLTPs* are more variable in the CR than those in PR and SR under salt stress. One ZmLTP gene, *ZmLTPd3*, was highly induced by salt stress in PR, SR and CR, suggesting that it may play an important role in salt stress response in maize. In addition, *ZmLTP1.1* was specifically expressed in CR, which is accordance with previous report that *ZmLTP1.1* may participate in salt resistance [27].

It is well-known that plants are constantly exposed to a variety of environmental stresses. Among them, the low temperature constitutes a key factor that can influence plant growth, development and crop productivity. The accumulation of soluble sugar and proline are important to protect plants against cold stress [47]. In previous research, the expression of rice cold-inducible nsLTP gene in transgenic *Phalaenopsis amabilis* improved its adaptive responses to cold stress. The examination of transgenic plants showed the increased accumulation of several compatible solutes such as total soluble sugars and proline [48]. To further dissect the biological function of ZmLTPs in cold tolerance, we investigate the possible roles of ZmLTPs under cold stress condition in two inbred maize lines, chilling-sensitive ETH-DL3 and chilling-tolerant ETH-DH7 [49]. Following whole-chip data processing, 54% (34 of 63) *ZmLTPs* have corresponding probe sets in the microarray, and a total of 36 probe sets on the maize 58 k NSF Array (some genes have two probe sets) were extracted for analysis (Additional file 20: Table S14). Three *ZmLTPs* (*ZmLTPd12*, *ZmLTPg9* and *ZmLTP1.5*) were defined as differentially expressed genes in response to cold stress (Additional file 21: Figure S7). Surprisingly, all of the three differentially expressed genes were up-regulated under severe cold stress. Among them, *ZmLTPd12* and *ZmLTPg9* were differentially expressed under the severe cold stress conditions in ETH-DH7, and *ZmLTP1.5* and *ZmLTPd12* was up-regulated under severe cold stress conditions in ETH-DL3.

Our results revealed effects of drought, salt and cold stress on transcript abundance of several members of maize nsLTP gene family, indicative of their important roles in abiotic stresses. Moreover, the promoters for most of *ZmLTPs* were found to contain one or more important *cis*-regulatory elements such as ABRE, MBS, TC-rich repeat and C-repeat/DRE (Additional file 13: Table S9), which might be involved in regulation of expression of *ZmLTPs* under these stress conditions. Thus, expression profiling against abiotic stresses provided novel insights into specific and/or overlapping expression patterns of nsLTP genes. These analyses will contribute to the studies for unraveling the molecular mechanisms of signal transduction, stress response, and developmental processes.

## Conclusions

We have identified 63 nsLTP genes in maize genome, and the members in this family can be divided into five types (1, 2, C, D and G). Comprehensive study of ZmLTPs provided some important features of this gene family such as gene structure, duplicated event, and three-dimensional structure. Additionally, gene ontology analysis was performed to obtain clues about biological function of the ZmLTPs. The analyses of putative upstream regulatory elements further indicated that ZmLTPs may be involved in diverse biological processes and provided new insights into the shared and different transcriptional regulation machineries of *ZmLTPs*. The expression profiles of *ZmLTPs* across the different developmental stages showed that some members of this family exhibit tissue-specific expression, indicative of their important roles in performing diverse developmental and physiological functions during the maize life cycle. Further, we focused on the roles of maize *nsLTPs* in stress responses. The result indicated that some *ZmLTPs* exhibited a delayed expression pattern after the infection of *Ustilago maydis* and differentially expressed under drought, salt and cold stress, which may be used in further studies for improving stress tolerance in maize. In a word, our results are significant contributions to maize molecular breeding with enhanced quality traits.

## Methods

### Retrieval and identification of nsLTPs in maize and sorghum genomes

Our search strategy used successive iterations of hidden Markov model (HMM) and BLAST searches to identify small, secreted nsLTPs in maize. The complete genome sequence of maize was downloaded from the maize sequence database (release version 5b; http://www.maizesequence.org/index.html). Starting with protein sequences annotated with the Pfam domain PF00234 (plant lipid transfer/seed storage/trypsin-alpha amylase inhibitor), we scanned maize proteome sequences using the hmmsearch from the HMMER 3.0 package [50] with default parameters. To increase the probability of detecting putative nsLTPs in maize, BLASTP searches were further performed against maize proteome sequences using all known nsLTP amino acid sequences from Arabidopsis and rice as baits. The results were merged, and redundant sequences were deleted. Then, the deduced protein sequences of candidate nsLTPs were manually examined to harbor the 8CM and proteins lacking the essential cysteine residues were removed. Additionally, the proteins encoding for the putative proline-rich proteins were excluded from further analysis. The remaining candidate proteins were submitted to the InterProScan (http://www.ebi.ac.uk/InterProScan) to verify the presence of nsLTP domains. The protein sequences of the 2S-albumins (At2S1 to At2S4) [51], and alpha amylase inhibitor (RATI) [52] were BLAST-searched against the rest of candidate nsLTP proteins to exclude possible cereal storage proteins. In addition, sorghum genome sequences were downloaded from the Joint Genome Institute plant genomics database (release version 1.4; http://www.Phytozome.net) and the same approach was used to identify nsLTPs in sorghum.

### Primary sequence analysis

All identified nsLTPs were analyzed for the presence of potential signal peptide cleavage sites using SignalP v4.0 program [53]. The subcellular localization of nsLTP was predicted by LocTREE 3 (https://rostlab.org/services/loctree3/). Following signal peptide removal, molecular weight and isoelectric point were computed for mature proteins with ExPasy’s pI/Mw tool (http://web.expasy.org/compute_pi/). The eight Cys residues of the 8CM domain were excluded during pI calculation, since Cys residues in a disulfide bond have no charge. Three prediction tools, PredGPI [54], big-PI Plant Predictor [55] and GPI-SOM [56] were used to determine the presence of GPI-anchor sites. Phosphorylation sites of the nsLTPs were predicted by P3DB (http://www.p3db.org/). A sequence identity matrix of the mature nsLTP sequences was computed using BioEdit v7.0.9.0 (http://www.mbio.ncsu.edu/bioedit/bioedit.html), which enable us to determine the gene subfamily assignment. The nomenclature was based on the guidelines proposed by Edstam et al. [6].

### Sequence alignment and phylogenetic reconstruction

The nsLTP amino acid sequences of Arabidopsis (*A. thaliana*) and rice (*O. sativa*) were downloaded from the TAIR (http://www.Arabidopsis.org/) and TIGR (http://rice.plantbiology.msu.edu/) databases, respectively. Multiple alignments of the 8CM part of the mature proteins were carried out using the ClustalW implemented in MEGA 5 software. Manually refined alignments were used as input in ProtTest v2.4 [57], which was set to all candidate models, a BIONJ tree, and the slow optimization strategy. The best model (WAG + I + G) was selected by the Akaike information criterion implemented in ProtTest. Then, the amino acid sequences of 8CM of maize, rice and Arabidopsis, totally 274 sequences were loaded into PhyML v3.0 software [58]. The confidence level of each node was estimated by the bootstrap procedure using 100 resampling repetitions of the data [59].

### Gene structure and conserved intron splicing site analysis

To explore the diverse exon-intron organization of maize nsLTP genes, we compared the predicted coding sequence (CDS) of maize nsLTP genes with their corresponding genomic sequences using GSDS software (http://gsds.cbi.pku.edu.cn). For a better visualization and comparison, the 5' UTR sequences were removed beforehand. The positions of the 8CM were retrieved with PAL2NAL (http://www.bork.embl.de/) by converting a multiple sequence alignment of 8CM sequences and the corresponding gene sequences into a codon alignment, and then marked in red [60].

### Genome distribution and gene duplication analysis

The genes were plotted separately onto the ten maize chromosomes according to their ascending order of physical position (bp), from the short arm telomere to the long arm telomere. As a gene family may be expanded through tandem and segmental duplication events, we intended to identify the mechanisms involved in the expansion of ZmLTP gene family. ZmLTP genes separated by no more than 10 intervening genes were considered as tandem duplication [61]. The putative othologs from rice, sorghum and Arabidopsis were assigned to corresponding maize nsLTP proteins according to searches from the TIGR database (http://rice.plantbiology.msu.edu/annotation_pseudo_apk.shtml). Syntenic information from all examined genes was collected from the Plant Genome Duplication Database (PGDD, http://chibba.agtec.uga.edu/duplication). The ideograms were created using Circos software [62].

### Estimation of synonymous and non-synonymous substitution rates

Synonymous and nonsynonymous substitution rates of duplicated genes were calculated by DnaSP v5.0 [63]. The the ratio of nonsynonymous to synonymous nucleotide substitution rates between paralogs was analyzed to detect the mode of selection. Ka/Ks ratio >1, <1 and =1 represent positive selection, purifying selection and neutral selection, respectively. The dates of the duplication events were calculated by the equation T = Ks/2λ × 10^-6^ Mya, (λ =6.5 × 10^− 9^) [64,65].

### Homology modeling of maize nsLTPs

All the maize nsLTP proteins were searched against the Protein Data Bank (PDB) by BLASTP with the default parameters to identify the best template. The data was fed in SWISS-MODEL [66] for predicting the protein structure by homology modeling. To visualize and edit the PDB models, interactive molecular graphics program Chimera v1.8 was used [67]. Ramachandran plots for the evaluation of the model were calculated using Chimera, and the amino acid environment was evaluated using ERRAT at the UCLA-DOE Structure Analysis and Verification Server (http://nihserver.mbi.ucla.edu/ERRAT/). A sequence alignment of various nsLTPs was made using the ClustalW implemented in MEGA 5 software, and the alignment was manually inspected and adjusted via Jalview (http://www.jalview.org/).

### Gene ontology analysis

The GO enrichment analysis was performed using the web-based tool AgriGO (http://bioinfo.cau.edu.cn/agriGO/index.php). The singular enrichment analysis (SEA) method was used to identify enriched GO terms, and the significantly enriched functional clusters were selected with an adjusted q-value less than 0.05. The program provides the output defining three categories of GO classification, namely, biological processes, cellular components and molecular functions.

### Promoter analysis of maize nsLTP genes

To investigate *cis*-elements in promoter sequences of maize nsLTP genes, the upstream sequences (~1000 bp) of each identified *ZmLTPs* were retrieved from B73 maize genome sequence using a Perl script. The PlantCARE website (http://bioinformatics.psb.ugent.be/webtools/plantcare/html/) was used to identify *cis*-elements in the promoters [68]. To avoid biases in analysis, only *cis*-elements identified in at least ten different genes were considered further.

### Microarray-based gene expression analysis

To gain an insight into the family-wide expression profile, microarray-based data analyses of both development and stress responses were carried out. In order to achieve the expression profilings of ZmLTP genes among different organs and development stages, transcriptome data of the genome-wide gene expression atlas of maize inbred line B73 were obtained from publicly available database PLEXdb with the accession number ZM37. Gene expression data of drought stress and pathogen infection were downloaded from GEO database with accession numbers GSE16567 and GSE10023, respectively. The microarray data of cold stress were downloaded from ArrayExpress database with accession number E-MTAB-1315. To identify probe sets for ZmLTP genes, probe match against the maize GeneChip assemblies were performed on the Affymetrix Netaffx website (http://www.affymetrix.com/estore/) and PLEXdb. The data were normalized using a robust multi-chip average (RMA) algorithm [69]. Limma package was applied to data processing, and heatmaps representing log2-transformed probe intensities were generated with the gplots package [70]. A hierarchical clustering algorithm was applied to determine similar patterns in expression profiles. Using fold change >2 and *P* value <0.05, ZmLTP genes having changed expression profiles in at least one of the three conditions examined were regarded as differentially expressed genes under drought stress, whereas the response must be at least 2.8-fold in cold stress.

### Differential gene expression profiles based on RNA-seq

Previous maize drought transcriptome performed by RNA-seq analysis was used to investigate the expression patterns of drought-responsive genes in drought-treated and well-watered fertilized reproductive (ovaries) and vegetative tissue (basal leaf meristem) [45]. The ovaries and the basal leaf meristem each have four libraries corresponding to well-watered and drought-stressed tissue, with one biological replicate respectively. The transcript abundance of each gene was estimated by fragments per kilobase of exon per million fragments mapped (FPKM). To achieve the expression patterns of ZmLTP genes in three root types (PR, SR and CR) under salt stress, the RNA-seq data were downloaded from the ArrayExpress database with accession number E-GEOD-53995. The transcript abundance of each gene was estimated by reads per kilobase per million mapped reads (RPKM).

### Real-time PCR analysis

Maize seeds were incubated at 30°C for germination and sown in plastic pots containing sandy loam soil of 0.1 m^3^ each. Pots of each plant were divided into 3 groups with 2 replicates. Plant growth was carried out in a controlled condition of 14 h photoperiod, 28 ± 2°C day/night temperature and 55% air humidity. At 34 days from the date of sowing (at this time, all plants had 3–4 fully developed leaves), one set of pots was kept as control (well-watered) and two other sets were used for drought by withholding the water for 6 days. At the end of this period, the volumetric soil water content (measured at the 5 cm depth from the top of soil level) was approximately 12.5% for the drought-stressed plants, compared with approximately 30% for the control plants. The leaves of the seedlings were harvested, immediately frozen in liquid nitrogen and stored at -80°C. Then, total RNA was extracted from leaves after subjecting to drought stress using Trizol reagent, followed by DNase I treatment to remove any genomic DNA contamination. About 2 μg of total RNA was reverse-transcribed using Takara RNA PCR Kit to generate cDNA. QRT-PCR was performed on iCycler iQ5 Multicolor real-time PCR detection system (Bio-Rad) by using Power SYBR Green PCR Master Mix (Applied Biosystems). Two biological replicates of each sample and three technical replicates of each biological replicate were used. The thermal cycling conditions were as follows: 50°C for 2 min, 95°C for 10 min, 40 cycles of 95°C for 15 s and 60°C for 1 min. Quantification of gene expression was performed by 2^-ΔCt^ method. Maizeubiquilin-1 (UBQ1) gene was used as internal control for normalization.

### Availability of supporting data

All the supporting data are included as additional files.

## Additional files

Additional file 1: Table S1. Maize genes encoding proteins with a Pfam domain PF00234 which belong to hybrid proline-rich proteins, alpha-amylase/trypsin inhibitors, prolamin storage proteins and 2S albumin storage proteins. Additional file 2: Table S2. The occurrence of nsLTPs in maize, and some of their features. Additional file 3: Table S3. Designations used for nsLTP sequences in this report, and their corresponding database accession numbers. Additional file 4: Table S4. Sorghum genes encoding proteins with a Pfam domain PF00234 which belong to hybrid proline-rich proteins, alpha-amylase/trypsin inhibitors, prolamin storage proteins and 2S albumin storage proteins. Additional file 5: Table S5. A summary of nsLTP genes in maize, sorghum, rice and Arabidopsis. Additional file 6: Figure S1. Multiple sequence alignment of ZmLTPs. Amino acid sequences of the 8CMs were aligned using ClustalW implemented in MEGA5 software suite program and manually refined. The conserved cysteine residues are blue boxed. Additional file 7: Table S6. Predicted phosphorylation sites in ZmLTPs. Additional file 8: Figure S2. Unrooted phylogenetic tree between maize, sorghum, rice and Arabidopsis nsLTP gene families. Different species are denoted with different colors (maize in black, sorghum in green, rice in blue and Arabidopsis in red). Additional file 9: Figure S3. The map of intron-exon arrangement of maize nsLTP genes. Introns and exons are drawn to scale with the full encoding regions of their respective. Exons are depicted as green boxes, introns as connecting thin lines and 8CM as red boxes. Non-translated regions, when supported by full-length cDNA sequences, are shown in blue boxes. Additional file 10: Figure S4. The map of intron-exon arrangement of Arabidopsis and rice nsLTP genes. Introns and exons are drawn to scale with the full encoding regions of their respective. Exons are depicted as green boxes, introns as connecting thin lines and 8CM as red boxes. Non-translated regions, when supported by full-length cDNA sequences, are shown in blue boxes. Additional file 11: Table S7. Syntenic sites harboring nsLTP genes in maize, sorghum and rice. Additional file 12: Table S8. Genes list corresponding to the GO enrichment analysis by the AgriGO program. Additional file 13: Table S9. Analysis of *cis*-elements in the 1 kb sequence upstream of the translation initiation codon in ZmLTP genes. Additional file 14: Table S10. Average log2 signal intensity values and CV values of 75 detected ZmLTP transcripts in sixty distinct tissues representing eleven major organ systems of inbred line B73 ordered by three clusters. Additional file 15: Table S11. The probe sets of ZmLTP genes on the maize 18 k GeneChip in this study. Additional file 16: Figure S5. Normalized signal intensities of 41 probe sets representing 36 ZmLTP genes displayed for the microarray experiment, which was treated with *Ustilago maydis*. Log2 ratio values (shown by a green-red gradient) represents the fold change of the gene expression in the fungal infections. Additional file 17: Table S12. Primers used for real time PCR analysis. Additional file 18: Table S13. List of FPKM values of 42 ZmLTP genes in maize reproductive (ovary) and vegetative tissue (leaf) under both drought and well-watered conditions. Additional file 19: Figure S6. Expression profiles of 38 ZmLTP genes in three root types [primary root (PR), seminal roots (SR) and crown roots (CR)] under salt stress. Additional file 20: Table S14. The probe sets of ZmLTP genes on the maize 58 k NSF array in this study. Additional file 21: Figure S7. Microarray-based expression profiles of 36 probe sets representing 34 ZmLTP genes under cold stress condition in two inbred maize lines, chilling-sensitive ETH-DL3 and chilling-tolerant ETH-DH7.
