# Supplementary material for: Non-specific lipid transfer proteins in maize
Source: BMC Plant Biol. 2014 Oct 28;14:281. doi: 10.1186/s12870-014-0281-8 (PMC4226865; doi:10.1186/s12870-014-0281-8)
Supplement: Additional file 1: Table S1. — Maize genes encoding proteins with a Pfam domain PF00234 which belong to hybrid proline-rich proteins, alpha-amylase/trypsin inhibitors, prolamin storage proteins and 2S albumin storage proteins. [file 12870_2014_281_MOESM1_ESM.pdf]

Table S1. Maize genes encoding proteins with a Pfam domain PF00234 which belong to hybrid proline-rich proteins, alpha-amylase/trypsin inhibitors, prolamin storage proteins and 2S albumin storage proteins.

**hybrid proline-rich proteins**			
GRMZM2G379898	GRMZM2G351505	GRMZM2G037255	GRMZM2G094639
GRMZM2G059964	GRMZM2G372102	GRMZM2G372074	GRMZM2G345700
GRMZM2G162276	AC155352.2_FG010	GRMZM5G873271	GRMZM2G406313
GRMZM2G410338	GRMZM2G398807	GRMZM2G104945	GRMZM2G058208
GRMZM2G136367	GRMZM2G304378	GRMZM2G040689	AC234161.1_FG001
AC234161.1_FG002	GRMZM2G407189	GRMZM2G429000	GRMZM2G391272
GRMZM2G391286	GRMZM2G091534	GRMZM2G031354	GRMZM2G106324
GRMZM2G027221	GRMZM2G027167	GRMZM2G477697	GRMZM2G477685
**alpha-amylase/trypsin inhibitors**			
GRMZM2G304548	GRMZM2G404688		
**prolamin storage proteins**			
GRMZM2G060429	GRMZM2G138689	GRMZM2G138727	
**2S albumin storage proteins**			
GRMZM2G012148	GRMZM5G822180	GRMZM2G410134	
