# Supplementary material for: Non-specific lipid transfer proteins in maize
Source: BMC Plant Biol. 2014 Oct 28;14:281. doi: 10.1186/s12870-014-0281-8 (PMC4226865; doi:10.1186/s12870-014-0281-8)
Supplement: Additional file 2: Table S2. — The occurrence of nsLTPs in maize, and some of their features. [file 12870_2014_281_MOESM2_ESM.pdf]

Table S2. The occurrence of non-specific lipid transfer proteins in maize, and some of their features.

[1]ECM, eight-cysteine motif.

[2]AA, number of amino acids.

[3]Mw, molecular weight in Dalton.

[4]pI, isoelectric point (cysteine residues were not taken into account in the pI caculation).

[5]Subcellular target for the protein. S = secretory pathway, C = cytoplasm.

Name	Gene ID	Protein ID	Chr.	W/C	Locus	No. of introns in ORF	ECM[1]	Signal peptide	Mature protein			Subcellular localization[5]	Sorghum Ortholog	Rice Ortholog	Arabidopsis Ortholog
								AA[2]	AA	Mw[3]	pI[4]				
**Type 1**															
ZmLTP1.1	GRMZM2G126397	GRMZM2G126397_P01	3	W	184434264..184435351	0	CX{9}CX{14}CCX{19}CXCX{24}CX{13}C	28	95	9856.47	10.62	S	SbLTP1.2	OsLTP1.2, OsLTP1.4	AtLTP1.6, AtLTP1.10
ZmLTP1.2.1	GRMZM2G010868	GRMZM2G010868_P01	3	C	230606444..230608235	0	CX{10}CX{14}CCX{19}CXCX{22}CX{13}C	28	93	9087.23	11.4	S	SbLTP1.5, SbLTP1.6, SbLTP1.7	OsLTP1.21, OsLTP1.22, OsLTP1.14, OsLTP1.17, OsLTP1.18	AtLTP1.1, AtLTP1.4, AtLTP1.5, AtLTP1.8
ZmLTP1.2.2		GRMZM2G010868_P02	3	C	230607424..230608235	0	CX{10}CX{14}CCX{19}CXCX{22}CX{13}C	28	91	8873.99	11.4	S	SbLTP1.5, SbLTP1.6, SbLTP1.7	OsLTP1.21, OsLTP1.22, OsLTP1.14, OsLTP1.17, OsLTP1.18	AtLTP1.1, AtLTP1.4, AtLTP1.5, AtLTP1.8
ZmLTP1.3.1	GRMZM2G107839	GRMZM2G107839_P01	3	W	231823510..231824603	0	CX{9}CX{14}CCX{19}CXCX{22}CX{13}C	30	127	12983.2	10.11	S	SbLTP1.4	OsLTP1.12, OsLTP1.19, OsLTP1.20	AtLTP1.7, AtLTP1.11, AtLTP1.12
ZmLTP1.3.2		GRMZM2G107839_P02	3	W	231823868..231825026	0	CX{9}CX{14}CCX{19}CXCX{22}CX{13}C	30	93	9228.69	11.1	S	SbLTP1.4	OsLTP1.12, OsLTP1.19, OsLTP1.20	AtLTP1.7, AtLTP1.11, AtLTP1.12
ZmLTP1.4	GRMZM2G096234	GRMZM2G096234_P01	8	C	25946151..25947246	0	CX{9}CX{15}CCX{19}CXCX{23}CX{13}C	26	100	9890.46	4.53	S	SbLTP1.1	OsLTP1.1	-
ZmLTP1.5.1	GRMZM2G025026	GRMZM2G025026_P01	9	W	61863515..61865024	0	CX{9}CX{17}CCX{19}CXCX{21}CX{13}C	24	95	10163.43	4.52	S	SbLTP1.9	-	-
ZmLTP1.5.2		GRMZM2G025026_P02	9	W	61863624..61864642	0	CX{9}CX{17}CCX{19}CXCX{21}CX{13}C	24	93	9921.16	4.61	S	SbLTP1.9	-	-
ZmLTP1.6	GRMZM2G101958	GRMZM2G101958_P01	10	C	4165442..4166593	0	CX{9}CX{14}CCX{18}CXCX{22}CX{13}C	28	93	9026.15	11	S	SbLTP1.5, SbLTP1.6, SbLTP1.7	OsLTP1.21, OsLTP1.22, OsLTP1.14, OsLTP1.17, OsLTP1.18	AtLTP1.1, AtLTP1.4, AtLTP1.5, AtLTP1.8
ZmLTP1.7.1	GRMZM5G898755	GRMZM5G898755_P02	10	C	4243325..4244555	0	CX{9}CX{14}CCX{19}CXCX{23}CX{13}C	35	105	10164.64	9.86	S	SbLTP1.8	-	-
ZmLTP1.7.2		GRMZM5G898755_P01	10	C	4243574..4244555	0	CX{9}CX{14}CCX{19}CXCX{23}CX{13}C	35	94	9131.41	10.42	S	SbLTP1.8	-	-
ZmLTP1.8	GRMZM2G439268	GRMZM2G439268_P01	10	C	84608056..84608542	0	CX{9}CX{14}CCX{19}CXCX{22}CX{13}C	30	93	9799.24	5.21	S	SbLTP1.3	OsLTP1.7	-
**Type 2**															
ZmLTP2.1	GRMZM2G137329	GRMZM2G137329_P01	1	C	3000161..3000884	0	CX{7}CX{13}CCX{8}CXCX{23}CX{6}C	25	73	7399.45	11.05	S	SbLTP2.1, SbLTP2.3	OsLTP2.3, OsLTP2.7, OsLTP2.8, OsLTP2.9, OsLTP2.10, OsLTP2.11, OsLTP2.12	AtLTP2.5, AtLTP2.11, AtLTP2.12
ZmLTP2.2	GRMZM2G081464	GRMZM2G081464_P01	2	C	220468734..220469483	0	CX{7}CX{13}CCX{8}CXCX{23}CX{6}C	28	71	7474.71	11.38	S	SbLTP2.7	OsLTP2.6, OsLTP2.13	-
ZmLTP2.3	GRMZM2G406552	GRMZM2G406552_P01	4	C	195759880..195760460	0	CX{7}CX{13}CCX{8}CXCX{23}CX{6}C	19	78	7988.29	9.7	S	-		-
ZmLTP2.4	GRMZM2G387360	GRMZM2G387360_P01	5	W	23838144..23838818	0	CX{7}CX{13}CCX{8}CXCX{23}CX{6}C	28	68	7042.98	10.26	S	SbLTP2.1, SbLTP2.3	OsLTP2.3, OsLTP2.7, OsLTP2.8, OsLTP2.9, OsLTP2.10, OsLTP2.11, OsLTP2.12	AtLTP2.5, AtLTP2.11, AtLTP2.12
ZmLTP2.5	GRMZM2G039383	GRMZM2G039383_P01	6	C	86281901..86282589	0	CX{7}CX{12}CCX{8}CXCX{23}CX{6}C	20	74	7654.79	10.9	S	SbLTP2.7	OsLTP2.6, OsLTP2.13	-
ZmLTP2.6	GRMZM2G403007	GRMZM2G403007_P01	6	C	129702914..129703989	0	CX{7}CX{13}CCX{8}CXCX{23}CX{6}C	32	69	7214.41	10.28	S	SbLTP2.5	OsLTP2.4	AtLTP2.4, AtLTP2.9
ZmLTP2.7	GRMZM2G004909	GRMZM2G004909_P01	6	W	162646836..162647576	0	CX{7}CX{13}CCX{8}CXCX{23}CX{6}C	32	69	7114.24	10.16	S	SbLTP2.5	OsLTP2.4	AtLTP2.4, AtLTP2.9
ZmLTP2.8	GRMZM2G320373	GRMZM2G320373_P01	6	W	162662690..162663517	0	CX{7}CX{13}CCX{9}CXCX{23}CX{6}C	28	70	7370.69	11.61	S	SbLTP2.6	OsLTP2.5	-
ZmLTP2.9	GRMZM2G393150	GRMZM2G393150_P01	8	C	152130321..152131030	0	CX{7}CX{13}CCX{9}CXCX{23}CX{6}C	26	74	7797.14	10.36	S	SbLTP2.4	OsLTP2.2	-
**Type C**															
ZmLTPc1	AC225127.3_FG003	AC225127.3_FGP003	4	C	195060826..195061640	0	CX{9}CX{14}CCX{9}CXCX{12}CX{6}C	23	80	7855.96	8.76	S	SbLTPc2	OsLTPc1	AtLTPc2
ZmLTPc2.1	GRMZM2G073377	GRMZM2G073377_P01	7	W	141644107..141645231	0	CX{9}CX{19}CCX{9}CXCX{12}CX{6}C	41	68	6854.02	9.98	S	SbLTPc1	OsLTPc2	-
ZmLTPc2.2		GRMZM2G073377_P04	7	W	141644109..141644635	0	CX{9}CX{19}CCX{9}CXCX{12}CX{6}C	41	78	7922.28	10.02	S	SbLTPc1	OsLTPc2	-
**Type D**															
ZmLTPd1	GRMZM2G031102	GRMZM2G031102_P01	1	C	31896751..31897513	0	CX{14}CX{14}CCX{11}CXCX{24}CX{10}C	20	91	9301.72	9.4	S	SbLTPd9, SbLTPd11, SbLTPd12	OsLTPd5, OsLTPd6, OsLTPd8	AtLTPd7,AtLTPd8
ZmLTPd2	GRMZM2G071771	GRMZM2G071771_P01	2	W	10823770..10824501	0	CX{9}CX{16}CCX{9}CXCX{26}CX{7}C	26	77	8046.4	10.4	S	SbLTPd5	OsLTPd4	-
ZmLTPd3.1	GRMZM2G136364	GRMZM2G136364_P01	2	C	55687675..55688855	0	CX{14}CX{14}CCX{12}CXCX{24}CX{10}C	16	103	10110.55	9.51	S	SbLTPd9, SbLTPd11, SbLTPd12	OsLTPd5, OsLTPd6, OsLTPd8	AtLTPd7,AtLTPd8
ZmLTPd3.2		GRMZM2G136364_P02	2	C	55687675..55688855	0	CX{14}CX{14}CCX{12}CXCX{24}CX{10}C	16	103	10215.73	8.99	S	SbLTPd9, SbLTPd11, SbLTPd12	OsLTPd5, OsLTPd6, OsLTPd8	AtLTPd7,AtLTPd8
ZmLTPd4	GRMZM2G099867	GRMZM2G099867_P01	2	W	127959950..127960704	0	CX{14}CX{14}CCX{11}CXCX{24}CX{10}C	20	91	9301.72	9.4	S	SbLTPd9, SbLTPd11, SbLTPd12	OsLTPd5, OsLTPd6, OsLTPd8	AtLTPd7,AtLTPd8
ZmLTPd5	GRMZM2G164440	GRMZM2G164440_P01	2	C	156327934..156329194	0	CX{10}CX{17}CCX{9}CXCX{24}CX{7}C	-	-	-	-	-	SbLTPd4	OsLTPd3	AtLTPd1,AtLTPd2
ZmLTPd6	GRMZM2G087413	GRMZM2G087413_P01	3	W	138623892..138624577	1	CX{10}CX{17}CCX{9}CXCX{22}CX{9}C	20	87	9889.67	9.27	S	SbLTPd7, SbLTPd8, SbLTPd13	OsLTPd9, OsLTPd10, OsLTPd13	AtLTPd10
ZmLTPd7	GRMZM2G396418	GRMZM2G396418_P01	3	C	162372093..162372586	0	CX{10}CX{18}CCX{9}CXCX{22}CX{8}C	28	78	8282.39	9.69	S	-	-	-
ZmLTPd8	GRMZM2G065557	GRMZM2G065557_P01	3	W	179414637..179416401	0	CX{14}CX{14}CCX{11}CXCX{24}CX{10}C	27	91	9360.23	11.37	S	SbLTPd9, SbLTPd11, SbLTPd12	OsLTPd5, OsLTPd6, OsLTPd8	AtLTPd7,AtLTPd8
ZmLTPd9.1	GRMZM2G094632	GRMZM2G094632_P02	5	W	39411646..39412216	0	CX{10}CX{16}CCX{11}CXCX{23}CX{7}C	24	101	11106.72	5.7	S	SbLTPd1	OsLTPd11	-
ZmLTPd9.2		GRMZM2G094632_P01	5	W	39411646..39412902	0	CX{10}CX{16}CCX{11}CXCX{23}CX{7}C	24	92	9939.33	6.82	S	SbLTPd1	OsLTPd11	-
ZmLTPd10	AC233926.1_FG002	AC233926.1_FGP002	7	W	30954067..30954378	0	CX{9}CX{16}CCX{9}CXCX{26}CX{7}C	26	77	8046.4	10.4	S	SbLTPd5	OsLTPd4	-
ZmLTPd11	GRMZM2G000221	GRMZM2G000221_P01	7	W	31086409..31087079	0	CX{9}CX{16}CCX{9}CXCX{26}CX{7}C	27	78	8153.49	6.77	S	SbLTPd6	-	-
ZmLTPd12	GRMZM2G471051	GRMZM2G471051_P01	8	C	162752046..162752849	0	CX{9}CX{16}CCX{9}CXCX{21}C{8}C	29	78	8162.44	7.98	S	-	OsLTPd2	-
ZmLTPd13	GRMZM2G170969	GRMZM2G170969_P01	8	C	162756043..162756672	0	CX{10}CX{11}CCX{9}CXCX{22}CX{6}C	29	71	7437.4	9.19	S	-	-	-
ZmLTPd14	GRMZM2G091054	GRMZM2G091054_P01	8	C	171565757..171566487	1	CX{10}CX{17}CCX{9}CXCX{22}CX{9}C	24	87	9554.06	6.78	S	SbLTPd7, SbLTPd8, SbLTPd13	OsLTPd9, OsLTPd10, OsLTPd13	AtLTPd10
ZmLTPd15	GRMZM2G155555	GRMZM2G155555_P01	10	C	96605920..96606680	1	CX{10}CX{15}CCX{9}CXCX{22}CX{9}C	27	93	10346.2	9.84	S	-	OsLTPd14	-
ZmLTPd16	GRMZM2G063375	GRMZM2G063375_P01	10	C	96617537..96618949	1	CX{10}CX{15}CCX{9}CXCX{22}CX{9}C	27	93	10302.08	9.46	S	-	OsLTPd14	-
**Type G**															
ZmLTPg1.1		GRMZM5G850455_P01	1	W	14103040..14104304	0	CX{9}CX{14}CCX{12}CXCX{26}CX{9}C	23	188	19357.29	9.13	S	SbLTPg8, SbLTPg12	OsLTPg2, OsLTPg19	AtLTPg6, AtLTPg13, AtLTPg14
ZmLTPg1.2	GRMZM5G850455	GRMZM5G850455_P02	1	W	14103040..14104361	2	CX{9}CX{14}CCX{12}CXCX{26}CX{9}C	23	169	16940.38	8.21	S	SbLTPg8, SbLTPg12	OsLTPg2, OsLTPg19	AtLTPg6, AtLTPg13, AtLTPg14
ZmLTPg1.3		GRMZM5G850455_P03	1	W	14103040..14104361	1	CX{9}CX{14}CCX{12}CXCX{26}CX{9}C	23	224	22784.06	8.96	S	SbLTPg8, SbLTPg12	OsLTPg2, OsLTPg19	AtLTPg6, AtLTPg13, AtLTPg14
ZmLTPg2	GRMZM2G174680	GRMZM2G174680_P01	1	C	52625970..52627201	1	CX{9}CX{16}CCX{13}CXCX{21}CX{9}C	29	160	15631.12	6.38	S	SbLTPg6	OsLTPg4	-
ZmLTPg3	GRMZM2G078876	GRMZM2G078876_P01	1	C	66023613..66026784	2	CX{9}CX{16}CCX{14}CXCX{25}CX{9}C	25	156	15026.19	8.71	S	SbLTPg5	OsLTPg5, AtLTPg10	-
ZmLTPg4	GRMZM2G083725	GRMZM2G083725_P01	1	C	194657095..194659202	2	CX{9}CX{14}CCX{12}CXCX{29}CX{9}C	23	158	15977.04	6.97	S	-	-	-

ZmLTPg5	GRMZM2G097137	GRMZM2G097137_P01	1	W	263198227..263201072	1	CX{9}CX{15}CCX{12}CXCX{27}CX{9}C	28	133	12510.03	8.38	S	SbLTPg3	-	-
ZmLTPg6	GRMZM2G006047	GRMZM2G006047_P02	1	C	287110810..287111891	2	CX{9}CX{14}CCX{12}CXCX{26}CX{9}C	32	150	14309.15	9.87	S	SbLTPx1, SbLTPg9,	OsLTPg7, OsLTPg14, OsLTPg16, OsLTPg18	AtLTPg15, AtLTPg19, AtLTPG26
ZmLTPg7	GRMZM2G005991	GRMZM2G005991_P01	1	C	287146113..287147400	3	CX{10}CX{17}CCX{12}CXCX{24}CX{8}C	27	174	17391.17	8.63	S	SbLTPg4	OsLTPg8	AtLTPg16, AtLTPg20
ZmLTPg8.1	GRMZM2G065640	GRMZM2G065640_P01	1	W	287188063..287188991	2	CX{9}CX{14}CCX{12}CXCX{25}CX{9}C	24	175	17239.55	8.21	S	SbLTPg2	OsLTPg9	-
ZmLTPg8.2		GRMZM2G065640_P02	1	W	287188063..287188991	2	CX{9}CX{14}CCX{12}CXCX{25}CX{9}C	24	158	15377.38	4.66	S	SbLTPg2	OsLTPg9	-
ZmLTPg9	GRMZM2G168833	GRMZM2G168833_P01	1	W	289786005..289788499	2	CX{9}CX{16}CCX{12}CXCX{26}CX{8}C	-	-	-	-	-	SbLTPg1	OsLTPg10	-
ZmLTPg10	GRMZM2G414620	GRMZM2G414620_P01	2	C	41484533..41485576	1	CX{9}CX{14}CCX{12}CXCX{24}CX{10}C	35	180	18249.23	9.95	S	SbLTPg19	-	-
ZmLTPg11	GRMZM2G151021	GRMZM2G151021_P01	2	W	47422297..47424522	2	CX{12}CX{8}CCX{14}CXCX{24}CX{13}C	27	190	19051.12	6.55	S	SbLTPg18	OsLTPg29	-
ZmLTPg12	AC194203.3_FG003	AC194203.3_FGP003	2	C	212487102..212487524	0	CX{9}CX{16}CCX{14}CXCX{22}CX{9}C	22	118	11730.54	7.99	S	SbLTPg13	OsLTPg21	-
ZmLTPg13	GRMZM2G176347	GRMZM2G176347_P01	2	C	233632142..233633151	2	CX{10}CX{18}CCX{12}CXCX{24}CX{8}C	25	170	16664.09	11.1	S	SbLTPg11	OsLTPg17	-
ZmLTPg14	GRMZM2G071575	GRMZM2G071575_P01	4	C	45262758..45263990	2	CX{9}CX{14}CCX{12}CXCX{29}CX{9}C	25	160	16219.39	6.97	S	SbLTPg21	OsLTPg22	AtLTPg4
ZmLTPg15	GRMZM2G145054	GRMZM2G145054_P01	4	W	126473476..126474249	1	C{6}CX{13}CCX{12}CXCX{25}CX{5}C	-	-	-	-	-	SbLTPg16	-	-
ZmLTPg16	GRMZM2G130454	GRMZM2G130454_P03	5	W	4429841..4430924	2	CX{9}CX{14}CCX{12}CXCX{41}CX{20}C	32	160	16243.38	11.34	S	-	-	-
ZmLTPg17.1	GRMZM2G141858	GRMZM2G141858_P01	5	W	49545870..49548495	2	CX{6}CX{13}CCX{12}CXCX{25}CX{8}C	25	124	12692.44	6.93	S	SbLTPg23	OsLTPg13	AtLTPg7
ZmLTPg17.2		GRMZM2G141858_P02	5	W	49545872..49548469	1	CX{6}CX{13}CCX{12}CXCX{25}CX{8}C	25	115	11827.49	6.93	S	SbLTPg23	OsLTPg13	AtLTPg7
ZmLTPg18	GRMZM2G116167	GRMZM2G116167_P01	5	W	59978801..59980128	2	CX{6}CX{15}CCX{12}CXCX{25}CX{8}C	26	149	14486.34	10.8	S	SbLTPg24	OsLTPg24	-
ZmLTPg19	GRMZM2G089400	GRMZM2G089400_P01	6	C	89065818..89068077	2	CX{5}CX{13}CCX{12}CXCX{25}CX{8}C	25	124	12805.72	8.27	S	SbLTPg23	OsLTPg13	AtLTPg7
ZmLTPg20.1	GRMZM2G089288	GRMZM2G089288_P03	7	W	8901782..8903227	2	CX{9}CX{14}CCX{12}CXCX{25}CX{9}C	46	140	13977	4.27	S	-	OsLTPg15	-
ZmLTPg20.2		GRMZM2G089288_P01	7	W	8901856..8903227	2	CX{9}CX{14}CCX{12}CXCX{25}CX{9}C	29	140	13977	4.27	S	-	OsLTPg15	-
ZmLTPg21	GRMZM2G046750	GRMZM2G046750_P01	7	W	9201072..9202357	2	CX{10}CX{14}CCX{12}CXCX{24}CX{8}C	33	187	17984.37	5.04	C	SbLTPg10	-	-
ZmLTPg22	GRMZM2G170044	GRMZM2G170044_P01	7	W	16096163..16099019	2	CX{9}CX{16}CCX{12}CXCX{26}CX{8}C	28	178	17674.11	9.05	S	SbLTPg8, SbLTPg12	OsLTPg2, OsLTPg19	AtLTPg6, AtLTPg13, AtLTPg14
ZmLTPg23	GRMZM2G171597	GRMZM2G171597_P01	7	C	167020090..167021470	2	CX{9}CX{16}CCX{14}CXCX{22}CX{9}C	24	145	14127.38	10.37	S	SbLTPg13	OsLTPg21	-
ZmLTPg24	GRMZM2G379035	GRMZM2G379035_P01	8	C	171016014..171017268	2	CX{9}CX{16}CCX{12}CXCX{25}CX{9}C	27	167	16275.83	5.14	S	SbLTPg15, SbLTPg22	OsLTPg1, OsLTPg12	AtLTPg21
ZmLTPg25	GRMZM2G004466	GRMZM2G004466_P01	9	W	149245864..149247159	2	CX{9}CX{16}CCX{12}CXCX{24}CX{9}C	28	175	17097.42	8.13	S	SbLTPg7	OsLTPg3	AtLTPg11, AtLTPg31
ZmLTPg26	GRMZM2G140646	GRMZM2G140646_P01	10	C	15003990..15007027	2	CX{9}CX{21}CCX{18}CXCX{24}CX{20}C	51	183	18124.19	5.51	S	SbLTPg20	OsLTPg28	-
**Single**															-
ZmLTPx1.1		GRMZM2G036063_P01	7	W	45409454..45410733	1	CX{9}CX{16}CCX{13}CXCX{26}CX{12}C	24	144	14941.3	4.87	S	SbLTPx3	-	-
ZmLTPx1.2	GRMZM2G036063	GRMZM2G036063_P02	7	W	45409455..45410645	1	CX{9}CX{16}CCX{13}CXCX{26}CX{12}C	24	145	15097.49	5.14	S	SbLTPx3	-	-
ZmLTPx1.3		GRMZM2G036063_P03	7	W	45409465..45410441	0	CX{9}CX{16}CCX{13}CXCX{26}CX{12}C	24	130	13711.83	6.68	S	SbLTPx3	-	-
ZmLTPx2	GRMZM2G166484	GRMZM2G166484_P01	10	C	118762389..118764275	2	CX{12}CX{8}CCX{14}CXCX{24}CX{13}C	21	170	17367.16	11.39	S	-	-	-
