# Supplementary material for: Non-specific lipid transfer proteins in maize
Source: BMC Plant Biol. 2014 Oct 28;14:281. doi: 10.1186/s12870-014-0281-8 (PMC4226865; doi:10.1186/s12870-014-0281-8)
Supplement: Additional file 3: Table S3. — Designations used for nsLTP sequences in this report, and their corresponding database accession numbers. [file 12870_2014_281_MOESM3_ESM.pdf]

Table S3. Designations used for nsLTP sequences in this report, and their corresponding database accession numbers.

Arabidopsis			Rice			Sorghum		
**Name**	**Locus**	**Group**	**Name**	**Locus**	**Group**	**Name**	**Locus**	**Group**
AtLTP1.1	AT2G15050.3	Type 1	OsLTP1.1	LOC_Os01g12020.1	Type 1	SbLTP1.1	Sb03g001580.1	Type 1
AtLTP1.2	AT2G15325.1	Type 1	OsLTP1.2	LOC_Os01g60740.1	Type 1	SbLTP1.2	Sb03g038280.1	Type 1
AtLTP1.3	AT2G18370.1	Type 1	OsLTP1.3	LOC_Os03g59380.1	Type 1	SbLTP1.3	Sb07g002600.1	Type 1
AtLTP1.4	AT2G38530.1	Type 1	OsLTP1.4	LOC_Os05g40010.1	Type 1	SbLTP1.4	Sb08g002660.1	Type 1
AtLTP1.5	AT2G38540.1	Type 1	OsLTP1.5	LOC_Os06g06340.1	Type 1	SbLTP1.5	Sb08g002670.1	Type 1
AtLTP1.6	AT3G08770.1	Type 1	OsLTP1.6	LOC_Os06g34840.1	Type 1	SbLTP1.6	Sb08g002680.1	Type 1
AtLTP1.7	AT3G51590.1	Type 1	OsLTP1.7	LOC_Os08g03690.1	Type 1	SbLTP1.7	Sb08g002690.1	Type 1
AtLTP1.8	AT3G51600.1	Type 1	OsLTP1.9	LOC_Os11g02350.1	Type 1	SbLTP1.8	Sb08g002700.1	Type 1
AtLTP1.9	AT4G33355.1	Type 1	OsLTP1.12	LOC_Os11g02400.1	Type 1	SbLTP1.9	Sb10g021170.1	Type 1
AtLTP1.10	AT5G01870.1	Type 1	OsLTP1.13	LOC_Os11g02424.2	Type 1	SbLTP2.1	Sb01g017700.1	Type 2
AtLTP1.11	AT5G59310.1	Type 1	OsLTP1.14	LOC_Os11g24070.1	Type 1	SbLTP2.2	Sb01g017710.1	Type 2
AtLTP1.12	AT5G59320.1	Type 1	OsLTP1.16	LOC_Os12g02300.1	Type 1	SbLTP2.3	Sb01g049830.1	Type 2
AtLTP1.13	AT4G28395.1	Type 1	OsLTP1.17	LOC_Os12g02310.1	Type 1	SbLTP2.4	Sb03g031720.1	Type 2
AtLTP2.2	AT1G43666.1	Type 2	OsLTP1.18	LOC_Os12g02320.1	Type 1	SbLTP2.5	Sb09g027590.1	Type 2
AtLTP2.3	AT1G43667.1	Type 2	OsLTP1.19	LOC_Os12g02330.2	Type 1	SbLTP2.6	Sb09g027600.1	Type 2
AtLTP2.4	AT1G48750.1	Type 2	OsLTP1.20	LOC_Os12g02340.1	Type 1	SbLTP2.7	Sb10g029330.1	Type 2
AtLTP2.5	AT1G66850.1	Type 2	OsLTP1.21	LOC_Os11g02369.1	Type 1	SbLTPc1	Sb02g030420.1	Type C
AtLTP2.6	AT1G73780.1	Type 2	OsLTP1.22	LOC_Os11g02389.1	Type 1	SbLTPc2	Sb07g025160.1	Type C
AtLTP2.7	AT2G14846.1	Type 2	OsLTP2.1	LOC_Os01g49640.1	Type 2	SbLTPd1	Sb01g026220.1	Type D
AtLTP2.8	AT3G12545	Type 2	OsLTP2.2	LOC_Os01g49650.1	Type 2	SbLTPd2	Sb01g026225.1	Type D
AtLTP2.9	AT3G18280.1	Type 2	OsLTP2.3	LOC_Os03g02050.1	Type 2	SbLTPd3	Sb01g026230.1	Type D
AtLTP2.10	AT3G57310.1	Type 2	OsLTP2.4	LOC_Os05g47700.1	Type 2	SbLTPd4	Sb02g009300.1	Type D
AtLTP2.11	AT5G38160.1	Type 2	OsLTP2.5	LOC_Os05g47730.1	Type 2	SbLTPd5	Sb02g009320.1	Type D
AtLTP2.12	AT5G38170.1	Type 2	OsLTP2.6	LOC_Os06g49190.1	Type 2	SbLTPd6	Sb02g009340.1	Type D
AtLTP2.13	AT5G38180.1	Type 2	OsLTP2.7	LOC_Os10g36070.1	Type 2	SbLTPd7	Sb03g037210.1	Type D
AtLTP2.14	AT5G38195.1	Type 2	OsLTP2.8	LOC_Os10g36090.1	Type 2	SbLTPd8	Sb03g037220.1	Type D
AtLTPc1	AT5G07230.1	Type C	OsLTP2.9	LOC_Os10g36100.1	Type 2	SbLTPd9	Sb03g039880.1	Type D
AtLTPc2	AT5G52160.1	Type C	OsLTP2.10	LOC_Os10g36110.1	Type 2	SbLTPd10	Sb03g043610.1	Type D
AtLTPc3	AT5G62080.1	Type C	OsLTP2.11	LOC_Os10g36160.1	Type 2	SbLTPd11	Sb06g016160.1	Type D
AtLTPd1	AT5G48485.1	Type D	OsLTP2.12	LOC_Os10g36170.1	Type 2	SbLTPd12	Sb06g016170.1	Type D
AtLTPd2	AT5G48490.1	Type D	OsLTP2.13	LOC_Os11g40530.1	Type 2	SbLTPd13	Sb08g005360.1	Type D
AtLTPd3	AT5G55410.1	Type D	OsLTPc1	LOC_Os08g43290.1	Type C	SbLTPg1	Sb01g004610.1	Type G
AtLTPd4	AT5G55450.1	Type D	OsLTPc2	LOC_Os09g35700.1	Type C	SbLTPg2	Sb01g005410.1	Type G
AtLTPd5	AT5G55460.1	Type D	OsLTPd1	LOC_Os01g68580.1	Type D	SbLTPg3	Sb01g011270.1	Type G
AtLTPd6	AT2G37870.1	Type D	OsLTPd2	LOC_Os01g68589.1	Type D	SbLTPg4	Sb01g005420.1	Type G
AtLTPd7	AT3G53980.1	Type D	OsLTPd3	LOC_Os07g18750.1	Type D	SbLTPg5	Sb01g033830.1	Type G
AtLTPd8	AT5G05960.1	Type D	OsLTPd4	LOC_Os07g18990.1	Type D	SbLTPg6	Sb01g036750.1	Type G
AtLTPd9	AT1G32280.1	Type D	OsLTPd5	LOC_Os01g62980.1	Type D	SbLTPg7	Sb01g044330.1	Type G
AtLTPd10	AT4G30880.1	Type D	OsLTPd6	LOC_Os04g33920.1	Type D	SbLTPg8	Sb01g046080.1	Type G
AtLTPd11	AT4G33550.2	Type D	OsLTPd7	LOC_Os04g33930.2	Type D	SbLTPg9	Sb02g004130.1	Type G

AtLTPd12	AT5G56480.1	Type D	OsLTPd8	LOC_Os05g06780.1	Type D	SbLTPg10	Sb02g004200.1	Type G
AtLTPe1	AT3G07450.1	Type E	OsLTPd9	LOC_Os01g58650.1	Type D	SbLTPg11	Sb02g004210.1	Type G
AtLTPe2	AT3G52130.1	Type E	OsLTPd10	LOC_Os01g58660.1	Type D	SbLTPg12	Sb02g005900.1	Type G
AtLTPg1	AT1G03103.1	Type G	OsLTPd11	LOC_Os10g05720.2	Type D	SbLTPg13	Sb02g039860.1	Type G
AtLTPg2	AT1G05450.2	Type G	OsLTPd12	LOC_Os11g29420.1	Type D	SbLTPg14	Sb02g041710.1	Type G
AtLTPg3	AT1G18280.1	Type G	OsLTPd13	LOC_Os03g25350.1	Type D	SbLTPg15	Sb03g037810.1	Type G
AtLTPg4	AT1G27950.1	Type G	OsLTPd14	LOC_Os11g03870.1	Type D	SbLTPg16	Sb04g024055.1	Type G
AtLTPg5	AT1G36150.1	Type G	OsLTPg1	LOC_Os01g59870.1	Type G	SbLTPg17	Sb05g022630.1	Type G
AtLTPg6	AT1G55260.1	Type G	OsLTPg2	LOC_Os03g07100.1	Type G	SbLTPg18	Sb06g017510.1	Type G
AtLTPg7	AT1G62790.1	Type G	OsLTPg3	LOC_Os03g09230.1	Type G	SbLTPg19	Sb06g019070.1	Type G
AtLTPg8	AT1G73550.1	Type G	OsLTPg4	LOC_Os03g20760.1	Type G	SbLTPg20	Sb06g020910.1	Type G
AtLTPg9	AT1G73560.1	Type G	OsLTPg5	LOC_Os03g26800.1	Type G	SbLTPg21	Sb07g026120.1	Type G
AtLTPg10	AT1G73890.1	Type G	OsLTPg6	LOC_Os03g26820.1	Type G	SbLTPg22	Sb09g023870.1	Type G
AtLTPg11	AT2G13820.1	Type G	OsLTPg7	LOC_Os03g57970.1	Type G	SbLTPg23	Sb10g027810.1	Type G
AtLTPg12	AT2G27130.1	Type G	OsLTPg8	LOC_Os03g57980.1	Type G	SbLTPg24	Sb10g029740.1	Type G
AtLTPg13	AT2G44290.1	Type G	OsLTPg9	LOC_Os03g57990.1	Type G	SbLTPx1	Sb01g005430.1	single
AtLTPg14	AT2G44300.1	Type G	OsLTPg10	LOC_Os03g58940.1	Type G	SbLTPx2	Sb02g011135.1	single
AtLTPg15	AT2G48130.1	Type G	OsLTPg11	LOC_Os04g38840.1	Type G	SbLTPx3	Sb02g011205.1	single
AtLTPg16	AT2G48140.1	Type G	OsLTPg12	LOC_Os05g41030.1	Type G			
AtLTPg19	AT3G22600.1	Type G	OsLTPg13	LOC_Os06g47200.1	Type G			
AtLTPg20	AT3G22620.1	Type G	OsLTPg14	LOC_Os07g07790.1	Type G			
AtLTPg21	AT3G43720.1	Type G	OsLTPg15	LOC_Os07g07870.1	Type G			
AtLTPg22	AT3G58550.1	Type G	OsLTPg16	LOC_Os07g07860.1	Type G			
AtLTPg23	AT4G08670.1	Type G	OsLTPg17	LOC_Os07g07920.1	Type G			
AtLTPg24	AT4G12360.1	Type G	OsLTPg18	LOC_Os07g07930.1	Type G			
AtLTPg25	AT4G14805.1	Type G	OsLTPg19	LOC_Os07g09970.1	Type G			
AtLTPG26	AT4G14815.1	Type G	OsLTPg20	LOC_Os07g30590.1	Type G			
AtLTPG27	AT4G22630.1	Type G	OsLTPg21	LOC_Os07g43290.1	Type G			
AtLTPG28	AT4G22666.1	Type G	OsLTPg22	LOC_Os08g42040.1	Type G			
AtLTPG29	AT5G09370.1	Type G	OsLTPg23	LOC_Os11g37320.1	Type G			
AtLTPG30	AT5G13900.1	Type G	OsLTPg24	LOC_Os06g49770.1	Type G			
AtLTPG31	AT5G64080.1	Type G	OsLTPg25	LOC_Os11g37280.1	Type G			
AtLTPg17	AT3G22570.1	single	OsLTPg28	LOC_Os01g42210.1	Type G			
AtLTPg18	AT3G22580.1	single	OsLTPg29	LOC_Os03g46110.1	Type G			
AtLTPG33	AT4G22640.1	single	OsLTPx1	LOC_Os03g44000.1	single			
AtLTPG34	AT4G22650.1	single	OsLTPx2	LOC_Os07g27940.1	single			
AtLTPx1	AT1G52415.1	single	OsLTPx3	LOC_Os11g34660.1	single			
AtLTPx2	AT1G64235.1	single						
AtLTPx3	AT4G08530.1	single						
