# Supplementary material for: Non-specific lipid transfer proteins in maize
Source: BMC Plant Biol. 2014 Oct 28;14:281. doi: 10.1186/s12870-014-0281-8 (PMC4226865; doi:10.1186/s12870-014-0281-8)
Supplement: Additional file 4: Table S4. — Sorghum genes encoding proteins with a Pfam domain PF00234 which belong to hybrid proline-rich proteins, alpha-amylase/trypsin inhibitors, prolamin storage proteins and 2S albumin storage proteins. [file 12870_2014_281_MOESM4_ESM.pdf]

Table S4. Sorghum genes encoding proteins with a Pfam domain PF00234 which belong to hybrid proline-rich proteins, alpha-amylase/trypsin inhibitors, prolamin storage proteins and 2S albumin storage proteins.

**hybrid proline-rich proteins**			
Sb02g032910	Sb02g032920	Sb02g037735	Sb02g037740
Sb09g024070	Sb02g041700	Sb05g000960	Sb02g011200
Sb02g011170	Sb02g011190	Sb04g032620	Sb04g032650
Sb06g024800	Sb10g025210	Sb01g029450	Sb10g000510
Sb06g028200	Sb01g029630	Sb01g029620	Sb06g030490
Sb05g003860	Sb01g009990	Sb06g030500	Sb10g004790
Sb01g050420	Sb06g024770	Sb04g032640	Sb04g032670
Sb01g029610	Sb04g032660	Sb01g050360	Sb04g032680
Sb06g024780	Sb06g024790		
**alpha-amylase/trypsin inhibitors**			
Sb02g006470	Sb02g006550	Sb02g006480	Sb02g002495
Sb02g006570	Sb09g024570	Sb02g006560	Sb02g002500
**prolamin storage proteins**			
Sb02g025490	Sb02g025510	Sb09g000360	
**2S albumin storage proteins**			
Sb02g004220	Sb01g012630	Sb01g012620	
