# Supplementary material for: Non-specific lipid transfer proteins in maize
Source: BMC Plant Biol. 2014 Oct 28;14:281. doi: 10.1186/s12870-014-0281-8 (PMC4226865; doi:10.1186/s12870-014-0281-8)
Supplement: Additional file 5: Table S5. — A summary of nsLTP genes in maize, sorghum, rice and Arabidopsis. [file 12870_2014_281_MOESM5_ESM.pdf]

Table S5. A summary of nsLTP genes in maize, sorghum, rice and Arabidopsis.

Group	*Zea mays*	*Sorghum bicolor*	*Oryza sativa*	*A. thaliana*
Type 1	8	9	18	13
Type 2	9	7	13	13
Type C	2	2	2	3
Type D	16	13	14	12
Type E	0	0	0	2
Type G	26	24	27	29
single	2	3	3	7
Tota	63	58	77	79
