# Supplementary material for: Non-specific lipid transfer proteins in maize
Source: BMC Plant Biol. 2014 Oct 28;14:281. doi: 10.1186/s12870-014-0281-8 (PMC4226865; doi:10.1186/s12870-014-0281-8)
Supplement: Additional file 7: Table S6. — Predicted phosphorylation sites in ZmLTPs. [file 12870_2014_281_MOESM7_ESM.pdf]

Table S6. Predicted phosphorylation sites in ZmLTPs.

[1]phosphorylation site.

[2]phosphorylated residue.

Name	Full length	Position[1]	Amino acide[2]	Name	Full length	Position[1]	Amino acid[2]
ZmLTP1.5.2	117	110	theonine	ZmLTPg13	195	59	serine
ZmLTP2.4	96	79	serine			122	serine
ZmLTPd1	111	49	theonine			132	theonine
ZmLTPd4	111	49	theonine	ZmLTPg14	185	144	theonine
ZmLTPg1.1	211	126	serine			150	serine
		183	serine	ZmLTPg15	166	11	serine
ZmLTPg1.2	192	128	serine			17	serine
ZmLTPg1.3	247	126	serine	ZmLTPg16	192	84	serine
		183	serine	ZmLTPg17.1	149	106	serine
ZmLTPg2	189	153	theonine			110	serine
ZmLTPg3	181	148	serine			114	serine
		156	serine	ZmLTPg19	149	106	serine
ZmLTPg5	161	130	theonine			110	serine
ZmLTPg6	182	84	serine			114	serine
		127	theonine	ZmLTPg21	220	131	serine
		130	theonine	ZmLTPg23	169	136	theonine
ZmLTPg7	201	11	serine			148	serine
		162	serine	ZmLTPg24	194	166	serine
ZmLTPg8.1	199	124	serine	ZmLTPg25	203	30	theonine
		158	serine			33	serine
		163	serine			116	theonine
		170	serine			148	serine
		175	serine			150	theonine
ZmLTPg8.2	182	124	serine			155	theonine
		147	theonine			173	serine
		158	serine	ZmLTPg26	234	183	serine
ZmLTPg10	215	178	serine			211	theonine
		185	theonine	ZmLTPx1.2	169	161	serine
ZmLTPg11	217	114	serine	ZmLTPx2	191	141	theonine
		188	theonine				
