# Supplementary figures and images for: Non-specific lipid transfer proteins in maize
Source: BMC Plant Biol. 2014 Oct 28;14:281. doi: 10.1186/s12870-014-0281-8 (PMC4226865; doi:10.1186/s12870-014-0281-8)

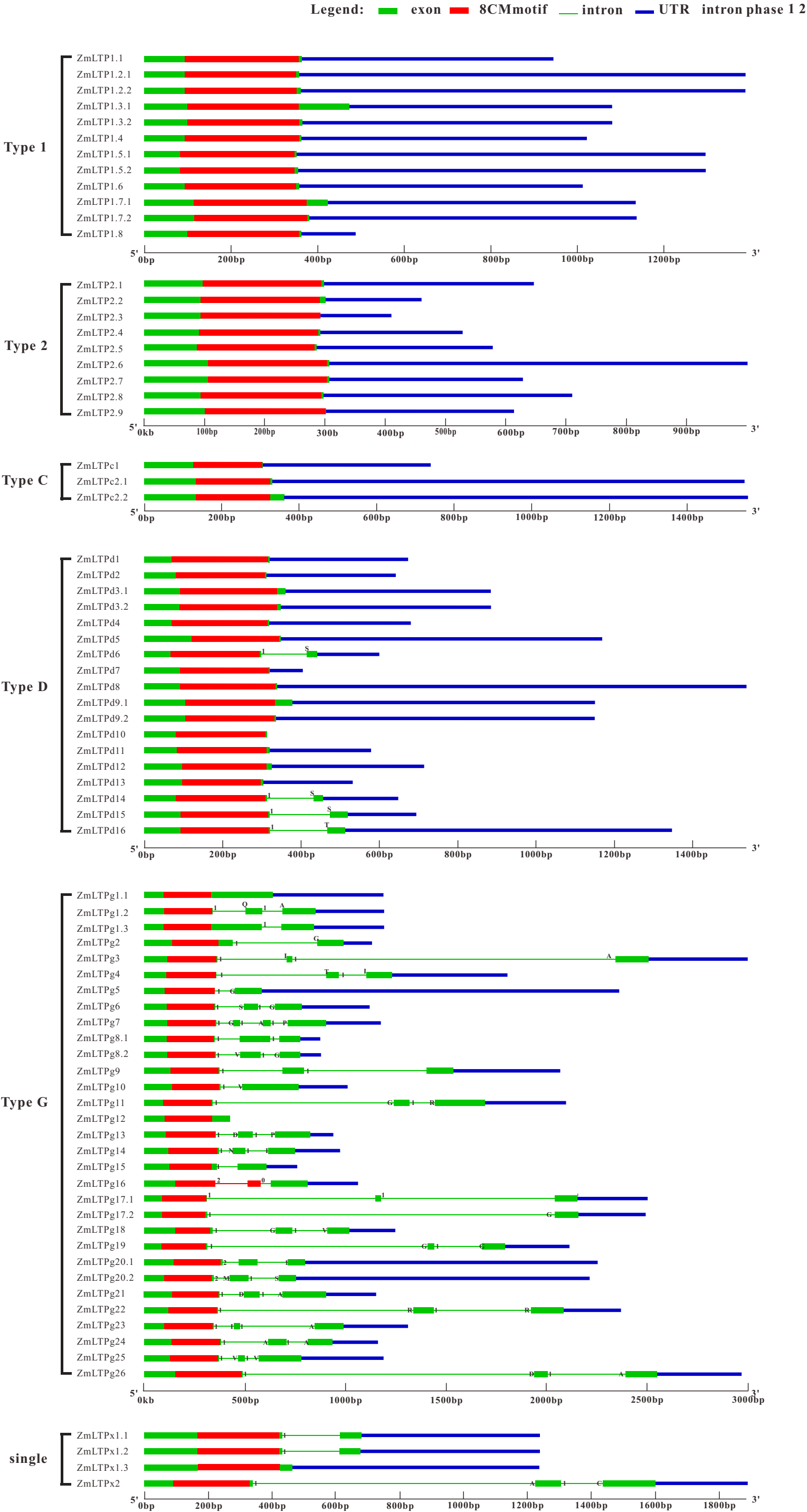

Supplement: Additional file 9: Figure S3. — The map of intron-exon arrangement of maize nsLTP genes. Introns and exons are drawn to scale with the full encoding regions of their respective. Exons are depicted as green boxes, introns as connecting thin lines and 8CM as red boxes. Non-translated regions, when supported by full-length cDNA sequences, are shown in blue boxes. [file 12870_2014_281_MOESM9_ESM.pdf]

Legend: exon 8CMmotif intron UTR intron phase 1 2

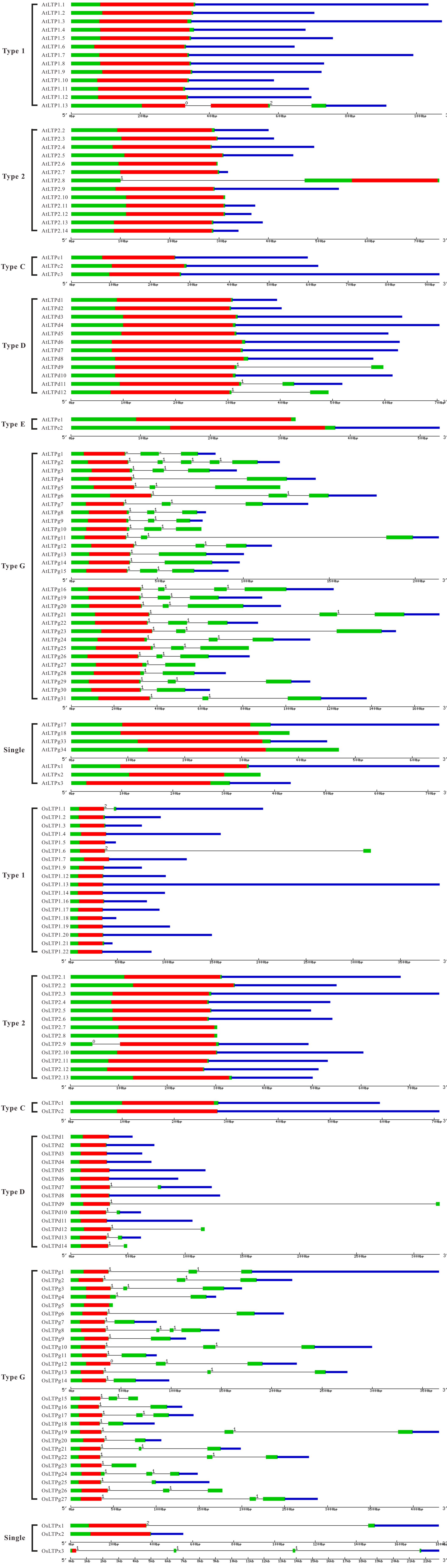

Supplement: Additional file 10: Figure S4. — The map of intron-exon arrangement of Arabidopsis and rice nsLTP genes. Introns and exons are drawn to scale with the full encoding regions of their respective. Exons are depicted as green boxes, introns as connecting thin lines and 8CM as red boxes. Non-translated regions, when supported by full-length cDNA sequences, are shown in blue boxes. [file 12870_2014_281_MOESM10_ESM.pdf]

**log<sub>2.8</sub>-fold change**

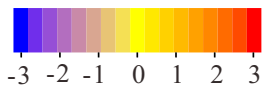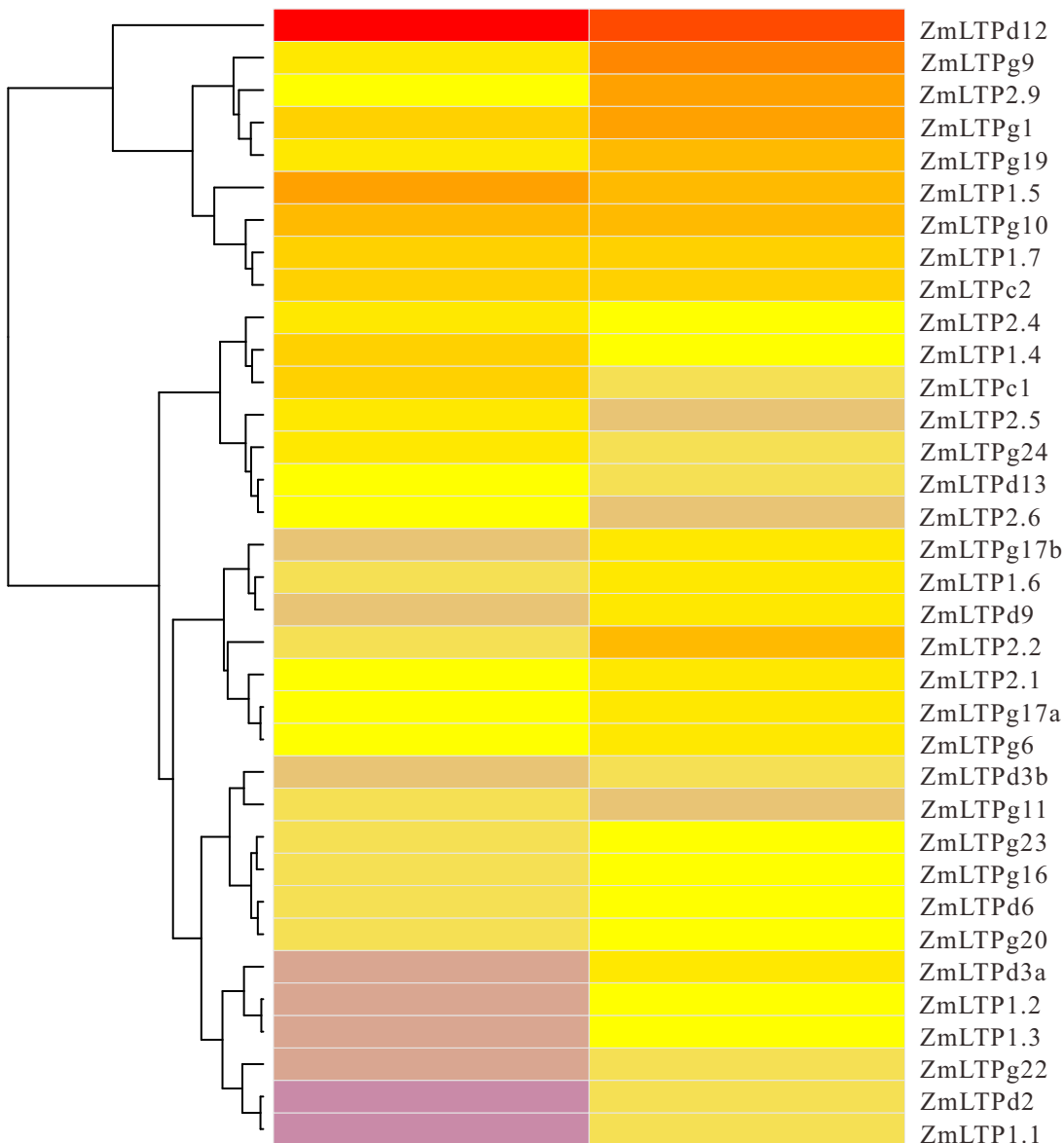

**ETH-DL3**

**ETH-DH7**

Supplement: Additional file 11: Table S7. — Syntenic sites harboring nsLTP genes in maize, sorghum and rice. [file 12870_2014_281_MOESM11_ESM.pdf]

**log<sub>2</sub>-fold change**

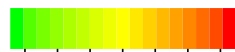

-6 -4 -2 0 2 4 6

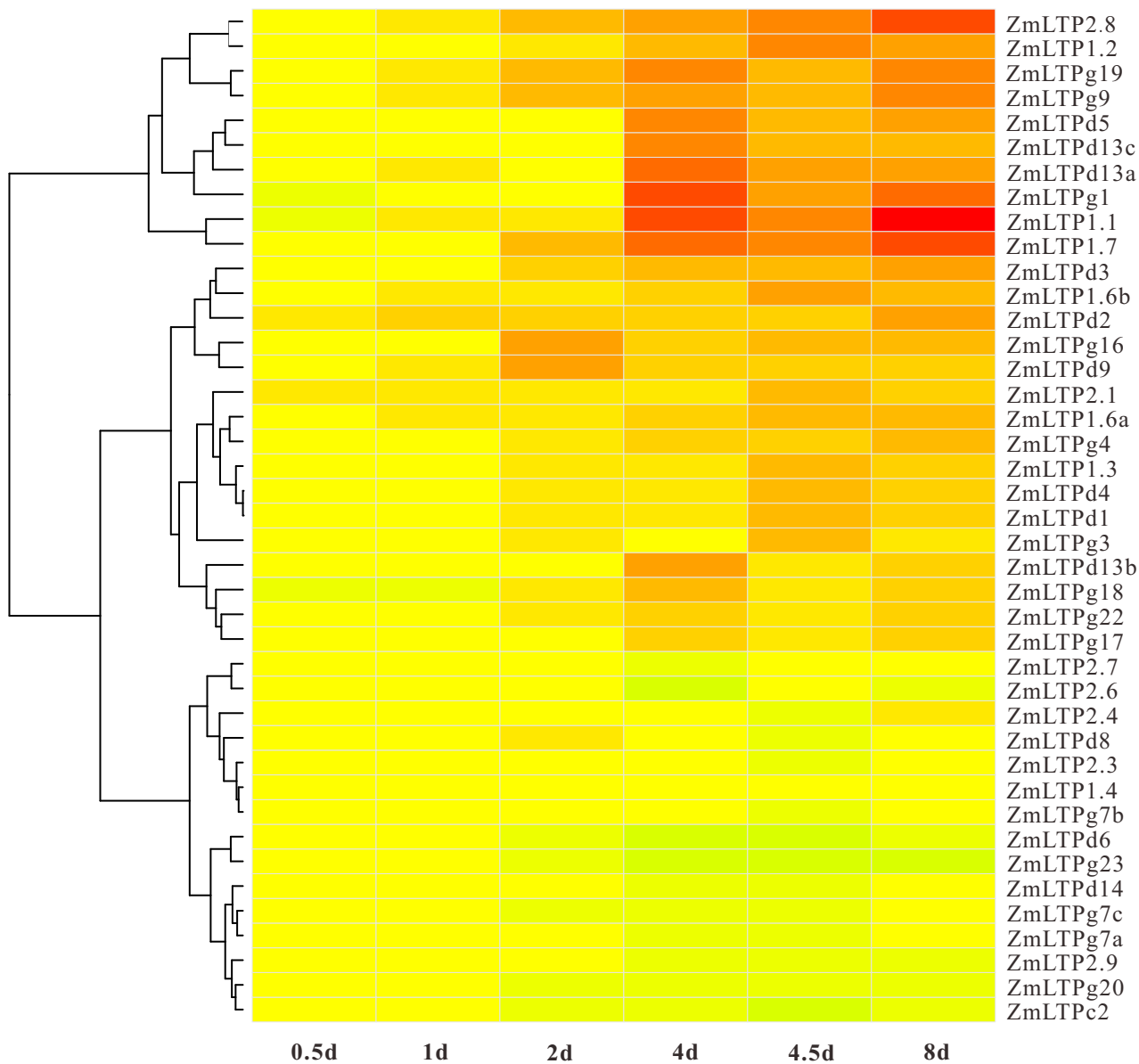

Supplement: Additional file 16: Figure S5. — Normalized signal intensities of 41 probe sets representing 36 ZmLTP genes displayed for the microarray experiment, which was treated with Ustilago maydis. Log2 ratio values (shown by a green-red gradient) represents the fold change of the gene expression in the fungal infections. [file 12870_2014_281_MOESM16_ESM.pdf]

**log<sub>2</sub>-fold change**

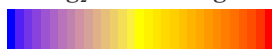

-2      0      2

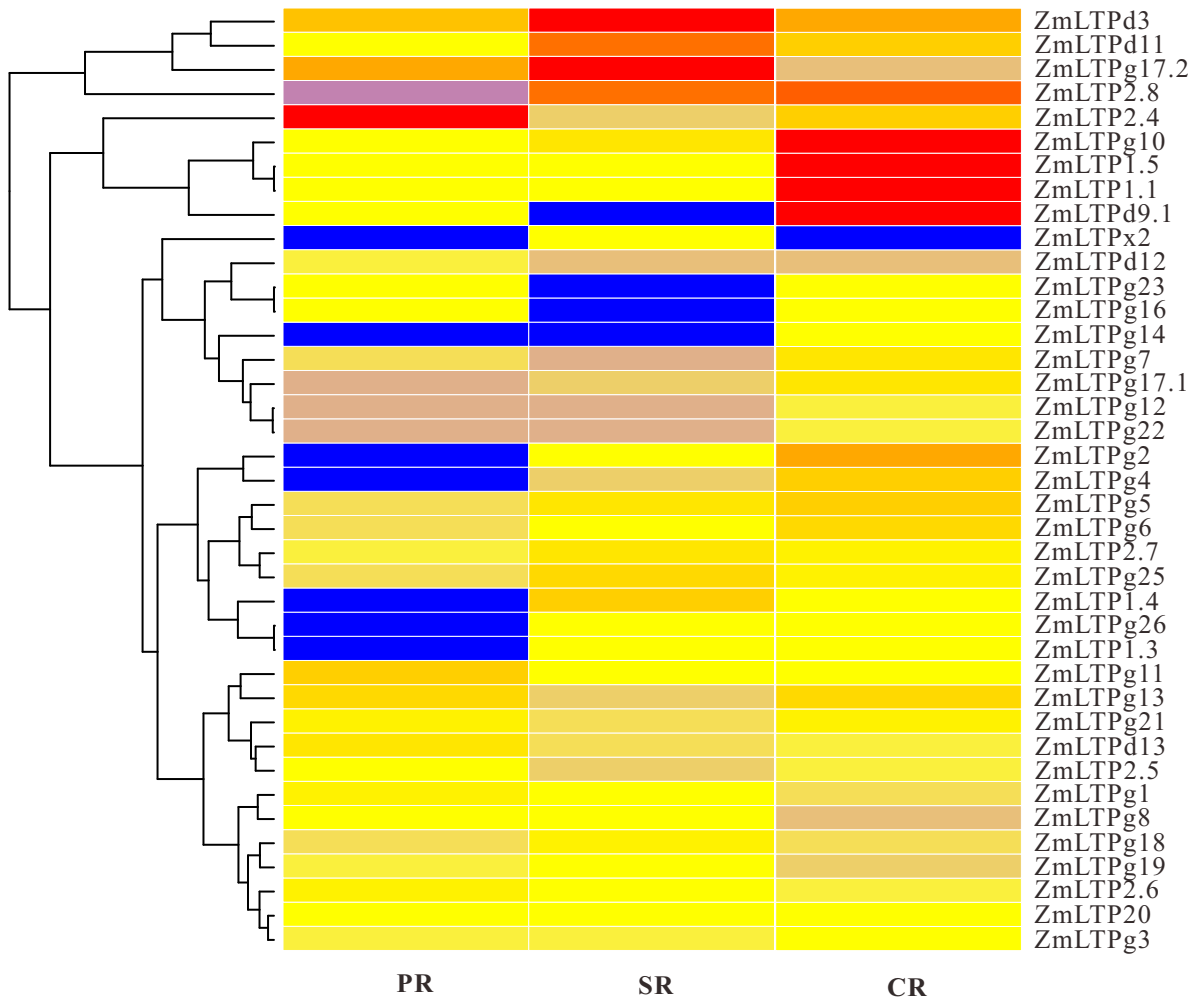

Supplement: Additional file 19: Figure S6. — Expression profiles of 38 ZmLTP genes in three root types [primary root (PR), seminal roots (SR) and crown roots (CR)] under salt stress. [file 12870_2014_281_MOESM19_ESM.pdf]
