# Supplementary material for: Non-specific lipid transfer proteins in maize
Source: BMC Plant Biol. 2014 Oct 28;14:281. doi: 10.1186/s12870-014-0281-8 (PMC4226865; doi:10.1186/s12870-014-0281-8)
Supplement: Additional file 13: Table S9. — Analysis of cis-elements in the 1 kb sequence upstream of the translation initiation codon in ZmLTP genes. [file 12870_2014_281_MOESM13_ESM.pdf]

Table S9. Analysis of *cis*-elements in the 1 kb sequence upstream of the translation initiation codon in ZmLTP genes.

***cis*-lement**	**Function**	**ZmLTP1.1**	**ZmLTP1.2**	**ZmLTP1.3**	**ZmLTP1.4**	**ZmLTP1.5**	**ZmLTP1.6**	**ZmLTP1.7**	**ZmLTP1.8**	**ZmLTP2.1**
RY-element	*cis*-acting regulatory element involved in seed-specific regulation									
O2-site[18]	*cis*-acting regulatory element involved in zein metabolism regulation				1		1			
CAT-box	*cis*-acting regulatory element related to meristem expression							1	1	1
CCGTCC-box	*cis*-acting regulatory element related to meristem specific activation				4		1			1
NON-box	*cis*-acting regulatory element related to meristem specific activation									
OCT	*cis*-acting regulatory element related to meristem specific activation									
Skn-1_motif	*cis*-acting regulatory element required for endosperm expression	1	1	2	1	2	1	1	3	2
AACA_motif	involved in endosperm-specific negative expression									
GCN4_motif[25]	*cis*-regulatory element involved in endosperm expression					1	1			1
circadian[9]	*cis*-acting regulatory element involved in circadian control		1	2					2	
MSA-like	*cis*-acting element involved in cell cycle regulation						1		1	
as-2-box[8]	involved in shoot-specific expression and light responsiveness							1		
ACE[15]	*cis*-acting element involved in light responsiveness								1	
C-box	*cis*-acting regulatory element involved in light responsiveness				1					
G-Box[10]	*cis*-acting regulatory element involved in light responsiveness				1		3	6	2	
G-box	*cis*-acting regulatory element involved in light responsiveness	1	1		6	3	4	10	5	2
ATCC-motif	part of a conserved DNA module involved in light responsiveness									
ATCT-motif	part of a conserved DNA module involved in light responsiveness						1			
Box 4[12]	part of a conserved DNA module involved in light responsiveness					1	1	2		2
AE-box[19]	part of a module for light response	1								
AT1-motif	part of a light responsive module									
Box II	part of a light responsive element									
CATT-motif	part of a light responsive element	1						2	1	
chs-CMA2a	part of a light responsive element									
chs-Unit 1 m1	part of a light responsive element									
GAG-motif [11]	part of a light responsive element				1		2	2		1
GA-motif[26]	part of a light responsive element			1			1			
Gap-box	part of a light responsive element									
GATA-motif[1]	part of a light responsive element	1	1					1	1	1
GATT-motif	part of a light responsive element					1				1
GTGGC-motif	part of a light responsive element									
I-box[4]	part of a light responsive element	1				1	1	1	2	2
LAMP-element	part of a light responsive element									
L-box	part of a light responsive element		1					1		
LS7	part of a light responsive element									
rbcS-CMA7a	part of a light responsive element									
TCCC-motif[20]	part of a light responsive element					1				
TCT-motif[27]	part of a light responsive element						1			
TGG-motif	part of a light responsive element									
3-AF1	light responsive element									
4cl-CMA2a	light responsive element									
AAAC-motif	light responsive element									
Box I [23]	light responsive element	1								
GT1-motif[2]	light responsive element		3				1	2	1	
MNF1	light responsive element		1	1		2				
Sp1	light responsive element	4	1		4		2	2	5	
TGA-element[21]	auxin-responsive element								1	
AuxRR-core	*cis*-acting regulatory element involved in auxin responsiveness	1	1	1					1	
CGTCA-motif	*cis*-acting regulatory element involved in the MeJA-responsiveness		1	3	3			2	1	2
TGACG-motif	*cis*-acting regulatory element involved in the MeJA-responsiveness		1	3	3			2	1	2
TCA-element[24]	*cis*-acting element involved in salicylic acid responsiveness	1		1	1			1		
SARE	*cis*-acting element involved in salicylic acid responsiveness									
ABRE[6]	*cis*-acting element involved in the abscisic acid responsiveness	2				1		1	2	
motif IIb	abscisic acid responsive element								1	
CE3	*cis*-acting element involved in ABA and VP1 responsiveness									
TATC-box	*cis*-acting element involved in gibberellin-responsiveness					1				45
GARE-motif[3]	gibberellin-responsive element			1		1				2
P-box[22]	gibberellin-responsive element				1					
ERE[28]	ethylene-responsive element									
Box-W1[16]	fungal elicitor responsive element									1
EIRE	elicitor-responsive element									
WUN-motif	wound-responsive element									
TC-rich repeats[17]	*cis*-acting element involved in defense and stress responsiveness	2	1							
HSE[24]	*cis*-acting element involved in heat stress responsiveness					1	1			
LTR[7]	*cis*-acting element involved in low-temperature responsiveness				1	1				1
C-repeat/DRE	regulatory element involved in cold- and dehydration-responsiveness				1					
ARE[13]	*cis*-acting regulatory element essential for the anaerobic induction	1								1
GC-motif[13]	enhancer-like element involved in anoxic specific inducibility	1			2			1	1	
MBS[5]	MYB binding site involved in drought-inducibility		2		2	1	1	1		3
MBSII	MYB binding site involved in flavonoid biosynthetic genes regulation	1								
MBSII	MYB binding site involved in flavonoid biosynthetic genes regulation	1								
MRE	MYB binding site involved in light responsiveness			1						1
CCAAT-box	MYBHv1 binding site		1	1	1		1			
Box III	protein binding site	1								
AT-rich element	Binding site of AT-rich DNA binding protein (ATBP-1)									
A-box	*cis*-acting regulatory element				2	1	5	9		
OBP-1 site	*cis*-acting regulatory element									
ATGCAAAT motif	*cis*-acting regulatory element associated to the TGAGTCA motif									
TA-rich region	enhancer					33				
5UTR Py-rich stretch [14]	*cis*-acting element conferring high transcription levels				4		2	1	2	1
CAAT-box	common *cis*-acting element in promoter and enhancer regions	8	15	6	8	12	6	14	7	15
TATA-box	core promoter element around -30 of transcription start	18	19	22	5	109	30	30	10	45

ZmLTP2.2	ZmLTP2.3	ZmLTP2.4	ZmLTP2.5	ZmLTP2.6	ZmLTP2.7	ZmLTP2.8	ZmLTP2.9	ZmLTPc1	ZmLTPc2	ZmLTPd1	ZmLTPd2	ZmLTPd3	ZmLTPd4	ZmLTPd5	ZmLTPd6	ZmLTPd7	ZmLTPd8	ZmLTPd9
	1		1											1	1	1		
		1	1		1	2			1		1			2		1		
1					2	2				2		1		2		3		
1		1				1		4		2			1	2		3		
																1		
	1	1	1	1		2	3		1		3		1	1	1	3	2	1
															1			
1		1		1	2	1	1			1	1				1			
			2			1			3	2		4			1	2		
							1											
1	1		1	1	2		1				1						2	2
1		2			1	3	2	1	2		1	1	1	2	1	1	6	1
6		5		2	2	7	3	2	2		1	2	1	4	2	3	10	1
						1												
	1		1								1			1	1			
	2		1	1						1	3				1			
1											3				1	1	1	1
											1							
								1										
		5	1											1		1		
													1					
	1																	
1					1	1		2	1				1	1	1			1
								1							2			
							1											
			1		1							1						
1	1				2	1								1				2
			1															
			1															1
	1		1								1				1			
															1			
					1													1
2	1		1	1					1	2				1	1	2		1
			2		1			1			2			2				2
		1											1					
	1	2	1	2	2	4	4	4	1	2		1	1	4	1			
	1	1	1				1			1	1		1	2		1	1	1
1				2						2								3
2			1			3	3		1		2	2	1			1	1	
2			1		3	3	3		1		2	2	1			3	1	
1	2	1		1	1	1	1		1	3	1		1			1		1
1	5	1	2		1	1				1		1		2	1			1
					1	1	1		1			1						
1		1	1						1				1					
															1			
			1				2										3	
		1	1		1													
				1						1				1		1		1
						1			1			1	1		1	1		
	1						1				2				1	2		
1				1		1								1	2		1	1
				1				1				2	1					
	1						1	2									1	1
		1					2	1	2	1			2			3		
		3	1	1	1			3	2	1	2	3					1	
1							1		1						1			
		1			1	1												1
															1			
		2		2	1	2	3	1	2					2	1	2	6	1
											1							
												1						
1		1				1		4		2			1	2		1		
10	10	6	11	16	12	11	5	11	18	15	12	12	4	13	20	8	7	19
43	93	13	27	28	13	6	22	13	23	32	39	16	8	19	74	35	7	16

ZmLTPd10	ZmLTPd11	ZmLTPd12	ZmLTPd13	ZmLTPd14	ZmLTPd15	ZmLTPd16	ZmLTPg1	ZmLTPg2	ZmLTPg3	ZmLTPg4	ZmLTPg5	ZmLTPg6	ZmLTPg7	ZmLTPg8	ZmLTPg9	ZmLTPg10	ZmLTPg11	ZmLTPg12
															1	1		1
1				1	2	1	1	1						2	1			
	1			2			2							1				
	3						2			2			1		1		1	
																		1
3	2	2		3	2	3	2	1	2	2	1		2	1	1	1		1
1					1	1	2	1		2	2		1					
				1	3	2		1	1					1				
								1	1									
											1							
1							1				1	1				1		
1			1	2		1	2	2		2	3	2		1	3	1		
1	1	2	2	2	2	3	4	5		6	4	4	3	3	7	3	3	2
1	2	1		1								1				1		
3			1	1	1	3		2					2	1				
3		1		1											1		1	
								1										
									1									
		1								1					1			
	1						1		1									
												1	1					
	1	3	1			1	1	1			1		1					
										1	1	1						
				1														1
		1				1						1				1		
							3											
		2		1														
																1		
			1				1							1				
					1													
				1					1						1			
1	1	1		1								1				1		
												1		1				
		1				1			2		1	1	1				1	
2		1			1				2	1		1				2		
							1			1							1	
		1	3	2	1	1	2		1	6				1	3		1	1
1	2		1								1							1
											1						2	
2	5	1	6		1	1	3	3		3		1	5		2	2		
2	5	1	6		1	1	3	3		3		1	5		2	2		
								1				1						
1		1	1	1	2	2	1	1						1			1	
							1											
							1											
	1				1	1			2		1			2	1	2	1	
														1				
		1									1	1				1		
			1									1	1	2				3
														1				
									1									
2				1							2	2					2	1
		1			1	1						3	2			1	1	
	1							2					2					1
																		1
												1						
		0	1							2			1					2
2		1	2	3	2		1		1	2		2	1	1	2			5
	1		1															
	1							3	1	2			1			2		2
		1		1	1	1												
											1	1						
		2		3			2	2		2	2	2	1	1	7	1	1	
1																		
	3						2			2			1		1		1	
12	11	10	8	21	22	23	13	19	16	10	28	14	19	15	8	14	17	8
39	18	35	10	47	26	26	4	28	32	1	26	40	13	22	9	47	13	15

ZmLTPg13	ZmLTPg14	ZmLTPg15	ZmLTPg16	ZmLTPg17	ZmLTPg18	ZmLTPg19	ZmLTPg20	ZmLTPg21	ZmLTPg22	ZmLTPg23	ZmLTPg24	ZmLTPg25	ZmLTPg26	ZmLTPx1	ZmLTPx2
						1		2		1		1			
	1		2	1	1	1	1		2	1		1		2	3
		2	2						3	1	1	2	1	2	
										1					
										1					
	3	1		2	1		3	2	2	1		2	2		3
				1			1	2	1		2				
									1					1	1
										1					
1	1	4		1				2		1					
				1							1				
2	2		3	3	3	2		1	1				2		
	5	1	6	10	6	5		2	5	2		1	3	1	2
			1	1											
		1	1			1			4	1					
					1		1						1		
			1	1											
1						2	1		1	1				1	1
											1				
		1		1											
1								1	1						
5			1							2				1	
						1									
	1		1												
				1						1	1				
	2			1				1		1	1		1	1	
						1									
															1
		1													
			1	1											
				1											
														1	
					1										
	3	2				2		1		1		1			
1								1							
		1		2	1						1		1		
	2			1	5	1			19	2	5	1		2	15
			2	1		3		1		1	1				
1		1			2	1	1	1		2					
2	2	1	1	2		1	1	4	2	2	3	2			7
2	2	1	1	2		1	1	4	2	2	3	2			7
			1	1		2	1	1		1		1	2	1	
						1									
2	1		1		1	1	1	2		1			1		
											1				
1															
												1		1	
		1	1									2			1
1										1				1	
1		2	1	1		2					2		1		
						1							1		
2	1					2				1		1	1		
1	2			2				1	1						
	1					1					1				
		1								1	1				
			1	1	3					1					
1		2	2	1	1					1	1		1	2	2
1			1		2				3	1					1
							1								
	1														1
5	5	1	3	9	5			2	2			1	1		1
									1						
						1				1					
		2	2						3	1	1	2	2	2	
10	14	21	21	5	8	17	21	18	9	9	15	10	11	15	21
28	24	28	25	28	24	27	25	43	26	18	9	4	36	26	4

References cited in this table:

1. Gilmartin PM, Sarokin L, Memelink J, Chua NH: Molecular light switches for plant genes. Plant Cell 1990, 2:369-378.
2. Villain P, Mache R, Zhou DX: The mechanism of GT element-mediated cell type-specific transcriptional control. J Biol Chem 1996, 271:32593-32598.
3. Ogawa M, Hanada A, Yamauchi Y, Kuwahara A, Kamiya Y, Yamaguchi S: Gibberellin biosynthesis and response during Arabidopsis seed germination. Plant Cell 2003, 15:1591-1604.
4. Terzaghi WB, Cashmore AR: Light-regulated transcription. Annu Rev Plant Physiol Plant Mol Biol 1995, 46:445-474.
5. Yamaguchi-Shinozaki K, Shinozaki K: Arabidopsis DNA encoding two desiccation-responsive rd29 genes. Plant Physiol 1993, 101:1119-1120.
6. Simpson SD, Nakashima K, Narusaka Y, Seki M, Shinozaki K, Yamaguchi-Shinozaki K: Two different novel cis-acting elements of erd1, a clpA homologous Arabidopsis gene function in induction by dehydration stress and dark-induced senescence. Plant J 2003, 33:259-270.
7. Baker SS, Wilhelm KS, Thomashow MF: The 5’-region of Arabidopsis thaliana core15a has cis-acting elements that confer cold-, drought- and ABA-regulated gene expression. Plant Mol Biol 1994, 24:701-713.

8. Diaz-De-Leon F, Klotz KL, Lagrimini M: Nucleotide sequence of the tobacco (Nicotiana tabacum) anionic peroxidase gene. Plant Physiol 1993, 101:1117-1118.

9. Pichersky E, Bernatzky R, Tanksley SD, Breidenbach RB, Kausch AP, Cashmore AR: Molecular characterization and genetic mapping of two clusters of genes encoding chlorophyll a/b-biding proteins in Lycopersicon esculentum (tomato). Gene 1985, 40:247-258.

10. Sommer H, Saedler H: Structure of the chalcone synthase gene of Antirrhinum majus. Mol Gen Genet 1986, 202:429-434.

11. Werneke JM, Chatfield JM, Ogren WL: Alternative mRNA splicing generates the two ribulosebisphosphate carboxylase/oxygenase activase polypeptides in spinach and Arabidopsis. Plant Cell 1989, 1:815-825.

12. Lois R, Dietrich A, Hahlbrock K, Schulz W: A phenylalanine ammonia-lyase gene from parsley: structure, regulation and identification of elicitor and light responsive cis-acting elements. EMBO J 1989, 8:1641-1648.

13. Manjunath S, Sachs MM: Molecular characterization and promoter analysis of the maize cytosolic glyceraldehyde 3-phosphate dehydrogenase gene family and its expression during anoxia. Plant Mol Biol 1997, 33:97-112.

14. Daraselia ND, Tarchevskaya S, Narita JO: The promoter for tomato 3-hydroxy-3-methylglutaryl coenzyme A reductase gene 2 has unusual regulatory elements that direct high-level expression. Plant Physiol 1996, 112:727-733.

15. Feldbrugge M, Sprenger M, Dinkelbach M, Yazaki K, Harter K, Weisshaar B: Functional analysis of a light-responsive plant bZIP transcriptional regulator. Plant Cell 1994, 6:1607-1621.

16. Rushton PJ, Torres JT, Parniske M, Wernert P, Hahlbrock K, Somssich I.E: Interaction of elicitor-induced DNA-binding proteins with elicitor response elements in the promoters of parsley PR1 genes. EMBO J 1996, 15:5690-5700.

17. Diaz-De-Leon F, Klotz KL, Lagrimini M: Nucleotide sequence of the tobacco (Nicotiana tabacum) anionic peroxidase gene. Plant Physiol 1993, 101:1117-1118.

18. Hartings H, Lazzaroni N, Marsan PA, Aragay A, Thompson R, Salamini F, Di Fonzo N, Palau J, Motto M: The b-32 protein from maize endosperm: characterization of genomic sequences encoding two alternative central domains. Plant Mol Biol 1990, 14:1031-1040.

19. Conley TR, Park SC, Kwon HB, Peng HS, Shih MC: Characterization of cis-acting elements in light regulation of the nuclear gene encoding the A subunit of chloroplast isozymes of glyceraldehyde-3-phosphate dehydrogenase from Arabidopsis thaliana. Mol Cell Biol 1994, 14:2525-2533.

20. Bichler J, Herrmann RG: Analysis of the promotors of the single-copy genes for plastocyanin and subunit delta of the chloroplast ATP synthase from spinach. Eur J Biochem 1990, 190:415-426.

21. Pastuglia M, Roby D, Dumas C, Cock JM: Rapid induction by wounding and bacterial infection of an S gene family receptor-like kinase in Brassica oleracea. Plant Cell 1997, 9:1-13.

22. Kim JK, Cao J, Wu R: Regulation and interaction of multiple protein factors with the proximal promoter regions of a rice high pI alpha-amylase gene. Mol Gen Genet 1992, 232:383-393.

23. Kuhlemeier C, Cuozzo M, Green PJ, Goyvaerts E, Ward K, Chua NH: Localization and conditional redundancy of regulatory elements in rbcS-3A, a pea gene encoding the small subunit of ribulose-bisphosphate carboxylase. Proc Natl Acad Sci USA 1988, 85:4662-4666.

24. Pastuglia M, Roby D, Dumas C, Cock JM: Rapid induction by wounding and bacterial infection of an S gene family receptor-like kinase in Brassica oleracea. Plant Cell 1997, 9:1-13

25. Kim SY, Wu R: Multiple protein factors bind to a rice glutelin promoter region. Nucleic Acids Res 1990, 18:6845-6852..

26. Grandbastien MA, Berry-Lowe SL, Shirley BW, Meagher RB: Two soybean ribulose-1,5-bisphosphate carboxylase small subunit genes share extensive homology even in distant flanking sequences. Plant Mol Biol 1986, 7:451-465.

27. Kwon HB, Park SC, Peng HP, Goodman HM, Dewdney J, Shih MC: Identification of a light-responsive region of the nuclear gene encoding the B subunit of chloroplast glyceraldehyde 3-phosphate dehydrogenase from Arabidopsis thaliana. Plant Physiol 1994, 105:357-367.

28. Itzhaki H, Woodson WR: Characterization of an ethylene-responsive glutathione S-transferase gene cluster in carnation. Plant Mol Biol 1993, 22:43-58.
