# Supplementary material for: Non-specific lipid transfer proteins in maize
Source: BMC Plant Biol. 2014 Oct 28;14:281. doi: 10.1186/s12870-014-0281-8 (PMC4226865; doi:10.1186/s12870-014-0281-8)
Supplement: Additional file 14: Table S10. — Average log2 signal intensity values and CV values of 75 detected ZmLTP transcripts in sixty distinct tissues representing eleven major organ systems of inbred line B73 ordered by three clusters. [file 12870_2014_281_MOESM14_ESM.pdf]

Table S10. Average log2 signal intensity values and CV values of 75 detected ZmLTP transcripts in sixty distinct tissues representing eleven major organ systems of inbred line B73 ordered by three clusters.

Cluster	Probe ID	Name	Germinating Seed	Root			Whole Seedling		Stem and SAM		
			24H_Germinating Seed	6DAS_GH_Primary Root	VE_Primary Root	V1_GH_Primary Root	6DAS_GH_Coleoptile	VE_Whole Seedling	V1_Stem and SAM	V3_Stem and SAM	V4_Stem and SAM
	GRMZM2G027378_T01	E2enzyme	14.99108344	14.96681372	15.08497063	15.13858015	14.64734925	14.68300126	14.91667599	14.89353998	14.76298105
	GRMZM2G083725_T01	ZmLTPg4	13.87081295	9.798044608	9.566718994	9.279310397	12.54429042	12.66984775	11.66176408	11.23747209	11.00543328
	GRMZM2G101958_T01	ZmLTP1.6	15.43959056	5.944157934	5.86597871	5.708280789	14.77260169	13.97230106	11.98962401	12.1247562	11.8650346
	GRMZM2G137329_T01	ZmLTP2.1	9.781391871	6.10518166	6.04857669	5.614069729	11.70236284	11.72127898	8.27527778	9.169359349	9.801450538
	GRMZM2G107839_T02	ZmLTP1.3.2	7.930266572	4.960581101	7.751873576	6.385911515	10.39280923	13.25931411	11.11643015	11.19503787	11.56415078
	GRMZM2G107839_T01	ZmLTP1.3.1	8.260831797	5.422447829	8.148904024	6.857657216	11.26990716	13.91562197	11.72152189	11.89933689	12.25289454
	GRMZM2G010868_T01	ZmLTP1.2.1	12.83178981	5.120302147	4.953554627	5.310347513	13.52072236	13.44654956	11.87526323	11.24417945	11.33791002
	GRMZM2G010868_T02	ZmLTP1.2.2	13.36298633	5.573491295	5.299871143	5.628925855	13.57608914	13.40283343	11.49475772	10.91574486	10.99955154
	GRMZM2G170969_T01	ZmLTPd13	7.83956002	11.72300744	12.17967237	10.76136752	7.348852847	7.63068494	10.5212676	10.33023919	10.84936804
	GRMZM2G126397_T01	ZmLTP1.1	8.465328269	5.585514215	5.78419275	5.621128317	13.25788126	12.82102273	12.99856183	12.48069183	11.88653622
I	GRMZM2G136364_T02	ZmLTPd3.1	8.55897357	9.594155846	10.68928615	9.106544672	9.542046517	9.649223042	10.6516256	10.95219133	14.3996985
	GRMZM2G136364_T01	ZmLTPd3.2	8.55897357	9.594155846	10.68928615	9.106544672	9.542046517	9.649223042	10.6516256	10.95219133	14.3996985
	GRMZM2G164440_T01	ZmLTPd5	9.275555412	11.16326919	12.70851287	11.54977129	9.804099033	10.99006754	11.26394203	11.16719111	11.53672245
	GRMZM2G141858_T02	ZmLTPg17.2	9.183614447	12.57051706	11.33685615	11.75255043	11.34452014	11.25550541	11.74505377	11.31408924	11.78429808
	GRMZM2G141858_T01	ZmLTPg17.1	9.863915432	13.06191773	11.8169141	12.24608882	11.74752236	11.73746803	12.10576216	11.81137787	12.23999155
	GRMZM2G089400_T01	ZmLTPg19	11.08186433	10.4815189	11.64035122	10.80695422	10.69425675	10.61462221	10.75967658	10.75008117	10.86104227
	GRMZM2G078876_T01	ZmLTPg3	11.39754703	11.25258837	10.75483925	10.46094757	11.90724091	11.35134605	11.3210038	10.93393667	10.40344711
	GRMZM5G898755_T02	ZmLTP1.7.1	5.922265114	9.35679242	6.406865643	7.292075182	13.07291472	12.19344665	12.56366231	12.84180539	13.20454547
	GRMZM5G898755_T01	ZmLTP1.7.2	6.031675328	9.644585551	6.672254382	7.532602634	13.61430747	12.68239662	12.9602683	13.23674861	13.69946677
	GRMZM5G850455_T02	ZmLTPg1.2	10.84871682	8.502349853	7.157336315	6.691846906	12.5643128	12.23680715	13.34330358	13.35090871	13.22172759
	GRMZM5G850455_T01	ZmLTPg1.1	10.45803785	8.432620369	7.128376993	6.662665768	12.53459291	12.18733932	13.22578827	13.31221039	13.18817246
	GRMZM5G850455_T03	ZmLTPg1.3	10.45803785	8.432620369	7.128376993	6.662665768	12.53459291	12.18733932	13.22578827	13.31221039	13.18817246
	GRMZM2G091054_T01	ZmLTPd14	7.546653517	4.711569209	4.757126488	4.766745108	4.671617883	4.856082958	4.634610935	4.767328062	4.737055439
	GRMZM2G406552_T01	ZmLTP2.3	6.211689595	6.448964505	5.46784283	5.698830261	5.614353809	6.035973217	5.523002235	5.153593929	5.35924308
	GRMZM2G087413_T01	ZmLTPd6	6.36506539	5.294239939	5.147739929	5.068861703	5.20272888	5.297904354	5.34691446	5.205323888	5.286879765
	GRMZM2G081464_T01	ZmLTP2.2	12.33738346	4.551674596	4.730742698	4.695722129	4.99370791	5.254608636	4.892007412	4.963768331	4.747189336
II	GRMZM2G063375_T01	ZmLTPd16	5.311377292	4.782482261	4.723191578	4.823482248	4.790297653	4.827412215	4.94069201	4.755430377	4.646265421
	GRMZM2G155555_T01	ZmLTPd15	5.274446575	4.751429433	4.699864146	4.812150202	4.755888265	4.936002419	4.909747552	4.762361727	4.697828079
	GRMZM2G387360_T01	ZmLTP2.4	4.984559403	5.469276357	5.489543749	5.257845594	5.524424717	8.272927672	8.090119231	6.640191783	5.860390759
	GRMZM2G166484_T01	ZmLTPx2	8.00434085	7.605477457	7.463015478	7.619487551	7.091376343	8.378530977	7.052680961	7.92469698	7.639205208
	GRMZM2G071575_T01	ZmLTPg14	12.31359798	6.355488282	6.147973867	5.944248849	8.171251756	8.638227938	7.811545734	7.325482095	7.348346796
	GRMZM2G403007_T01	ZmLTP2.6	6.725901712	14.4260496	13.16111882	13.53123194	11.78822413	8.490444606	10.63449006	10.84334673	11.94154429
	GRMZM2G004909_T01	ZmLTP2.7	6.134896778	13.65041712	12.71530221	12.96139907	9.95799559	9.00642108	9.565376503	9.233501145	10.8395306
	GRMZM2G031102_T01	ZmLTPd1	6.042274091	13.80285917	14.52636537	14.12600422	9.233038111	11.41613854	10.47228365	9.150929803	11.05294268
	GRMZM2G099867_T01	ZmLTPd4	6.042274091	13.80285917	14.52636537	14.12600422	9.233038111	11.41613854	10.47228365	9.150929803	11.05294268
	GRMZM2G168833_T01	ZmLTPg9	6.472667529	6.253107803	6.300233929	6.1326723	8.675208247	9.899354262	6.645632074	6.489101947	6.595204149
	GRMZM2G005991_T01	ZmLTPg7	5.926548638	7.51703008	11.33375848	12.35132163	6.621747027	11.82066366	7.118810064	7.336635911	6.890032281
	GRMZM2G006047_T02	ZmLTPg6	6.710132523	9.190946032	12.0556046	12.85110679	7.931172207	12.03817705	9.038675772	8.910163487	7.445716305
	GRMZM2G170044_T01	ZmLTPg22	11.75291457	9.843314368	10.3980977	8.940283468	8.044709355	10.1112996	9.078827159	9.567440717	10.2298226
	GRMZM2G004466_T01	ZmLTPg25	6.271925412	9.147292831	9.865381436	8.275933343	8.251338097	9.758383976	9.49743278	10.35568197	10.97779353
	GRMZM2G065557_T01	ZmLTPd8	6.924854401	10.84611322	10.79655138	9.393593067	8.869856983	9.334800181	8.606041287	9.91331258	11.33182535
	GRMZM2G320373_T01	ZmLTP2.8	11.45190881	10.17273512	10.33634988	8.624651011	8.821365324	8.035352868	7.959556661	8.38290461	10.37181735
	GRMZM2G116167_T01	ZmLTPg18	6.548395756	9.690543281	8.633749113	8.763806117	10.59921311	8.711253352	8.377524889	8.733767386	8.248962733
	GRMZM2G097137_T01	ZmLTPg5	7.784534579	8.091670568	9.597044029	10.70686402	7.873424248	8.148832988	7.825260982	8.03332879	7.794217118
	GRMZM2G140646_T01	ZmLTPg26	7.593281673	8.024727445	7.878006909	8.093285737	8.099376552	8.206925531	7.515060076	8.11234466	7.876561108
	GRMZM2G046750_T01	ZmLTPg21	5.165703919	6.00009625	8.91596149	10.05785895	5.383756125	9.978171506	6.275136072	6.353476447	5.614176371
	GRMZM2G130454_T03	ZmLTPg16	5.095868854	5.683813072	8.783022239	9.908405032	5.458236712	8.913644004	6.1672417	5.832798339	5.458973505
	GRMZM2G176347_T01	ZmLTPg13	5.286333506	5.875321566	7.901471021	8.718545027	5.743552848	7.505803007	5.604549793	5.632455561	5.706802186
	GRMZM2G096234_T01	ZmLTP1.4	10.55290918	7.359112816	6.858275729	6.326417745	6.451103924	6.299796838	6.213997137	6.118161997	6.145963427
	GRMZM2G094632_T01	ZmLTPd9.2	6.592360492	6.761987586	6.985999198	7.035433788	5.927611216	7.69560213	6.397389264	6.633349947	7.334615258
	GRMZM2G094632_T02	ZmLTPd9.1	6.828023813	6.906259569	7.397146127	7.215429253	6.106037324	8.206240611	6.541963535	7.046774959	7.744319714
	AC225127.3_FGT003	ZmLTPc1	6.888084351	5.77997372	5.659487855	5.68465593	5.979955421	5.501663841	5.963336406	5.493577386	5.499831208
	GRMZM2G073377_T01	ZmLTPc2.1	5.162498604	5.298899966	5.11150631	5.381963947	5.22091915	5.484212649	5.007371489	5.075899027	5.174243621
III	GRMZM2G073377_T04	ZmLTPc2.2	5.277676516	5.782212	5.528788092	5.466184848	5.710435065	6.090537039	5.464521187	5.5615939	5.671433542
	GRMZM2G025026_T02	ZmLTP1.5.2	5.283649373	5.46264739	5.653375731	5.565853048	5.135099522	5.120616946	5.482822845	5.519704624	6.167380399
	GRMZM2G025026_T01	ZmLTP1.5.1	5.653628217	5.508313728	5.653375731	5.552455196	5.140730212	5.173658459	5.682992263	5.441734433	6.167380399
	GRMZM2G393150_T01	ZmLTP2.9	5.803418447	5.170244143	5.055817482	5.246089265	5.138632806	5.188222401	5.481397134	5.326263156	5.752435688
	GRMZM2G036063_T03	ZmLTPx1.3	5.454025693	5.528377847	5.380563059	5.255061282	5.355678461	5.276782447	5.097496938	5.24682949	5.310462853
	GRMZM2G036063_T01	ZmLTPx1.1	5.251793061	5.403031432	5.283659177	5.299423991	5.51036061	5.387491007	5.166595576	5.176206785	5.542989375
	GRMZM2G036063_T02	ZmLTPx1.2	5.251793061	5.403031432	5.283659177	5.299423991	5.51036061	5.387491007	5.166595576	5.176206785	5.542989375
	GRMZM2G439268_T01	ZmLTP1.8	10.36871047	5.433531275	5.315425256	5.533900945	5.397205118	5.370978262	4.778752114	4.933716461	5.204089133
	GRMZM2G151021_T01	ZmLTPg11	5.216806318	5.243268935	5.295664933	5.119975515	5.252780826	5.775937271	5.906597873	5.177849977	5.452794366
	GRMZM2G039383_T01	ZmLTP2.5	7.524092393	5.988345827	6.206465945	6.044498626	10.87973886	6.029012445	6.135726247	6.041820087	6.156668703
	GRMZM2G174680_T01	ZmLTPg2	5.816646521	5.852296583	6.070719576	5.939900477	6.279158714	6.262293037	6.245314917	6.465691846	6.628111178
	GRMZM2G396418_T01	ZmLTPd7	5.923695546	7.439358836	10.07395092	8.043142827	6.094698177	6.0348401	6.70214602	7.111344214	6.500530452
	GRMZM2G089288_T03	ZmLTPg20.1	5.179618428	5.942063858	5.450877898	5.538940181	5.583682794	5.490266022	5.260680872	5.47774906	5.608765687
	GRMZM2G089288_T01	ZmLTPg20.2	5.291410193	6.216755863	5.662245691	5.458080128	5.682952977	5.647734834	5.235892617	5.671959658	6.136027935
	GRMZM2G414620_T01	ZmLTPg10	5.446991747	5.008098527	5.120388063	4.920508756	5.356686411	6.429019856	5.969936408	5.147886635	5.007058411
	GRMZM2G000221_T01	ZmLTPd11	4.766922227	5.293302262	7.307066798	5.575794342	5.623192963	5.69528277	5.558890141	5.871860569	5.435353873
	GRMZM2G071771_T01	ZmLTPd2	4.690505122	4.753813633	5.660813561	5.037708171	5.099809559	5.409730164	5.466363498	6.023970862	5.922730412
	AC233926.1_FGT002	ZmLTPd10	4.615710639	4.786765655	5.505312461	4.845961905	5.268871033	5.372355224	5.426734206	5.904212969	5.84690954
	GRMZM2G471051_T01	ZmLTPd12	5.006227778	7.203756337	7.205348054	6.200014857	4.889491418	4.791634905	6.044365157	5.536500486	5.743264844
	AC194203.3_FGT003	ZmLTPg12	5.381263266	6.162177622	6.554506579	6.556226374	5.856567977	5.60914129	5.672161801	6.090225789	6.146539116
	GRMZM2G171597_T01	ZmLTPg23	4.944060287	5.294342281	5.26070173	5.112768034	5.315645244	5.432640648	5.304674254	5.433049634	5.603765316
	GRMZM2G065640_T01	ZmLTPg8.1	5.018551884	5.174477045	5.482030492	5.728622414	5.343865422	5.874804726	5.008186652	5.292017489	5.05327472
	GRMZM2G065640_T02	ZmLTPg8.2	4.901736948	5.198177601	5.311842508	5.719336487	5.335704632	5.975137089	4.966231408	5.304590333	5.181397806

	**Internode**			**Cob**		**Leaves**				
V5_Shoot Tip	V5_First Internode	V7_First Internode	V9_Fourth Internode	V18_Immature Cob	R1_Pre-pollination Cob	V1_Pooled Leaves	V3_First Leaf and Sheath	V3_Topmost Leaf	V5_Base of stage-2 Leaf	V7_Base of stage-2 Leaf
14.95020591	14.89897196	14.91088606	15.04541993	14.81239968	15.00153645	14.71295858	15.03668792	14.62578307	14.67739792	14.96059379
10.48052697	10.28007168	10.1151912	8.836060239	11.74875268	13.04109927	12.65255558	12.36844789	12.92337686	12.56785184	12.77825897
11.03278346	11.71327151	11.0524337	9.006933633	7.655210343	10.42019268	14.42517946	14.81542795	14.40973837	13.59933077	13.19408424
7.130579871	8.792656321	8.700491951	6.071929818	5.769905034	7.457539422	13.65724689	13.92123685	13.11526829	7.911805615	8.450275743
9.520982661	10.61646727	11.40746325	5.150949396	5.23497808	6.145949042	13.50689077	12.35579622	13.72178919	12.56161769	10.78143201
10.2378934	11.47776862	12.10474889	5.454935074	5.521947505	6.489718001	14.0458245	13.16810619	14.22777303	13.25224884	11.46380992
10.79465057	10.48193261	10.50397454	6.986419886	6.269061292	11.61996859	13.37141649	12.90597293	13.62763889	13.48794969	13.09027857
10.2687132	10.43065173	10.49393478	7.548207696	6.398919755	11.3718515	13.45311547	12.99677114	13.59918424	13.63774325	13.18508001
10.94057629	11.70765276	11.37493071	9.415123152	9.486233756	12.4489527	5.91967844	5.844246193	6.068672619	7.994276367	7.274500144
12.07188453	10.07509537	8.246652692	10.83745442	12.45991551	13.80326823	7.161891387	6.715038033	10.27268263	12.93734133	13.60531302
12.19085846	13.31584142	15.08267137	13.44075495	8.993323844	11.25885523	8.879254467	7.869386749	9.898978483	10.73426128	12.67139392
12.19085846	13.31584142	15.08267137	13.44075495	8.993323844	11.25885523	8.879254467	7.869386749	9.898978483	10.73426128	12.67139392
11.42155473	11.79181895	12.41704	12.19282838	11.11218503	11.23109305	11.44935747	9.15215861	10.81143068	11.93924078	11.89859715
11.85311192	11.15796888	11.89329658	12.15153007	11.20337445	11.64681413	11.28841134	10.69828841	11.25471985	10.8589648	11.30182754
12.28750011	11.59609885	12.34354059	12.57979732	11.73426785	12.1239076	11.69188504	10.95559518	11.70438844	11.26006841	11.72400438
11.05192881	11.36282798	11.53991697	11.20531801	9.17523721	10.52659417	10.35263552	11.85817469	10.26834441	10.01745309	10.19582093
11.13145825	11.26918055	11.40136195	11.89382604	11.15100229	12.69101951	10.8880534	9.781712723	11.28433569	12.71637928	12.89550714
13.57587849	13.4404095	13.6623195	13.50367008	11.64875825	13.7445634	6.424127023	6.974334019	10.35645012	14.20775913	14.23142664
14.02891706	13.87379553	14.14303967	14.04701297	11.94954179	14.1010431	6.199333066	6.854699382	10.67463744	14.61343432	14.64247716
13.64251999	12.91446719	13.16087093	12.73428929	13.23669274	13.91211896	6.610086052	8.229420646	9.621090103	13.46953517	13.9828609
13.61302192	12.89857199	13.14081774	12.70393553	13.22638707	13.88239907	6.718888869	8.194145134	9.49213269	13.43841779	14.06123125
13.61302192	12.89857199	13.14081774	12.70393553	13.22638707	13.88239907	6.718888869	8.194145134	9.49213269	13.43841779	14.06123125
4.797696856	4.775335135	4.702723746	4.773306485	5.02047952	4.886266696	5.01192913	4.831634901	5.113073322	4.736243409	4.738897878
5.158114317	5.225662272	5.168299251	5.292529617	5.341899576	5.262169663	5.330350594	5.246578262	5.311106519	5.188291667	5.233150973
5.236543287	5.374672973	5.091698518	5.293560189	5.873982073	5.399780862	5.243669721	5.043074295	5.37476538	5.12275425	5.111526656
4.997114915	4.745030078	4.700633144	4.892736209	5.019592243	4.684129397	5.452693087	5.407119679	5.153783155	4.86564864	4.948041999
4.947797273	4.587165699	4.70961427	4.564402364	4.73756265	4.559742545	4.955493323	5.053042908	5.06193027	4.636646382	4.963134297
4.858322261	4.627827836	4.737038949	4.723633386	4.746058311	4.570433381	4.993802858	4.82721898	5.047533654	4.631735685	4.850063779
5.781635541	5.633862953	5.548935147	6.227085889	6.106370709	5.936291499	7.174089047	11.41261035	6.471979852	5.879578943	5.69883129
8.397908167	6.933333385	7.117966813	8.633840518	9.254362124	7.87732225	7.820832211	7.755638768	6.932764458	8.618594178	6.926467459
6.778222061	6.641739093	6.691646104	5.882548491	7.301218632	7.452686268	7.812169014	7.751414094	8.150954367	8.794525299	8.9843958
9.887366763	11.99377713	13.07525448	10.23903152	6.189676925	10.57411641	7.04015668	7.043765894	7.560912722	8.644236833	8.929111774
8.015683441	9.879275563	11.8497032	10.18705417	6.826245699	8.977724119	8.261458674	7.639709085	8.180706253	8.353842117	8.713681182
8.374159259	10.94027721	12.70311159	9.230503026	6.408597919	8.714803114	11.71834394	7.362699397	11.94589693	11.45069487	12.02565907
8.374159259	10.94027721	12.70311159	9.230503026	6.408597919	8.714803114	11.71834394	7.362699397	11.94589693	11.45069487	12.02565907
6.928821127	7.217318804	7.678893793	7.01848526	6.794092456	6.903946509	9.580442021	8.655654248	10.73418329	7.874054383	6.382065089
6.101143867	7.626299638	10.39906406	6.4400009	5.820604156	6.376709102	11.24552894	7.883274537	11.63915268	9.092086056	7.275790726
7.008533954	9.304317226	12.06165882	6.169550325	5.795414961	5.414101162	11.91800498	10.11774873	12.51957381	10.19037154	7.356592131
10.03639427	11.06587336	11.57420893	9.157825652	7.835035046	9.070447807	10.55619935	8.827227535	10.97862882	9.605181998	9.204400101
10.47371668	11.46383398	11.81474509	9.548046854	8.519921189	9.186678467	9.163779479	7.844792569	9.372104355	9.063073788	8.931783795
10.1584471	11.74050735	13.40707133	10.59923097	7.174444283	8.98512949	9.964084667	9.400848742	9.227108319	8.986673713	9.856919121
8.812525163	10.33284528	12.86476602	9.852140247	6.268938529	7.468480723	6.904337297	6.632895524	7.900348423	9.871923165	9.341822101
8.628532728	8.49142056	9.416018279	10.04315043	7.917781403	9.708236012	7.188452931	6.484104835	7.617226242	9.332144617	10.26622706
7.82449345	7.790061383	8.967702264	7.852551625	7.435115273	7.371908846	8.529449148	7.732769267	8.697718365	7.726869649	7.832157088
8.693541328	7.904814385	8.30593707	8.860353229	8.350406574	8.145301515	8.959293004	8.15579566	8.175484613	8.567003759	7.78394216
5.64593778	6.35575016	10.10295707	4.79508224	5.116813904	5.003573365	10.50286754	6.910012167	10.87502435	7.052531735	4.925351167
5.389683214	5.973963694	8.381752355	5.659389559	5.266093829	5.090652939	8.264560237	6.519168139	9.45356578	7.684602939	5.953086515
5.493579871	6.194203146	8.040042189	6.168164589	5.740544373	5.571078585	7.1020399	6.690361764	8.18512495	6.629423436	5.951870785
6.457076442	6.104944575	6.309741674	6.665678565	6.460448381	6.404406114	6.958101215	6.216069784	6.547658733	6.437125979	6.154541533
6.808282203	6.46529511	7.159131986	6.650586731	8.252537696	7.03341495	8.008466833	8.273639854	8.915287929	7.055244417	6.903665991
7.13667614	6.419703263	7.632856401	7.343191678	8.741218418	7.87803013	8.719221378	8.561859692	9.320715007	7.621782031	7.244183317
5.695353996	5.907429494	5.840944486	5.346397102	5.50026178	6.085558895	5.716101076	6.328072395	5.743325573	5.620611076	6.091472097
5.448671895	5.371011146	5.333610661	5.414477312	5.547811825	5.39772526	5.93505726	7.105365718	6.101525306	5.287296625	5.241945788
5.974527954	5.983754212	5.946945133	6.608651721	6.36942628	6.400525595	6.464973728	6.355860776	6.015483397	6.446411792	5.797965503
6.214756457	5.656789362	5.982096968	5.863962224	5.426481179	6.648391628	5.308555445	5.154149833	5.323294543	5.478010444	5.508552997
6.214756457	5.382695449	5.90038085	5.845807069	5.361201486	6.648391628	6.02012797	5.320745834	5.412251653	5.160986954	5.349217191
5.547062957	5.355639715	5.569605681	5.944408789	6.206964204	5.262243184	5.597333294	5.174534756	5.372799536	5.132766655	5.721927193
5.391254309	5.283060745	5.387175526	5.437182469	5.395002504	5.501397751	5.652266653	5.222030732	5.252888843	5.457361462	5.288612315
5.391254309	5.281962185	5.435525138	5.455057758	5.58991697	5.537729538	5.92423551	5.332298465	5.471220939	5.457361462	5.330998361
5.391254309	5.281962185	5.435525138	5.455057758	5.58991697	5.537729538	5.92423551	5.332298465	5.471220939	5.457361462	5.330998361
5.288609652	5.419023798	5.489966196	5.582738392	5.17779869	5.355971969	5.911251615	5.05243864	5.193739307	5.761752115	5.324800247
5.39499302	5.236427767	5.070467477	5.630583639	6.963003392	5.639931858	5.436271551	5.347346143	5.053776464	5.972022271	5.393672132
5.853557447	6.080665502	6.3630761	6.689525611	5.822327939	6.328788931	6.239878477	6.329265873	6.049807971	6.247901295	6.347820769
6.555458869	6.218072516	6.243385297	7.04294987	6.86356995	7.138555731	6.998237079	6.833996999	6.945616606	6.531227065	6.654026521
6.669781474	7.5725396	7.294092449	6.898877063	6.440307993	6.529212682	6.638277202	6.680958678	6.136198534	6.353166465	6.535327279
5.570000019	5.515730297	5.78189192	5.989113508	5.657568917	5.7723695	5.569777729	5.547765634	5.345432969	5.522220694	5.735138278
5.851830077	5.705079845	6.360222271	6.466468212	5.683117847	6.246175149	6.154345119	5.878598834	5.606187828	5.871282416	5.797992852
5.12088855	4.729663188	4.750048425	5.195364236	5.232984683	4.997376133	6.567248023	6.665871258	7.725411244	7.472123477	8.654043217
5.458404222	6.692447257	6.476162771	5.505844835	5.283641491	5.286127304	6.55891305	6.891455416	7.180467691	5.356073676	5.128573994
4.922227703	6.134261247	6.169565102	4.934454594	5.16625179	4.705122001	6.170346413	6.396784121	6.59849843	4.77567138	4.934740295
5.000068908	6.271598947	6.399371841	5.114922442	4.939137839	4.919032411	5.845355709	6.538930608	6.372858943	5.312185586	5.254327927
5.40412199	5.693375749	5.308147205	6.560982264	4.782102499	5.303795818	4.950268415	4.979134179	4.833363696	5.906593803	5.182750692
6.372136092	5.747292115	5.659024798	5.971824551	5.987823889	5.685965103	6.144838925	5.974267209	5.856200721	5.920753766	5.644295402
5.430181392	5.466502285	5.442178798	5.69058132	5.605698099	5.802102088	5.775272878	5.360651644	5.111297061	5.551959083	5.417404166
5.626871462	5.367269814	5.42820761	5.818584642	5.372446626	5.560533685	6.366111307	5.229747355	6.42711692	5.553329273	5.611212917
5.574359013	5.361804583	5.460195351	5.592653004	5.405333908	5.550781895	6.089278169	5.227418049	6.610982025	5.704019098	5.486752667

								Tassel and Anthers			
V5_Tip of stage-2 Leaf	V7_Tip of stage-2 Leaf	V9_Immature Leaves	V9_Eleventh Leaf	V9_Thirteenth Leaf	VT_Thirteenth Leaf	R2_Thirteenth Leaf	V9_Eighth Leaf	V13_Immature Tassel	V18_Meiotic Tassel	R1_Silks	R1_Anthers
14.9597247	14.93030013	14.71119165	14.98487679	14.76304437	14.99438767	15.11359589	15.1201473	15.06172001	14.81218859	14.90280327	14.60959308
11.83977831	10.70141189	12.82964421	12.37583469	12.39140257	11.65915384	11.38570891	11.31130257	13.07146913	14.40486644	14.96811654	13.45873038
14.17365429	12.9997975	13.4688125	14.38538395	14.67669803	13.66598284	13.75736399	13.9236243	9.357628774	12.94911972	15.103246	13.2691526
13.53070748	12.82397698	9.446569296	13.66669373	13.80330407	13.34174241	12.94435379	13.10737729	7.567943842	9.752526862	9.515747237	12.20620577
12.65365465	12.30542211	13.3240019	14.34252694	14.35508976	12.00784129	12.22562225	12.39983157	6.091749759	14.82119682	13.40817808	13.80026292
13.29565894	12.95944117	14.02280114	14.82776609	14.88685895	12.80153651	12.8590679	12.96540307	6.466846922	15.12580974	14.05248273	14.35060524
11.95014789	10.79809806	13.95107309	13.7468713	14.22067797	11.50047445	10.92381175	11.13907023	9.2309582	14.14598967	14.89998938	13.24548023
12.42758497	11.09020155	14.00116719	13.86861496	14.3712011	11.63156466	11.14464814	11.28656626	8.839798998	14.21773499	15.25473371	13.32755426
5.812260725	6.355135592	5.73256136	8.252107156	5.899061014	6.06236394	6.053685986	8.037859167	9.91357998	7.216920431	5.721762175	6.210742536
6.588328382	6.713795619	12.398633	6.513061389	6.947419616	9.184493648	8.692411258	7.981012055	13.51069158	12.75276362	14.68211991	10.35604821
11.107863	10.73614633	10.9902875	13.61495349	12.52043872	10.12434403	8.938618311	10.95241068	10.59375669	12.31242556	8.96329943	8.898158013
11.107863	10.73614633	10.9902875	13.61495349	12.52043872	10.12434403	8.938618311	10.95241068	10.59375669	12.31242556	8.96329943	8.898158013
9.005973447	8.062749214	12.52350583	11.93601858	12.48705643	7.626942864	6.831221892	6.853133544	11.46298768	10.87724628	9.596807908	7.302348091
8.87155161	8.232275954	11.60473195	10.54444248	10.21421132	9.348507726	9.753086707	10.15683321	11.28592106	11.91402669	13.19283978	12.47827088
9.337485195	8.902403284	12.02590836	11.13670214	10.71490593	9.988879016	10.24310767	10.66269173	11.84291049	12.41873441	13.5073827	12.96802246
10.36632241	9.171418934	9.802910419	10.38898175	10.15157196	9.031325848	10.99356851	9.313481325	9.488889142	11.36312624	10.68727245	11.85611912
10.28722919	9.85439412	13.03715329	10.83200804	11.72338817	10.30132528	10.11706527	9.674548245	11.77721965	12.00999188	13.41851879	11.14138363
5.915741526	5.768598408	13.92974871	9.973874903	10.64491679	6.162470984	6.896647397	6.244511439	12.40242385	13.4584896	12.23229191	12.71823903
5.95561301	5.661939488	14.46484117	10.17662269	10.99017194	6.115468581	6.678634565	6.276133002	12.80238206	13.93396083	12.63957776	13.16924277
6.771310989	6.067589753	13.01138213	12.03823599	11.35391953	6.744236669	7.417718434	7.856280428	13.89026025	13.09864222	13.39845606	10.63603262
6.74263329	6.293008174	13.02163649	11.85005773	11.17427377	6.723356882	7.379544372	7.389239655	13.8359659	13.05830152	13.49419596	10.55581679
6.74263329	6.293008174	13.02163649	11.85005773	11.17427377	6.723356882	7.379544372	7.389239655	13.8359659	13.05830152	13.49419596	10.55581679
5.029469174	4.826531647	4.847846771	4.819739848	4.889385638	4.829258953	5.126378448	5.022806093	4.930214044	4.810540732	4.949311537	4.81694193
5.547689713	5.202995939	5.417545991	5.422047448	5.216547539	5.459661314	5.596569509	5.454131763	5.608555894	5.460591614	5.574978922	5.474335803
5.13357015	5.609932633	5.6461326	5.128370768	5.095303306	5.537012251	5.432468196	5.61182309	5.53278803	5.177711292	5.705518059	5.200973444
5.565236349	4.893234237	4.799097538	4.875252334	4.924237238	5.210743912	4.969193522	5.069548344	4.83055188	4.80033712	5.532767704	5.049923279
5.010983879	4.749885814	4.703716804	4.672974036	4.563992043	4.88610728	5.259788533	5.135080436	4.620389874	4.575763193	4.573674166	4.584433717
4.997572404	4.70846165	4.738643959	4.669611896	4.740271021	4.741842568	5.34847632	5.034279628	4.585980486	4.658523225	4.715571066	4.653444625
5.962353071	6.563164517	5.919655784	5.613718227	5.534085101	7.659491746	9.495593471	6.332742752	5.981089654	9.157938671	6.912492121	13.56167982
7.540533317	8.2078455	9.567262246	8.576513418	7.651901479	9.050081237	7.950534483	8.009501811	10.79115411	8.787803074	9.056929623	8.421544813
7.118371665	6.825196083	7.726532432	6.968100669	7.43162373	6.796191954	6.515637859	6.879229023	7.649272387	10.40596003	8.33827039	8.743904406
8.053591714	8.510853551	7.16335552	10.27873848	9.511780431	8.338076401	7.833152184	9.501598371	6.231585204	10.1670937	5.939551371	5.942168906
9.8239361	9.776399057	8.254082611	10.95668634	11.15257643	11.28972099	11.50621365	11.83756881	7.402658096	10.9771497	6.045319598	7.54364466
12.08796786	9.966553633	11.68615779	13.22397304	13.51178985	6.419383911	6.612686419	6.79401451	6.88807812	13.27103099	10.63908808	7.461660137
12.08796786	9.966553633	11.68615779	13.22397304	13.51178985	6.419383911	6.612686419	6.79401451	6.88807812	13.27103099	10.63908808	7.461660137
9.593862673	9.334656897	7.565226985	10.97587562	11.60422397	9.790749749	9.190770076	8.988457575	6.561188377	11.69157853	7.801616656	11.75880254
7.343590297	6.512571459	12.72540383	9.491077255	12.22539015	6.496466509	6.258928056	6.528069871	6.011110377	11.59826076	6.956372123	9.846144429
8.090150684	7.776532075	12.7201065	9.295509773	12.11130246	7.827688172	7.03268172	7.866732065	5.773638699	11.75454862	6.25216231	11.69484509
8.667984538	9.317219865	9.736064807	10.87682603	10.56247961	8.815080658	8.398891639	9.039336438	8.831706706	11.36706424	7.818791598	11.01846172
9.883152125	9.969704543	9.516998302	10.67231889	11.36889907	8.287307824	7.408203459	8.346187707	8.606145003	9.391726064	7.973704593	7.255445807
9.243943444	9.122099636	8.856165543	11.45131609	12.01056375	9.244985328	9.324459369	9.578122303	7.265975995	10.03805419	8.80359866	7.587384809
7.133135148	6.023808633	8.803823257	11.01400701	10.31427457	6.58332883	6.555561132	8.29748431	6.335043028	8.179251486	8.707606332	6.275402565
7.243883646	7.402754689	9.297818702	6.802565297	6.819742673	7.203582935	6.810178297	6.701466105	7.924784268	7.343680788	11.48756504	7.092442388
7.660852493	7.169780133	7.901736304	7.511025833	7.705585413	7.436392888	7.51502176	7.419506328	7.482579773	8.024094365	7.849474564	7.970379028
8.496591169	9.072477472	8.903650415	8.604569464	7.921665384	8.889201535	8.113768954	7.71843301	8.498005077	7.706467416	8.388693892	8.067358178
5.607226294	5.023116265	11.52149855	8.698438211	11.69118764	5.151794144	5.300104995	5.208948571	5.098003082	10.44236155	4.784881892	8.319840541
5.65910216	6.039762542	9.241686249	6.57146251	8.538415953	5.65806661	5.710851635	4.903431342	5.049392827	7.413654599	5.816959232	7.133588966
6.067628777	6.574073085	9.132303924	6.550597234	8.624346835	6.280258772	5.823613236	5.520536855	5.771344901	7.605836846	5.756536904	6.755167242
6.395721912	6.188388498	6.73838947	6.783280551	6.35904485	11.52113785	10.45458217	8.404449833	6.391104518	12.71128907	6.952205245	6.103173137
7.136305745	7.869168128	7.009943416	7.299664587	7.14960928	7.526165863	7.627150478	8.710147379	8.057898805	15.36595562	7.458226004	6.905407414
7.372036147	8.448778087	7.956565585	7.914898658	7.589778629	8.092587796	8.584288605	9.27655723	8.828002207	15.66203004	8.255164842	7.449369828
6.366031559	5.362909191	5.356163487	5.772022313	5.71320101	5.841812411	7.36191216	6.945555071	5.55744959	14.83454246	5.973387046	6.162505108
6.497724877	6.179846884	5.558943028	5.504743855	5.419029279	6.165406403	5.943359803	7.573643765	5.381181999	13.445679	5.835223015	5.471668817
6.795318993	7.123370377	6.255519949	6.1016178	5.915193428	7.232618012	6.767177073	6.448748842	6.311787751	12.71599437	7.069270087	6.097095638
5.578319038	5.489883368	5.271082526	5.488659262	5.18456911	5.265108517	5.869499268	5.913360316	5.559878101	15.54068988	5.245787062	5.264925993
5.818074569	5.949398797	5.283873557	5.535995749	5.372648523	5.537357876	5.85352681	5.985003042	5.720522315	15.54068988	5.245622653	5.295077769
5.324411824	5.2943752	5.490746078	5.246918397	6.344461281	5.567075166	6.009556532	5.559167524	5.782825793	11.75000723	5.229010636	5.346293203
5.409365566	5.438920088	5.663093333	5.395028333	5.320009612	5.490675322	5.371656319	5.317031381	5.258560273	10.0845582	5.669606724	5.208729703
5.409365566	5.438920088	5.693906425	5.462140111	5.214755647	5.627837377	5.371656319	5.286419575	5.221725834	9.134622849	5.669606724	5.169465323
5.409365566	5.438920088	5.693906425	5.462140111	5.214755647	5.627837377	5.371656319	5.286419575	5.221725834	9.134622849	5.669606724	5.169465323
5.675912984	5.978790584	5.598984623	5.599481411	5.097635955	5.817478534	5.055661982	5.11945189	5.196430363	4.885853252	5.293544151	5.568485007
5.450588627	5.787251109	5.501901882	5.405029281	5.243716194	6.343874871	5.496642513	5.754203926	9.213993519	6.77666946	6.515636091	5.469283286
6.630812956	6.97736962	6.069781839	5.973276967	5.848928008	6.529119825	5.888420763	5.793692698	5.995404186	5.783015447	6.334850513	6.385747337
7.231235565	7.470913815	6.454650661	6.779315212	6.541026991	6.533125798	6.257933922	6.657022296	6.375120826	6.287091248	7.461584675	6.510282378
6.705585481	6.878171167	6.443024756	6.090697196	5.806824638	6.787644436	6.457178848	6.153692966	6.016066977	5.780718121	6.352271432	5.975684134
5.603049829	6.548486016	5.464407735	5.441069129	5.202173045	5.597504516	5.435903614	5.125687416	5.26338205	5.226333601	5.553141892	11.787766
6.0281198	7.148639476	5.690837499	5.627026924	5.226699624	5.948393516	5.813497041	5.349978755	5.60248638	5.509641387	5.80685238	12.89397843
7.697843563	6.301223726	5.606840344	4.93777191	5.168324082	6.100436633	7.043429122	6.35066611	4.913480936	7.573196969	5.081933316	5.324531111
6.262882501	5.558548568	5.609967875	9.378791684	7.303501806	7.065291743	5.561996721	7.48376594	5.032429014	5.043017334	5.78730943	5.143906516
6.542419862	5.075059247	4.900522186	7.539446211	5.353774131	6.997695267	6.576071693	7.754053241	4.691885232	4.744711433	4.835107885	4.852561609
6.69846715	5.594811775	4.881605121	7.650595043	5.416466773	7.190871923	5.901203827	8.016481917	4.851128466	4.760324548	4.852571076	5.099098958
4.890568027	5.307043574	4.90444267	4.884966542	4.726860115	5.356518906	4.996009984	5.03575005	5.408751291	4.904736216	5.428256101	4.926687516
6.138132414	6.388399744	6.484156825	5.829032921	5.770223145	6.196940156	6.195680596	5.938177941	6.160165665	5.793209642	6.661236694	5.909897662
5.349925946	5.600060937	5.367950097	5.32767562	5.146091739	5.744942134	5.15425368	5.232956971	5.55258984	5.078089252	6.40032762	6.497465223
5.392820088	5.948855644	6.989064415	5.844119799	6.519379773	5.322860174	5.566202677	5.600585398	5.503820411	5.630157233	6.024199415	5.576525573
5.649317214	5.905439673	6.975179327	5.929689152	6.423477477	5.542467537	5.532537077	5.827971002	5.802541257	5.612101166	5.844820036	5.550570846

Husk			Whole Seed						
R1_Innermost Husk	R2_Innermost Husk	R2_Outer Husk	2DAP_Whole Seed	4DAP_Whole Seed	6DAP_Whole Seed	8DAP_Whole Seed	10DAP_Whole Seed	12DAP_Whole Seed	14DAP_Whole Seed
14.85820025	14.87294354	15.07281125	14.97340243	14.7738447	15.12955781	14.86449684	14.88814484	15.07322062	14.93413719
13.78050577	12.83318937	12.63798483	13.63128377	14.5036069	14.6534721	14.56176277	14.15834008	13.76500721	13.22457499
13.6654973	14.26010892	14.24028648	11.32644985	11.86529675	12.36856391	12.35428705	12.50563679	12.71321473	12.53803751
10.92979227	10.47891867	12.72569177	9.147549857	9.843473681	11.06417842	11.73541433	11.86872342	12.04703535	11.47495771
14.28113388	15.14775821	15.22979689	10.05687744	11.74600577	12.69207674	13.28811939	13.11770181	12.48217207	12.25401142
14.85653631	15.26209023	15.53339491	10.85781574	12.32758746	13.33839359	13.95039446	13.811861	13.31499892	12.87590967
14.62729072	14.96903419	14.42720869	12.27235914	12.90552099	13.3117262	13.37958226	13.33363093	12.46645322	12.10076969
14.96104023	14.93652623	14.63528528	11.76929884	12.84574458	12.80515181	13.09525095	13.16553744	12.50861712	11.88419179
6.206010788	6.118903933	5.999877403	12.651856	12.35496527	12.12285402	11.48951604	11.26994096	11.28572168	9.985186377
12.25584442	6.893531653	6.933167215	13.87676091	14.09557506	14.2427099	13.71932993	12.69046996	11.21377752	9.704733046
10.19944984	8.810616526	8.035759591	11.37190865	13.34448329	15.18255748	15.32303111	14.79876203	13.85806815	12.15234875
10.19944984	8.810616526	8.035759591	11.37190865	13.34448329	15.18255748	15.32303111	14.79876203	13.85806815	12.15234875
11.79418431	10.878331	9.638054262	11.53489707	11.4603532	12.30235401	12.1279318	11.92695757	12.84789896	12.0458376
12.74620905	12.52651327	11.97872937	11.81679176	11.77006923	12.48452107	12.03732772	11.87782708	11.70008398	11.36497242
13.19583492	13.22397093	12.41082346	12.29212147	12.29126749	13.00186889	12.52219689	12.45691707	12.16060705	11.81285388
11.63439069	10.80232752	11.71745344	10.93154359	11.23425757	11.87220695	11.33888446	10.96164581	11.60271627	11.87295055
12.40567837	11.51893938	11.09300609	12.59475654	12.94377991	13.19176339	13.10620687	12.89637633	13.02390086	12.9039104
14.25919387	9.995887882	7.369760944	13.95260464	14.57536376	14.52921567	14.33467049	13.87904721	13.66179205	13.15847481
14.45335425	10.25366885	7.110926588	14.32469214	14.94488441	14.84519565	14.67523008	14.39810587	14.20122123	13.65817594
14.03739625	12.97850612	12.53724068	13.9367448	14.00432664	13.84015457	13.48692685	13.24903124	13.18944185	12.95881872
14.19414976	12.89792368	12.47397671	14.17958296	13.97102252	13.83773624	13.47011026	13.20792216	13.12080865	12.87191122
14.19414976	12.89792368	12.47397671	14.17958296	13.97102252	13.83773624	13.47011026	13.20792216	13.12080865	12.87191122
4.808163758	5.155443686	4.875266885	4.782827969	4.911815864	5.709304491	13.27767497	14.63191088	15.12494954	15.14461667
5.23045036	6.456201834	5.654366093	5.314698164	5.574952751	8.264388297	12.78073441	14.25897644	14.97693347	14.73787558
5.313835076	5.037659634	5.824501108	5.175531277	5.286027962	6.180794373	12.88224081	14.81483844	15.24908061	15.05239123
4.966452081	4.906648697	5.033444488	5.208263901	4.867586585	4.849570337	5.066453239	5.218925441	9.076859911	11.14547682
4.743949133	5.577881621	5.14314077	5.15408403	4.845958142	5.121358452	4.84072766	10.08647269	11.9483811	11.83126031
4.690961627	5.27743193	5.22868372	5.005590008	4.799912573	5.015140175	4.970995959	10.0520633	11.91450034	12.15282113
8.130278497	10.88473363	12.59099362	7.14294201	9.160954739	11.43913846	10.92355729	9.612398164	9.03835389	9.440896378
7.791445441	7.491187745	7.975577533	8.241422718	7.748852595	7.533594617	7.910034056	9.50503509	9.775345898	9.548788862
7.784912654	8.06630025	8.035380413	7.634580278	7.928638051	8.345260827	8.608817375	8.552435663	9.885322573	9.190933044
8.867910735	6.10127079	6.062137559	10.92850939	11.31533397	11.46460567	10.0273366	8.004942765	6.153838844	5.473785694
8.019095044	6.437359141	6.299656521	9.137242651	9.436138601	9.735730199	8.34863157	7.317110304	6.439568186	5.737784722
11.16566309	9.313036052	7.634585456	9.155102818	9.796715866	10.01299639	8.695056098	7.552991311	6.613546136	6.799124289
11.16566309	9.313036052	7.634585456	9.155102818	9.796715866	10.01299639	8.695056098	7.552991311	6.613546136	6.799124289
9.434543461	9.600091176	11.10553501	6.987917146	7.487451297	6.967611725	7.533775756	7.716473831	7.169207993	8.242348239
10.83843785	6.476732954	8.031278522	5.680483406	6.373750368	9.087144588	9.609257074	10.37260407	9.336099963	7.987305564
11.8913404	8.574122691	10.91142213	6.126712258	6.639336992	8.644331736	9.202778792	9.475875477	8.437267336	7.013702486
10.23265268	10.77834886	10.62401027	9.583088969	9.778545031	10.01043562	9.746896605	9.748402432	9.814301823	9.596928507
8.611495062	6.928648593	7.58883393	9.021222095	8.965422526	9.339940169	9.02235004	8.597023492	7.966395895	7.494507805
9.323288415	8.170655016	7.646497428	8.300708897	8.808216609	10.00355779	8.232687016	8.379098044	7.405962559	7.576121154
7.317208284	7.036882389	6.314887472	7.631017876	7.909234553	8.459554071	7.53398325	6.701820702	5.945916202	5.831862785
9.289936031	7.181017309	6.922061761	9.588429261	9.810413274	9.59321106	9.017454576	8.469521533	7.607117545	7.45285606
7.953708922	7.72732645	8.008437865	7.588406245	7.413728415	7.708353883	7.924109391	7.889866537	7.822176913	7.729507487
7.853667232	7.734787781	7.877530214	8.173222549	7.725313681	7.537657348	7.712037711	8.371639962	8.101646412	8.321666243
10.24911956	5.485020806	7.890442031	4.937391666	5.685793535	8.250922665	8.416998682	8.893979324	7.051782126	5.807812294
8.500357535	5.246769608	6.339749058	5.312608621	5.414945494	5.827557481	5.641937638	5.541967929	5.599966529	5.125074808
7.696525193	5.3000528	6.413723153	5.79311149	5.296457863	6.276961898	6.402592942	6.621715554	6.422650423	6.140402669
6.127516543	5.848873612	6.388246937	6.235970786	6.286802313	6.325486031	6.44549271	6.286581866	6.269732746	6.137067544
7.045661656	7.4845278	8.421433896	7.393136834	8.052511179	8.552090455	7.523376057	6.927169026	6.632114176	6.707883775
8.006166638	8.159595184	9.218896848	7.897943631	8.674237334	8.828333566	7.986473705	7.321936483	6.972997215	7.324904778
5.794456084	6.594257948	6.417301692	5.873513265	6.28719129	6.705536503	6.654061981	6.460594009	6.363451971	6.749216145
5.354171375	5.111109975	5.46434985	5.170290449	5.074922223	5.180055829	5.015612786	5.272150834	5.418162683	6.136278537
5.890616175	5.270539028	6.146517855	5.721077559	5.633434852	5.50260986	5.666063034	5.794459213	6.261131564	6.506010197
6.857256151	5.476094281	5.330174958	6.777629327	6.894763493	6.173397542	6.567124183	6.13682518	5.494381879	5.22010872
6.857256151	5.324273996	5.497614678	6.663501347	6.655976181	6.21804988	5.908369986	6.213524328	5.193912944	5.231917352
5.290923268	5.403747978	5.590590145	5.2138827	5.402969258	5.26757479	5.331975228	5.056915139	5.909793434	5.854085314
5.268929241	5.100752277	5.446319265	5.330367784	4.97015546	5.087993561	5.470820758	5.343750796	5.409145397	5.767244364
5.217383994	4.90336552	5.556624511	5.42465923	5.033153323	5.083245043	5.324040263	5.508619267	5.582562211	5.625214281
5.217383994	4.90336552	5.556624511	5.42465923	5.033153323	5.083245043	5.324040263	5.508619267	5.582562211	5.625214281
5.193418927	4.98848755	5.34259685	5.192484461	5.032085688	5.034960575	5.191708202	5.043301065	5.470668693	5.74714308
5.515460847	5.125269985	5.385200704	5.686155117	5.77776766	5.53087883	5.563566615	7.353052	8.49175628	7.426877326
5.936111351	5.537735368	6.172955649	6.100788471	5.386093748	5.802118638	5.574654407	5.96612026	5.711794362	5.897046818
6.700695577	6.115019978	6.710474601	7.227444077	6.708515531	6.583591874	6.218524665	6.2408575	6.086743496	6.025683649
6.160161836	5.838491188	6.18093035	6.691284037	6.904136353	6.375719095	6.690427603	6.398014999	6.141728226	5.875576261
5.248920393	4.921844882	5.31152655	5.303522611	5.101402581	5.084893291	5.135627114	5.371189497	5.371007826	5.700818251
5.550980204	5.177831973	5.422037427	5.317887667	5.250549912	5.093917329	5.177612317	5.561250377	5.528222262	5.913886437
5.292060906	5.382451311	5.310653218	5.360660446	5.355304388	7.259632584	7.870687297	5.94151111	5.69891225	5.485447187
5.42564279	4.846695149	5.402227379	4.917858131	4.743920404	4.866073368	4.782334648	5.179271947	5.341616734	5.273531794
4.799427895	4.766788005	4.872656375	4.876430892	4.763107889	4.936670205	5.004614594	4.718334808	4.793620761	4.893510013
5.360281261	4.877991907	4.953010984	4.906737617	4.825328351	5.131472212	4.859136642	4.95748265	4.922941504	5.266987183
5.573287075	4.915439237	4.916345191	5.239529276	5.063680324	4.918039686	4.803830523	4.711299373	5.144604397	4.866770777
5.686374858	5.691397006	6.15099357	6.104425532	5.44545869	5.664049053	5.597647286	5.920409652	5.582590395	5.894463265
5.292511274	5.027606501	5.557970336	5.253234051	5.143279304	5.010217964	5.055085134	5.848149862	5.355202806	5.332646003
5.656197946	5.287843862	5.514746849	5.311735868	5.356769755	5.354118769	5.129142999	5.406262268	5.210018204	5.503676106
5.632696989	5.670248822	5.591563343	5.159763225	5.241985108	5.01040395	4.99779199	5.49110765	5.19410247	5.968914162

					Endopsperm					
16DAP_Whole Seed	18DAP_Whole Seed	20DAP_Whole Seed	22DAP_Whole Seed	24DAP_Whole Seed	12DAP_Endosperm	14DAP_Endosperm	16DAP_Endosperm	18DAP_Endosperm	20DAP_Endosperm	22DAP_Endosperm
15.06384415	14.83026451	15.15673103	15.0266319	15.09183031	15.02821342	14.98584308	15.09509072	15.18066035	15.22506189	15.17645698
13.34486485	12.74903539	12.99354799	12.71473945	13.26806529	13.10055179	12.35614274	11.49721888	12.80589958	10.16701224	9.588437202
13.09499874	13.1878255	14.02126036	13.86045715	14.54153162	10.14042469	10.17091552	11.04598426	13.42902448	12.01843176	11.76811975
11.6329387	11.26651555	12.49275006	12.29710991	12.89744356	9.682572983	8.86574951	8.473634069	11.00396757	10.19484216	9.635453675
11.74765887	11.6183333	12.16193528	11.2816733	11.16160083	7.921376894	7.87151928	8.813876787	11.56092381	7.910352913	6.877561421
12.59917398	12.32927903	12.86613678	11.95736589	11.82806659	8.417356854	8.418542048	9.332852887	12.26637557	8.30617093	7.224429444
11.90665849	11.52594825	11.51954875	10.16028608	10.705642	8.773374095	8.367598345	8.472591555	11.24401898	6.65348555	5.86531087
12.1566726	11.60931032	11.7465065	11.14644557	11.54620989	8.477065496	8.351018905	8.578594662	11.41797171	7.996534003	7.833575876
9.22456944	9.279315733	10.05110155	9.580566077	9.979793942	9.351711956	8.72292134	8.160730189	9.288182739	6.673814506	6.658896789
9.458784953	8.99000785	9.976544582	9.96422162	10.78729812	8.867721351	7.780529436	7.767944735	7.465783831	7.298271705	6.837419636
10.30880985	8.517449522	8.470109663	8.262841166	10.05440006	11.88478618	8.852310648	5.567275626	6.211534299	5.783857484	5.710149274
10.30880985	8.517449522	8.470109663	8.262841166	10.05440006	11.88478618	8.852310648	5.567275626	6.211534299	5.783857484	5.710149274
12.14683819	11.31939364	11.85225586	11.5456905	11.81088363	10.71594333	9.767633942	9.334928318	10.90331779	7.593719466	7.542929145
11.06166048	11.08657892	11.31948609	11.07382227	11.00401486	10.76203071	9.740699168	9.63095775	9.971504705	9.566848522	9.391911597
11.41785806	11.51045505	11.75356761	11.55364609	11.48790168	11.15058432	10.11913513	10.03280127	10.36664705	9.858186569	9.913309865
11.84976773	11.23812714	11.47335762	11.23519012	11.61448589	12.1955701	12.11652861	11.53555214	11.70936653	11.41379774	11.32932142
13.17126872	12.24093556	12.47188052	12.2012656	12.43648006	12.80123157	12.30094041	12.79957967	12.48535345	12.59127248	12.63007902
13.05352087	12.70722354	13.04456751	12.55390024	12.8000097	11.79659073	11.23837213	11.50630409	12.9286909	10.00150116	9.990865787
13.52977666	13.19019605	13.6149214	13.02068912	13.30382333	12.1520866	11.62431196	11.85082333	13.38022867	10.21727266	10.31985983
12.7650619	12.71054285	12.89021968	12.42335173	12.74413512	12.97187922	12.61782924	12.33291905	12.84644997	12.504155	12.07395189
12.88210211	12.59784404	12.86376875	12.41732519	12.73538717	12.98249403	12.61248573	12.27900234	12.84489944	12.23018251	12.04093552
12.88210211	12.59784404	12.86376875	12.41732519	12.73538717	12.98249403	12.61248573	12.27900234	12.84489944	12.23018251	12.04093552
14.9888324	14.99328766	14.47280062	14.01742834	13.6152201	15.26018153	14.86262184	14.47647755	15.06412292	12.70161089	12.30534356
14.5499082	14.06798495	13.76874863	13.79814854	13.97649035	14.47604732	13.23894482	13.09275998	12.41494666	13.26834629	13.59116498
15.23444645	14.98010616	14.37909323	14.61508001	14.2827855	15.04142752	14.10615163	13.71165917	13.16002921	13.90185412	14.23679341
12.37752197	12.05071159	13.1813748	12.80632073	13.39114791	8.29943112	10.42191814	11.09028429	12.73105436	10.53753846	10.60545212
11.00165657	11.31718087	8.646537614	8.961461752	8.852193591	10.56278024	10.02970301	7.968824197	9.194986642	7.149065588	6.724355045
11.62170048	11.33119927	9.026632651	9.592509588	9.164037121	11.11711785	10.14758981	8.945144364	9.477524936	7.474405862	7.418839926
10.09286641	8.833261185	9.762469599	9.128262852	9.736703031	9.10524876	8.813818475	8.332898423	9.986872286	7.791351285	8.248428946
9.783910581	9.247628516	9.453467174	9.392155055	9.897784426	10.54862863	9.797960233	9.585669752	9.330071641	9.423619973	10.41768369
8.933259566	9.767411267	10.26635512	10.09996289	10.65972257	9.327885178	9.203506499	8.648916211	9.677310315	9.44036251	9.640822195
6.545180239	5.303418861	5.524957127	5.573702147	5.380032665	5.24446322	5.452761098	5.7323788	5.227756866	6.103214259	6.174066857
5.538932759	5.526925916	5.584140218	5.556731368	5.383750507	5.490968567	5.609391128	5.920944983	5.755029409	6.432445087	5.710015616
5.743478157	5.505936456	5.580560878	5.459354013	5.827981267	6.229132455	6.114060567	6.148688259	5.203773008	5.439483251	5.721168741
5.743478157	5.505936456	5.580560878	5.459354013	5.827981267	6.229132455	6.114060567	6.148688259	5.203773008	5.439483251	5.721168741
7.957993335	7.558909445	8.258226661	7.484601539	7.874461875	6.240557957	6.38234447	6.383612407	6.711978026	6.518591404	6.066376979
7.261281783	6.83277921	5.960795743	6.007511207	6.366108046	5.892016766	6.016301346	6.50357235	6.162209952	6.285462154	6.007428006
7.079524053	6.242208805	5.563762077	5.912772969	5.802036448	5.92738933	5.778275514	6.051077486	5.519638316	6.704928863	6.32886268
9.364367666	8.876930175	9.152361902	8.902016266	9.119767155	8.900718612	7.983698779	7.869147743	8.488222289	6.933572126	7.358553722
7.696507575	6.850310908	6.911116382	6.812276658	6.69809812	8.127879727	7.054558154	7.173565844	7.327269922	7.3237515	6.368782543
7.46150395	7.479660721	7.682732767	7.718589096	7.968045329	7.908421599	7.904922157	7.849488732	7.724821543	7.475012364	8.016580566
6.204350065	5.703327717	5.83082585	5.827309306	6.012455113	5.526974964	5.757436778	6.400907902	5.834874117	6.313167609	5.756924354
7.470132999	7.163425105	7.257725354	7.145286723	7.247263585	7.60750934	7.40047528	7.674902961	7.223641217	8.188771354	7.434024976
7.513112535	7.242882366	7.008189638	7.110119167	7.271765855	7.500514993	7.581745522	7.408350685	7.235909082	7.094423676	7.19451287
8.101080777	7.982844695	7.731643757	7.74135294	7.79502329	7.497258706	7.808037772	7.781778008	7.802412471	7.254772892	7.704713122
5.991832974	5.592417908	5.275108815	5.152354602	5.013949583	5.004202527	5.080610501	5.259362606	5.022380096	5.050145476	5.23641272
5.552687563	5.155231492	5.196669762	5.409806025	5.368370848	5.600363616	5.023334857	5.267553962	5.339321114	6.011350704	5.246153724
5.795984036	5.580716305	5.513478166	5.629112889	5.599121838	5.664812383	5.915689103	6.262617583	5.496694919	7.442955315	5.295256434
6.427392999	6.261837923	6.415310365	6.19331136	6.155499257	6.276339922	6.079083433	6.707927159	6.370417217	6.742907383	6.405649885
6.417075794	6.393554434	6.743809083	6.091159259	6.311837796	6.783973673	6.275468439	6.79711062	6.501089818	6.504395547	6.638346991
6.848790219	6.401108846	6.876572087	6.287156532	6.371035635	6.973534016	6.526121033	7.211343173	6.873301967	6.67541412	6.842218984
6.983257153	6.332298028	6.596720815	6.214790307	6.311363467	7.127248119	7.200756004	6.927083252	7.031301063	6.976660334	7.106788121
6.884534766	5.833009367	6.510630369	5.726294108	5.793501698	6.358445169	6.88550407	7.469874578	6.853483689	6.583373163	6.566932284
7.378665354	5.970044015	6.481111948	6.095073589	6.253389105	6.4616864	6.543061959	6.689413854	6.467652562	6.650781016	6.706099259
5.314190261	5.179903975	5.373879501	4.900348772	5.140147757	5.589944234	4.989615999	6.248350539	5.065884069	5.341384813	5.328305165
5.122460967	5.189884912	5.291390353	4.900348772	5.140147757	5.419743289	4.989615999	5.930005946	5.119542588	5.166695049	5.328305165
6.560979539	5.920328474	6.933804831	6.45408032	6.748498691	7.152162703	6.539932711	6.233049195	7.136832439	5.491923517	6.304010268
5.500303593	5.401794395	5.61033671	5.479268775	5.309290822	5.78815856	5.338591716	5.644992895	5.405266748	5.922160218	5.522700882
5.338389067	5.631910913	5.501103058	5.175926088	5.305705387	5.540412447	5.441957811	5.275040827	5.227154447	6.107412368	5.435216169
5.338389067	5.631910913	5.501103058	5.175926088	5.305705387	5.540412447	5.441957811	5.275040827	5.227154447	6.107412368	5.435216169
7.262387456	7.192305777	9.194011238	9.054987309	10.49990529	5.260382816	5.860071175	6.029111672	8.322877169	6.608152691	6.751602225
8.106834324	6.938341791	7.404493025	7.407460283	7.733644137	9.319500641	8.41420488	7.230425807	6.971114193	7.846472726	8.198990134
5.914326541	5.889762636	5.745756315	6.074139255	5.956812309	6.06182531	6.264520628	6.09486283	6.09402656	5.950152778	5.85996571
5.926991011	6.19781124	5.776577779	5.927854219	5.917678324	6.134111114	6.237198738	5.802202807	5.855825589	6.134086795	5.980543853
6.055876351	6.49646397	6.580245427	6.261979654	6.300181217	6.416468106	6.480816109	6.400002174	6.349855638	7.350058454	6.430361579
4.900405321	5.199272064	5.169597308	5.299817643	5.228993871	5.167864431	5.738801548	5.28287424	5.083993391	5.309487752	5.180299553
4.967069639	5.547606174	5.223199232	5.46280305	5.384504966	5.153180496	5.888793705	5.730154527	5.36373686	5.485434094	5.298228632
5.714424275	5.157243597	5.209725349	5.035609234	5.032757842	6.962439868	5.885859973	5.545270993	5.564744809	5.458861186	5.825728323
5.482280165	5.027642429	5.011321917	5.156870572	5.255252411	5.235903324	5.103147558	5.365951582	5.149345829	5.532568171	5.30442513
5.284076393	4.824680997	4.851666042	4.831237192	4.596532079	4.987709361	4.995546186	5.027879334	4.834606406	5.1883776	4.896745399
4.824015203	5.005947616	5.196884408	4.763759272	4.916035621	5.501289544	5.237186743	4.9772802	4.995369735	5.220064437	5.001813756
4.787395985	4.814883739	4.830920607	4.815246226	4.94891597	4.623695508	4.761401989	5.284741739	4.986680318	5.085865875	4.722350883
5.902249502	6.100566566	5.845207547	5.522374754	5.412979982	5.751369374	5.784859812	5.968587721	5.833405073	5.583499243	5.783436915
5.196412198	5.547997263	5.379920826	5.444755856	5.311810805	5.113881783	5.619782451	5.133266087	5.051108994	5.747137555	5.557149085
5.34430928	5.519873791	5.085527755	5.126781302	5.033059844	5.352812725	5.639672148	5.651354173	5.439694302	5.851332196	5.746344729
5.726326257	5.466536881	5.411400792	4.974965243	4.952943009	5.483811223	5.622543372	5.975548148	5.479261292	5.891539374	5.407984365

			Embryo				S	X_{mean}	CV
24DAP_Endosperm	16DAP_Embryo	18DAP_Embryo	20DAP_Embryo	22DAP_Embryo	24DAP_Embryo	18DAP_Pericarp			
14.94703959	15.03198922	15.08184243	14.73671161	15.02423301	15.04119612	15.00942463	0.115394704	14.98177666	0.007702338
9.401823552	14.15612887	14.00205385	13.96791036	14.2600858	14.50015141	13.78329141	1.794835356	13.43877789	0.133556442
11.75265706	14.42498441	14.37591012	14.07704121	14.08988464	14.09174551	14.21648515	0.940478207	13.86124401	0.067849481
9.063590147	7.141998758	6.61305679	7.440721921	8.276597869	9.328756254	13.2560256	2.229562387	8.731535334	0.255345973
5.553393352	7.96758116	8.035620577	8.747323041	9.117634798	9.919393423	12.68498715	2.169545583	8.860847643	0.24484628
6.04973631	8.402530217	8.581082257	9.365684779	9.869644189	10.65917809	13.38275932	2.255446617	9.472945023	0.238093498
5.441297813	12.30193327	11.97403847	11.27875736	10.92414346	11.06122394	12.63121334	2.450035263	10.80180109	0.226817291
7.38083957	12.41159247	12.21197404	11.58576441	11.41844265	11.44478392	12.66006361	1.797848363	11.30192295	0.159074555
6.293953392	11.34602941	11.4717509	11.04681393	11.61703017	12.27074272	10.47547824	1.995187267	10.64597125	0.187412423
7.034584662	13.13368253	13.01098817	12.72180366	12.65230454	12.76370479	8.8623705	2.45804486	11.45420555	0.214597586
6.079507501	10.6953785	10.78174792	11.2964738	11.80799992	12.83183728	9.425192656	2.181278392	10.4168768	0.209398502
6.079507501	10.6953785	10.78174792	11.2964738	11.80799992	12.83183728	9.425192656	2.181278392	10.4168768	0.209398502
7.025953463	11.58447582	11.56429975	11.56868234	12.11731633	12.42952827	12.67298168	1.928587205	11.28046252	0.170967033
9.160123637	11.32924458	11.21370915	11.20383552	11.16651133	11.66772647	13.22115786	1.185875267	11.28032979	0.105127712
9.525704544	11.78220931	11.67713832	11.6709376	11.61883203	12.1386841	13.55430711	1.181278705	11.70968757	0.100880463
10.8837724	11.21856916	11.26856015	11.09313332	11.08958932	11.12685582	11.32022181	0.144731116	11.14295743	0.012988573
12.05440926	12.73534051	12.42118161	12.30599131	12.4361335	12.1403503	12.88576821	0.300355522	12.42559639	0.024172322
8.620921617	11.29737306	11.28285453	11.8846959	12.60748991	13.05088922	13.37706106	1.597129361	11.73161219	0.13613895
8.595925693	11.63062527	11.69768259	12.38193491	13.09376998	13.60968698	13.9961316	1.806879872	12.14367957	0.148791794
11.89068736	13.09897839	12.98057904	13.01259308	12.94852935	13.20834793	13.37506671	0.482010777	12.93068312	0.037276513
11.78381491	13.08468525	12.95054222	12.96626124	12.92440422	13.17617107	13.34615896	0.510259227	12.89029112	0.039584771
11.78381491	13.08468525	12.95054222	12.96626124	12.92440422	13.17617107	13.34615896	0.510259227	12.89029112	0.039584771
11.56011976	12.29896012	8.555209895	10.74004992	10.66270429	10.91808253	14.05003078	1.683919814	11.25502247	0.149614967
13.26376641	10.21215528	8.839935819	10.90162707	11.72830528	10.23743666	12.54637549	1.519213347	11.10422886	0.136813944
13.67170577	11.26044904	10.32070059	12.20727779	12.6423669	11.13020371	13.41137352	1.24542096	12.09201105	0.102995354
10.44152129	9.079706172	5.662080985	8.384113529	8.364120103	8.416924495	10.98236478	1.726336627	8.761547336	0.197035588
6.168667128	6.132868899	5.335564866	6.050392717	5.565244515	4.921690023	7.298260675	0.76241276	5.924669832	0.12868443
6.281820216	6.690728552	5.189331527	6.256817701	5.62930139	5.21707255	8.142394458	1.027693179	6.201066628	0.165728453
7.606991638	7.31279099	6.316128766	6.39422423	6.300855907	6.90374737	8.315096367	0.766513694	7.021405038	0.109168135
10.44058463	8.179194383	8.020824782	7.678156731	7.834394119	7.597638952	8.463311173	0.982872901	8.316300681	0.118186311
8.538186612	11.54045884	11.23310272	11.37728269	11.71826846	11.90860367	7.937183684	1.642847161	10.60758381	0.154874775
5.695704268	5.211531916	5.593303015	5.243717795	5.974198216	5.510073233	5.927378572	0.300571327	5.593701002	0.053733892
5.660867041	6.09371668	5.811478723	5.74098779	5.947171534	5.868328101	5.817031401	0.141059263	5.84851161	0.024118831
5.893952439	6.546490896	6.366300699	6.361918857	6.517153786	6.313792918	6.167790298	0.222874615	6.309628556	0.035322937
5.893952439	6.546490896	6.366300699	6.361918857	6.517153786	6.313792918	6.167790298	0.222874615	6.309628556	0.035322937
6.133191731	6.105348384	6.152435112	6.080662701	6.254875944	6.197796872	10.43990986	1.62094476	6.766317229	0.239560858
6.310616387	5.707181301	5.621327483	5.627967014	5.989881926	5.870966777	6.648050553	0.385914427	5.967998777	0.064663959
6.336771774	6.019272093	5.22171043	5.295656943	5.853993359	5.905461223	6.062783946	0.409628024	5.813664253	0.070459525
7.116457313	6.686772425	6.636613818	6.257971249	6.765865194	6.752845282	10.23811194	1.359737852	7.207805317	0.188647971
6.691416029	7.518601153	7.123480241	7.102818863	7.298209286	6.937618487	7.037111653	0.262146409	7.101322245	0.036915155
8.164936411	7.046745156	7.058444618	6.66992008	7.121730568	7.56067226	7.02225257	0.48560316	7.23495738	0.067119008
5.745351813	6.67672837	6.323142678	6.804374048	7.1838014	7.818686536	6.07469044	0.698889713	6.660967898	0.104923147
7.416173845	7.07559081	7.093664443	6.966569336	7.315228456	7.545385457	8.010909613	0.35788552	7.346217423	0.048716979
7.190019183	7.490249602	7.231445378	7.111184111	7.388643611	7.286377317	7.425482972	0.136841072	7.303343168	0.018736771
7.8955003	8.283698233	8.557005652	8.455441118	8.312106124	8.050859977	7.862757944	0.271086793	8.202481335	0.033049364
5.095074691	4.996412057	4.920181678	4.975908395	4.986448986	4.773549379	5.430961601	0.203568674	5.025505255	0.040507106
5.281907268	5.04327994	5.500670059	5.009079652	5.208290305	5.305550126	5.108710026	0.171849908	5.208212482	0.032995948
5.683116543	5.614775098	5.499604344	5.651731994	5.872858435	5.524795453	6.046828717	0.196170731	5.699101512	0.034421344
6.419687623	6.177480677	6.569837376	6.225919362	6.337324204	6.380440793	6.653573778	0.17198803	6.39489483	0.026894583
6.083651595	6.696573066	6.79261534	6.576825732	6.545405769	6.925159983	7.426696175	0.408910415	6.720989666	0.060840804
6.270975823	6.984370042	7.375547272	6.769599944	7.014740549	7.437243612	8.285426871	0.630421404	7.16255773	0.08801624
6.364044223	6.574064994	5.97271328	6.36180109	6.079917783	6.172891602	7.023032336	0.353267168	6.364066473	0.05550966
5.981232244	5.457542777	5.852957007	5.002380931	5.347353609	5.234716143	5.748804204	0.35565404	5.517855274	0.064455123
6.038650701	6.01016739	6.164571805	5.493528446	5.955745505	5.809433955	6.559069131	0.325547736	6.004452419	0.054217723
5.197794594	5.265556406	5.503175858	5.299251536	5.300949955	5.30635422	5.134100706	0.114924935	5.286740468	0.021738335
5.254645349	5.25657887	5.404174552	5.321425533	5.301709001	5.30635422	5.283485763	0.050752406	5.304053327	0.009568608
5.445751474	6.037945251	5.841021069	6.181617909	5.904800606	6.409270977	5.33382547	0.384069418	5.879176108	0.065327082
5.665814409	5.148493685	5.647705668	5.338112913	5.326939373	5.323439362	5.741172326	0.225382215	5.455953962	0.041309406
5.593302314	5.264376054	5.640022394	5.511534862	5.417813958	5.460210178	5.649799846	0.138169495	5.505294229	0.025097568
5.593302314	5.264376054	5.640022394	5.511534862	5.417813958	5.460210178	5.649799846	0.138169495	5.505294229	0.025097568
5.89970022	6.077556705	5.493864613	5.989704638	5.851179041	7.570281833	6.210135007	0.6625657	6.156060294	0.107628202
8.065297697	5.825395736	5.362863901	5.13352167	5.565198963	4.994131128	5.593821737	1.041957712	5.791461547	0.179912739
6.229863424	6.069656423	6.05600932	6.671862654	6.358518139	8.36751342	6.028756297	0.836974007	6.540311382	0.127971584
6.482467778	5.807648768	6.238543709	6.256388088	6.067484061	6.500145446	6.15825092	0.240147744	6.215846967	0.038634758
6.290137732	6.97288059	6.794940781	6.443355323	7.124753302	7.0695342	6.566392639	0.324918694	6.75171351	0.048123886
5.538291892	5.473019108	5.675862486	5.520834098	5.469197778	5.301311506	5.187170155	0.161182022	5.452241003	0.029562527
5.686614087	5.546508464	5.894096345	5.646051577	5.60289397	5.362548289	5.325936193	0.194877005	5.580664132	0.034920038
5.219397374	5.074365814	4.813357808	4.98794355	4.892195191	5.196880825	5.287212713	0.177849232	5.067336182	0.035097184
5.226139733	4.843225936	5.093193103	4.911464891	5.45865399	5.003875358	5.488279184	0.255276197	5.146404599	0.049602823
4.731905123	4.856176352	4.814057321	5.047672297	4.858063639	4.823626206	4.937772108	0.100650392	4.867039007	0.020680005
4.894903536	4.919694111	5.003307158	4.998214193	5.007421853	4.886485609	4.859899429	0.062787749	4.938560841	0.012713774
4.691898198	4.869510279	5.03120718	4.655736654	5.062644544	5.997411773	5.093372292	0.450158725	5.057397274	0.089009959
5.865606064	5.780784958	5.777822817	5.861992921	5.731377708	5.658663686	5.86070455	0.078326758	5.790993243	0.013525617
5.385296807	5.21931812	5.008141488	5.344709998	5.215722577	5.186039928	5.300370267	0.124731479	5.237085598	0.023816964
5.234990709	5.415332925	5.322615971	5.252797322	5.381316785	5.374220657	5.829599377	0.200279992	5.401553392	0.037078221
5.103321157	5.547711521	5.363018834	5.249662705	5.57851118	5.293346291	6.086546958	0.322216035	5.460302664	0.059010655
