# Supplementary material for: Non-specific lipid transfer proteins in maize
Source: BMC Plant Biol. 2014 Oct 28;14:281. doi: 10.1186/s12870-014-0281-8 (PMC4226865; doi:10.1186/s12870-014-0281-8)
Supplement: Additional file 15: Table S11. — The probe sets of ZmLTP genes on the maize 18 k GeneChip in this study. [file 12870_2014_281_MOESM15_ESM.pdf]

Table S11. The probe sets of ZmLTP genes on the maize 18k GeneChip in this study.

Gene ID	Name	Probe Set ID	Match
GRMZM2G126397	ZmLTP1.1	Zm.7127.1.A1_at	15/15
GRMZM2G010868	ZmLTP1.2	Zm.714.1.A1_at	15/15
GRMZM2G107839	ZmLTP1.3	Zm.4168.1.A1_at	15/15
GRMZM2G096234	ZmLTP1.4	Zm.3909.1.A1_at	15/15
GRMZM2G101958	ZmLTP1.6a	Zm.2085.1.S1_s_at	15/15
GRMZM2G101958	ZmLTP1.6b	Zm.19.2.S1_x_at	10/15
GRMZM5G898755	ZmLTP1.7	Zm.16488.1.A1_at	15/15
GRMZM2G137329	ZmLTP2.1	Zm.930.1.A1_at	15/15
GRMZM2G406552	ZmLTP2.3	Zm.11801.1.S1_at	15/15
GRMZM2G387360	ZmLTP2.4	Zm.17087.1.S1_at	15/15
GRMZM2G403007	ZmLTP2.6	Zm.13789.1.A1_at	9/15
GRMZM2G004909	ZmLTP2.7	Zm.8222.1.A1_at	8/15
GRMZM2G320373	ZmLTP2.8	Zm.17726.1.A1_at	8/15
GRMZM2G393150	ZmLTP2.9	Zm.9640.1.A1_at	15/15
GRMZM2G073377	ZmLTPc2	Zm.10322.1.S1_at	11/15
GRMZM2G031102	ZmLTPd1	Zm.3374.1.S1_at	10/15
GRMZM2G071771	ZmLTPd2	Zm.11586.1.A1_at	15/15
GRMZM2G136364	ZmLTPd3	Zm.16476.1.A1_at	15/15
GRMZM2G099867	ZmLTPd4	Zm.3374.1.S1_at	10/15
GRMZM2G164440	ZmLTPd5	Zm.1090.2.A1_a_at	15/15
GRMZM2G087413	ZmLTPd6	Zm.253.1.A1_at	14/15
GRMZM2G065557	ZmLTPd8	Zm.4529.1.A1_a_at	15/15
GRMZM2G094632	ZmLTPd9	Zm.3825.1.A1_at	15/15
GRMZM2G170969	ZmLTPd13a	Zm.4302.2.A1_x_at	15/15
GRMZM2G170969	ZmLTPd13b	Zm.4302.2.A1_a_at	15/15
GRMZM2G170969	ZmLTPd13c	Zm.4302.1.A1_x_at	11/15
GRMZM2G091054	ZmLTPd14	Zm.8180.1.S1_at	14/15
GRMZM5G850455	ZmLTPg1	Zm.5674.1.S1_at	15/15
GRMZM2G078876	ZmLTPg3	Zm.1503.2.S1_at	15/15
GRMZM2G083725	ZmLTPg4	ZmAffx.191.1.A1_at	15/15
GRMZM2G005991	ZmLTPg7a	Zm.3313.1.A1_a_at	11/15
GRMZM2G005991	ZmLTPg7b	Zm.3313.2.A1_x_at	9/15
GRMZM2G005991	ZmLTPg7c	Zm.3313.1.A1_x_at	7/15
GRMZM2G168833	ZmLTPg9	Zm.2246.1.A1_at	14/15
GRMZM2G130454	ZmLTPg16	Zm.3724.3.A1_a_at	15/15
GRMZM2G141858	ZmLTPg17	Zm.1477.1.S1_at	15/15
GRMZM2G116167	ZmLTPg18	Zm.1046.1.S1_at	15/15
GRMZM2G089400	ZmLTPg19	Zm.14674.1.S1_at	15/15
GRMZM2G089288	ZmLTPg20	Zm.2305.1.S1_at	15/15
GRMZM2G170044	ZmLTPg22	Zm.10158.1.A1_at	15/15
GRMZM2G171597	ZmLTPg23	Zm.16112.1.S1_at	13/15
