# Supplementary material for: Non-specific lipid transfer proteins in maize
Source: BMC Plant Biol. 2014 Oct 28;14:281. doi: 10.1186/s12870-014-0281-8 (PMC4226865; doi:10.1186/s12870-014-0281-8)
Supplement: Additional file 17: Table S12. — Primers used for real time PCR analysis. [file 12870_2014_281_MOESM17_ESM.pdf]

Table S12. Primers used for real time PCR analysis.

Gene name	Forward primer	Reverse primer
ZmLTP2.4	5’ GGCGATCATCAGCGGGTC 3’	5’ CGTTGGGGCTGTTGATGTAG 3’
ZmLTP2.6	5’ CCATCATCATCGGGAGGTCG 3’	5’ GAGTTGACGTAGCGCTGAAG 3’
ZmLTP2.8	5’ GTGTCGCGCTGTTCCTGG 3’	5’ GCTGGGGTTCCTCTTGTACT 3’
ZmLTPd2	5’ GTCTGCAACCTGTCGAGC 3’	5’ TGTAGAACCTCACCCACACG 3’
ZmLTPd8	5’ CTTCGCGGGGCTGGATAC 3’	5’ TTGGGGATGGTCATGGCC 3’
ZmLTPd9	5’ GGACTGCTGCAAAACCATCA 3’	5’ CAGTCCTGCTTTGATCCGC 3’
ZmLTPg16	5’ GTGCCTCAACTACATCAGCG 3’	5’ GCGTCCTGTCGATGGTGA 3’
ZmLTPg18	5’ CTGCATCTTCAGCTTCCACC 3’	5’ GCCCACCACCAGATGAAACT 3’
