# Supplementary material for: Non-specific lipid transfer proteins in maize
Source: BMC Plant Biol. 2014 Oct 28;14:281. doi: 10.1186/s12870-014-0281-8 (PMC4226865; doi:10.1186/s12870-014-0281-8)
Supplement: Additional file 18: Table S13. — List of FPKM values of 42 ZmLTP genes in maize reproductive (ovary) and vegetative tissue (leaf) under both drought and well-watered conditions. [file 12870_2014_281_MOESM18_ESM.pdf]

Table S13. List of FPKM values of 42 ZmLTP genes in maize reproductive (ovary) and vegetative tissue (leaf) under both drought and well-watered conditions.

MCC and MCD stand for maize ovary, well watered and drought, respectively; MLC and MLD stand for maize basal leaf meristem, well watered and drought, respectively.

Numbers 1 and 2 indicate the two biological replicates.

The extent of differential expression is measured in terms of fold change and (-) indicates failure to calculate or undetected values.

Values in red and blue indicate the fold increase and decrease in expression in the drought-stressed tissue, respectively.

Name	Gene ID	MCC.1	MCC.2	Average	MCD.1	MCD.2	Average	Ovaries (Fold Change)
ZmLTP2.8	GRMZM2G320373	10.426	8.22402	9.32501	293.913	176.666	235.2895	25.2320
ZmLTPg14	GRMZM2G071575	9.68297	16.8128	13.24789	61.5871	103.104	82.34555	6.2158
ZmLTP2.2	GRMZM2G081464	1.17237	0.748169	0.96027	3.5746	1.58045	2.577525	2.6842
ZmLTPd4	GRMZM2G099867	2.14948	0.981653	1.565567	4.88947	0.547447	2.718459	1.7364
ZmLTPd1	GRMZM2G031102	2.11308	0.96503	1.539055	4.80667	0.538177	2.672424	1.7364
ZmLTPc2	GRMZM2G073377	1.56115	1.01596	1.288555	0.980828	2.97719	1.979009	1.5358
ZmLTP2.6	GRMZM2G403007	20.1625	24.8437	22.5031	0.39234	0.198247	0.295294	76.2060
ZmLTP2.1	GRMZM2G137329	20.9869	53.3202	37.15355	1.43846	0.464549	0.951505	39.0470
ZmLTP1.2	GRMZM2G010868	595.094	1436.18	1015.637	43.0125	36.1625	39.5875	25.6550
ZmLTP1.1	GRMZM2G126397	1342.75	1562.15	1452.45	95.4805	59.8011	77.6408	18.7070
ZmLTPg9	GRMZM2G168833	3.19474	4.07053	3.632635	0.239117	0.202985	0.221051	16.4335
ZmLTPg6	GRMZM2G006047	5.36824	7.43594	6.40209	0.359445	0.443662	0.401554	15.9430
ZmLTPg24	GRMZM2G379035	48.1625	51.1694	49.66595	3.98622	3.2204	3.60331	13.7830
ZmLTPg21	GRMZM2G046750	2.39596	1.93677	2.166365	0.539786	0.122738	0.331262	6.5397
ZmLTPg1	GRMZM5G850455	184.23	207.755	195.9925	33.3676	26.5791	29.97335	6.5389
ZmLTPg3	GRMZM2G078876	176.717	208.607	192.662	29.2066	29.9628	29.5847	6.5122
ZmLTPg2	GRMZM2G174680	6.97953	10.3239	8.651715	2.00723	1.43356	1.720395	5.0289
ZmLTPg17	GRMZM2G141858	56.2329	63.9697	60.1013	12.3026	17.8129	15.05775	3.9914
ZmLTPd9	GRMZM2G094632	11.0245	10.6958	10.86015	2.95937	3.5894	3.274385	3.3167
ZmLTPg19	GRMZM2G089400	16.672	17.0799	16.87595	5.23999	5.11543	5.17771	3.2593
ZmLTPg16	GRMZM2G130454	3.44783	4.29984	3.873835	1.0493	1.33798	1.19364	3.2454
ZmLTPd3	GRMZM2G136364	14.2103	13.8827	14.0465	8.36008	0.513462	4.436771	3.1659
ZmLTPg5	GRMZM2G097137	1.00325	0.586983	0.795117	0.211924	0.428934	0.320429	2.4814
ZmLTPd12	GRMZM2G471051	0.702159	0.983819	0.842989	0.650837	0.287756	0.469297	1.7963
ZmLTPg10	GRMZM2G414620	0.847816	0.277165	0.562491	0.351432	0	0.351432	1.6006
ZmLTPg4	GRMZM2G083725	346.72	771.911	559.3155	357.561	454.026	405.7935	1.3783
ZmLTPd5	GRMZM2G164440	57.0507	54.5583	55.8045	32.5263	51.9309	42.2286	1.3215
ZmLTP2.7	GRMZM2G004909	5.39505	4.01455	4.7048	5.7845	1.54254	3.66352	1.2842
ZmLTPg20	GRMZM2G089288	5.96294	5.88883	5.925885	3.01775	6.73541	4.87658	1.2152
ZmLTPg22	GRMZM2G170044	8.03274	6.02072	7.02673	6.88194	5.5397	6.21082	1.1314
ZmLTP1.3	GRMZM2G107839	261.266	559.841	410.5535	0	0	0	-
ZmLTPd13	GRMZM2G170969	118.475	45.6443	82.05965	0	0	0	-
ZmLTPg18	GRMZM2G116167	151.749	170.997	161.373	0	0	0	-

Name	Gene ID	MLC.1	MLC.2	Average	MLD.1	MLD.2	Average	Leaf (Fold Change)
ZmLTPd3	GRMZM2G136364	98.5154	32.6996	65.6075	226.254	468.784	347.519	5.2969
ZmLTP2.8	GRMZM2G320373	23.0335	10.6743	16.8539	39.3189	86.4044	62.86165	3.7298
ZmLTPd4	GRMZM2G099867	10.7975	2.7424	6.76995	12.1264	35.7739	23.95015	3.5377
ZmLTPd1	GRMZM2G031102	10.6147	2.69596	6.65533	11.9211	35.1681	23.5446	3.5377
ZmLTP2.6	GRMZM2G403007	0.895399	0.06069	0.4780445	1.40634	1.94226	1.6743	3.5024
ZmLTPg2	GRMZM2G174680	1.6429	1.15136	1.39713	4.15883	5.15967	4.65925	3.3349
ZmLTP1.1	GRMZM2G126397	43.3601	5.88486	24.62248	42.5902	111.67	77.1301	3.1325
ZmLTPg3	GRMZM2G078876	30.2099	19.885	25.04745	64.7171	63.4785	64.0978	2.5591
ZmLTPg1	GRMZM5G850455	3.75822	3.0752	3.41671	7.42871	9.44568	8.437195	2.4694
ZmLTPd5	GRMZM2G164440	25.2971	39.8123	32.5547	58.0493	75.1908	66.62005	2.0464
ZmLTP1.5	GRMZM2G025026	1.27297	0.214966	0.743968	1.39798	1.15311	1.275545	1.7145
ZmLTP1.2	GRMZM2G010868	1156.78	330.585	743.6825	1042.53	1330.33	1186.43	1.5953
ZmLTP2.7	GRMZM2G004909	128.523	72.6659	100.59445	146.805	167.643	157.224	1.5629
ZmLTP1.3	GRMZM2G107839	2207.42	689.465	1448.4425	1911.63	2503.43	2207.53	1.5241
ZmLTPg19	GRMZM2G089400	11.3913	8.56868	9.97999	13.4208	14.3896	13.9052	1.3933
ZmLTPg22	GRMZM2G170044	67.4672	24.2184	45.8428	52.8522	73.1059	62.97905	1.3738
ZmLTPd8	GRMZM2G065557	9.94042	10.1019	10.02116	9.41453	14.1537	11.784115	1.1759
ZmLTPd10	AC233926.1_FG002	37.4067	26.9737	32.1902	41.5912	29.5362	35.5637	1.1048
ZmLTPg17	GRMZM2G141858	27.0536	18.9292	22.9914	23.715	26.1727	24.94385	1.0849
ZmLTPg25	GRMZM2G004466	22.973	22.0804	22.5267	23.6736	23.6156	23.6446	1.0496
ZmLTP2.9	GRMZM2G393150	0.593746	0.845037	0.7193915	0.381498	0.210728	0.296113	2.4294
ZmLTPg24	GRMZM2G379035	8.41691	0	8.41691	3.01347	5.1377	4.075585	2.0652
ZmLTPd11	GRMZM2G000221	34.5622	11.3914	22.9768	20.9225	4.41849	12.670495	1.8134
ZmLTPg10	GRMZM2G414620	0.451229	0.76671	0.6089695	0.371987	0.320803	0.346395	1.7580
ZmLTPg18	GRMZM2G116167	3.82673	0.101742	1.964236	0.837906	1.46976	1.153833	1.7024
ZmLTPg7	GRMZM2G005991	2.94005	2.43093	2.68549	2.28582	1.00237	1.644095	1.6334
ZmLTPg6	GRMZM2G006047	19.8552	16.8587	18.35695	17.5124	6.08085	11.796625	1.5561
ZmLTPg16	GRMZM2G130454	5.92326	3.69522	4.80924	5.12351	1.08849	3.106	1.5484
ZmLTPg21	GRMZM2G046750	8.18335	7.51481	7.84908	8.41245	1.96057	5.18651	1.5134
ZmLTPg13	GRMZM2G176347	6.45889	4.34133	5.40011	5.60318	1.89512	3.74915	1.4404
ZmLTPg5	GRMZM2G097137	3.19798	1.96057	2.579275	2.80344	0.788911	1.7961755	1.4360
ZmLTPg9	GRMZM2G168833	179.275	96.4799	137.87745	111.426	89.6491	100.53755	1.3714
ZmLTPg14	GRMZM2G071575	3.66903	2.20221	2.93562	2.55683	1.79477	2.1758	1.3492
ZmLTPg4	GRMZM2G083725	161.271	100.659	130.965	123.116	103.977	113.5465	1.1534
ZmLTPd9	GRMZM2G094632	6.11464	4.13128	5.12296	4.58564	4.76498	4.67531	1.0957
ZmLTPd2	GRMZM2G071771	82.2042	56.1774	69.1908	80.5053	54.7451	67.6252	1.0232
