# Supplementary material for: Non-specific lipid transfer proteins in maize
Source: BMC Plant Biol. 2014 Oct 28;14:281. doi: 10.1186/s12870-014-0281-8 (PMC4226865; doi:10.1186/s12870-014-0281-8)
Supplement: Additional file 20: Table S14. — The probe sets of ZmLTP genes on the maize 58 k NSF array in this study. [file 12870_2014_281_MOESM20_ESM.pdf]

Table S14. The probe sets of ZmLTP genes on the maize 58k NSF array in this study.

Gene ID	Name	Probe Set ID	Expect	Identities
GRMZM2G126397	ZmLTP1.1	MZ00057079	0	100.00%
GRMZM2G010868	ZmLTP1.2	MZ00056626	0	100.00%
GRMZM2G107839	ZmLTP1.3	MZ00041203	0	100.00%
GRMZM2G096234	ZmLTP1.4	MZ00001642	2.00E-99	91.90%
GRMZM2G025026	ZmLTP1.5	MZ00024911	0	100.00%
GRMZM2G101958	ZmLTP1.6	MZ00041611	1.00E-149	98.30%
GRMZM5G898755	ZmLTP1.7	MZ00040001	0	100.00%
GRMZM2G137329	ZmLTP2.1	MZ00026029	0	100.00%
GRMZM2G081464	ZmLTP2.2	MZ00021772	1.00E-135	97.10%
GRMZM2G387360	ZmLTP2.4	MZ00024305	0	100.00%
GRMZM2G039383	ZmLTP2.5	MZ00048959	2.00E-86	100.00%
GRMZM2G403007	ZmLTP2.6	MZ00028450	0	100.00%
GRMZM2G393150	ZmLTP2.9	MZ00019363	0	99.90%
AC225127.3_FG003	ZmLTPc1	MZ00023792	0	97.90%
GRMZM2G073377	ZmLTPc2	MZ00044799	0	96.30%
GRMZM2G071771	ZmLTPd2	MZ00031966	0	100.00%
GRMZM2G136364	ZmLTPd3a	MZ00056914	1.00E-167	100.00%
GRMZM2G136364	ZmLTPd3b	MZ00015921	1.00E-167	100.00%
GRMZM2G087413	ZmLTPd6	MZ00015063	1.00E-177	99.10%
GRMZM2G094632	ZmLTPd9	MZ00015293	0	97.90%
GRMZM2G471051	ZmLTPd12	MZ00022218	0	100.00%
GRMZM2G170969	ZmLTPd13	MZ00041900	0	100.00%
GRMZM5G850455	ZmLTPg1	MZ00023652	0	99.40%
GRMZM2G006047	ZmLTPg6	MZ00057049	0	100.00%
GRMZM2G168833	ZmLTPg9	MZ00029700	0	93.30%
GRMZM2G414620	ZmLTPg10	MZ00021034	0	99.20%
GRMZM2G151021	ZmLTPg11	MZ00051921	0	100.00%
GRMZM2G071575	ZmLTPg14	MZ00046275	0	100.00%
GRMZM2G130454	ZmLTPg16	MZ00030532	1.00E-179	100.00%
GRMZM2G141858	ZmLTPg17a	MZ00025985	0	99.90%
GRMZM2G141858	ZmLTPg17b	MZ00043905	0	100.00%
GRMZM2G089400	ZmLTPg19	MZ00018623	0	98.10%
GRMZM2G089288	ZmLTPg20	MZ00015331	0	98.90%
GRMZM2G170044	ZmLTPg22	MZ00019645	0	99.80%
GRMZM2G171597	ZmLTPg23	MZ00044499	0	99.00%
GRMZM2G379035	ZmLTPg24	MZ00057310	0	100.00%
GRMZM2G036063	ZmLTPx1.3	MZ00051527	0	100.00%
