# Supplementary material for: Non-specific lipid transfer proteins in maize
Source: BMC Plant Biol. 2014 Oct 28;14:281. doi: 10.1186/s12870-014-0281-8 (PMC4226865; doi:10.1186/s12870-014-0281-8)
Supplement: Additional file 21: Figure S7. — Microarray-based expression profiles of 36 probe sets representing 34 ZmLTP genes under cold stress condition in two inbred maize lines, chilling-sensitive ETH-DL3 and chilling-tolerant ETH-DH7. [file 12870_2014_281_MOESM21_ESM.pdf]

Table S7. Syntenic sites harboring nsLTP genes in maize, sorghum and rice.

Block ID	Gene	Chr	Start	End	Gene	Chr	Start	End
33	ZmLTPg6	1	287110656	287111891	ZmLTPg16	5	4429841	4431021
93	ZmLTPg11	2	47422297	47424522	ZmLTPx2	10	118762389	118764275
261	ZmLTPg13	2	233632142	233633151	ZmLTPg21	7	9201072	9202357
269	ZmLTPd7	3	162372093	162372586	ZmLTPd13	8	162756043	162756672
246	ZmLTP2.7	6	162646836	162647576	ZmLTP2.9	8	152130321	152131030
36	OsLTP1.1	1	6541005	6543076	ZmLTP1.4	8	25946151	25947246
43	OsLTPd9	1	33906370	33909592	ZmLTPd14	8	171565757	171566487
43	OsLTPg1	1	34626184	34629997	ZmLTPg24	8	171016014	171017268
11	OsLTP1.2	1	35129858	35130917	ZmLTP1.1	3	184434264	184435351
11	OsLTPd5	1	36483566	36484818	ZmLTPd8	3	179414637	179416401
44	OsLTPd1	1	39836639	39837257	ZmLTPd12	8	162752046	162752849
180	OsLTPg5	3	15300737	15301156	ZmLTPg23	7	167020090	167021470
134	OsLTPg5	3	15300737	15301156	ZmLTPg3	1	66023613	66026784
165	OsLTPg5	3	15300737	15301156	ZmLTPg12	2	212487102	212487524
135	OsLTPg10	3	33563702	33566773	ZmLTPg9	1	289786005	289788499
179	OsLTPg10	3	33563702	33566773	ZmLTPg22	7	16096163	16099019
207	OsLTPd6	4	20534150	20535514	ZmLTPd3	2	55687675	55688855
228	OsLTP1.4	5	23508004	23509669	ZmLTP1.1	3	184434264	184435351
327	OsLTP1.6	6	20254443	20257626	ZmLTP1.5	9	61863515	61865024
313	OsLTPg13	6	28617447	28620321	ZmLTPg19	6	89065818	89068077
292	OsLTPg13	6	28617447	28620321	ZmLTPg17	5	49545870	49548495
291	OsLTPg24	6	30113940	30115510	ZmLTPg18	5	59978801	59980128
348	OsLTPg17	7	3976186	3977717	ZmLTPg13	2	233632142	233633151
362	OsLTPg17	7	3976186	3977717	ZmLTPg21	7	9201072	9202357
334	OsLTPg19	7	5313788	5318164	ZmLTPg9	1	289786005	289788499
359	OsLTPg19	7	5313788	5318164	ZmLTPg22	7	16096163	16099019
361	OsLTPd3	7	11090520	11091267	ZmLTPd10	7	30954067	30954378
357	OsLTPg21	7	25920677	25922871	ZmLTPg23	7	167020090	167021470
343	OsLTPg21	7	25920677	25922871	ZmLTPg12	2	212487102	212487524
372	OsLTPg22	8	26554150	26557081	ZmLTPg4	1	194657095	194659202
396	OsLTPg22	8	26554150	26557081	ZmLTPg14	4	45262758	45263990
447	OsLTPc2	9	20534165	20534954	ZmLTPc2	7	141644107	141645776
46	SbLTPx1	1	4463665	4464105	ZmLTPg16	5	4429841	4431021
40	SbLTPg4	1	4460355	4461248	ZmLTPg13	2	233632142	233633151
47	SbLTP2.1	1	18272382	18273147	ZmLTP2.4	5	23838144	23838818
57	SbLTPd1	1	43752841	43754087	ZmLTPd9	5	39411646	39412902
58	SbLTPg5	1	57231144	57233750	ZmLTPg23	7	167020090	167021470
43	SbLTPg5	1	57231144	57233750	ZmLTPg12	2	212487102	212487524
63	SbLTPg7	1	67453781	67454898	ZmLTPg25	9	149245864	149247159
8	SbLTPg6	1	60370302	60371426	ZmLTPg2	1	52625970	52627201
172	SbLTPg9	2	4685954	4687002	ZmLTPg20	7	8901782	8904176
172	SbLTPg10	2	4753198	4754293	ZmLTPg21	7	9201072	9202357
153	SbLTPg10	2	4753198	4754293	ZmLTPg13	2	233632142	233633151
180	SbLTPg12	2	7100123	7103454	ZmLTPg22	7	16096163	16099019
176	SbLTPd5	2	13351451	13352128	ZmLTPd10	7	30954067	30954378
178	SbLTPc1	2	65488459	65488989	ZmLTPc2	7	141644107	141645776
171	SbLTPg13	2	74005754	74008032	ZmLTPg23	7	167020090	167021470
146	SbLTPg13	2	210788762	212649098	ZmLTPg12	2	212487102	212487524
237	SbLTP1.1	3	1446324	1447200	ZmLTP1.4	8	25946151	25947246
209	SbLTP1.2	3	66219316	66220110	ZmLTP1.1	3	184434264	184435351

209	SbLTPd9	3	67522375	67523472	ZmLTPd8	3	179414637	179416401
235	SbLTPd7	3	65227168	65227630	ZmLTPd14	8	171565757	171566487
235	SbLTPg15	3	65758095	65759880	ZmLTPg24	8	171016014	171017268
234	SbLTPd10	3	70899337	70899654	ZmLTPd12	8	162752046	162752849
271	SbLTPg16	4	53770386	53770992	ZmLTPg15	4	126473476	126474249
391	SbLTPd11	6	44576681	44577477	ZmLTPd3	2	55687675	55688855
365	SbLTPg18	6	46903482	46905662	ZmLTPx2	10	118762389	118764275
383	SbLTPg18	6	46903482	46905662	ZmLTPg11	2	47422297	47424522
383	SbLTPg19	6	48688841	48689610	ZmLTPg10	2	41484533	41485576
421	SbLTP1.3	7	2833270	2834356	ZmLTP1.8	10	84608056	84608542
412	SbLTPg21	7	61309583	61311559	ZmLTPg4	1	194657095	194659202
438	SbLTPg21	7	61309583	61311559	ZmLTPg14	4	45262758	45263990
471	SbLTP1.4	8	2779571	2780575	ZmLTP1.6	10	4165442	4166593
486	SbLTP1.4	8	2779571	2780575	ZmLTP1.2	3	230606444	230608235
549	SbLTPg22	9	53507894	53509662	ZmLTPg24	8	171016014	171017268
564	SbLTP2.5	9	56662520	56663221	ZmLTP2.9	8	152130321	152131030
125	SbLTP1.9	10	46718450	46719021	ZmLTP1.5	9	61863515	61865024
107	SbLTPg23	10	57671753	57674547	ZmLTPg19	6	89065818	89068077
81	SbLTPg23	10	57671753	57674547	ZmLTPg17	5	49545870	49548495
105	SbLTP2.7	10	59161545	59162227	ZmLTP2.5	6	86281901	86282589
79	SbLTPg24	10	59476463	59477591	ZmLTPg18	5	59978801	59980128
