# Supplementary material for: Non-specific lipid transfer proteins in maize
Source: BMC Plant Biol. 2014 Oct 28;14:281. doi: 10.1186/s12870-014-0281-8 (PMC4226865; doi:10.1186/s12870-014-0281-8)
Supplement: Additional file 8: Figure S2. — Unrooted phylogenetic tree between maize, sorghum, rice and Arabidopsis nsLTP gene families. Different species are denoted with different colors (maize in black, sorghum in green, rice in blue and Arabidopsis in red). [file 12870_2014_281_MOESM8_ESM.pdf]

Table S8. Genes list corresponding to the GO enrichment analysis by the AgriGO program.

GO_acc	term_type	Term	query item	query total	bgitem	bgtotal	pvalue	FDR
GO:0006869	P	lipid transport	47	53	104	39203	2.40E-110	8.00E-109
GO:0010876	P	lipid localization	47	53	104	39203	2.40E-110	8.00E-109
GO:0033036	P	macromolecule localization	47	53	997	39203	6.50E-68	1.50E-66
GO:0051234	P	establishment of localization	47	53	3841	39203	6.20E-41	8.40E-40
GO:0006810	P	transport	47	53	3841	39203	6.20E-41	8.40E-40
GO:0051179	P	localization	47	53	3887	39203	1.10E-40	1.20E-39
GO:0048871	P	multicellular organismal homeostasis	12	53	1758	39203	3.20E-06	1.90E-05
GO:0042309	P	homoiothermy	12	53	1758	39203	3.20E-06	1.90E-05
GO:0050826	P	response to freezing	12	53	1758	39203	3.20E-06	1.90E-05
GO:0009409	P	response to cold	12	53	1758	39203	3.20E-06	1.90E-05
GO:0001659	P	temperature homeostasis	12	53	1758	39203	3.20E-06	1.90E-05
GO:0009266	P	response to temperature stimulus	12	53	1765	39203	3.40E-06	1.90E-05
GO:0009628	P	response to abiotic stimulus	12	53	1856	39203	5.60E-06	2.90E-05
GO:0032501	P	multicellular organismal process	12	53	1983	39203	1.10E-05	5.30E-05
GO:0042592	P	homeostatic process	12	53	2047	39203	1.50E-05	6.80E-05
GO:0065008	P	regulation of biological quality	12	53	2139	39203	2.30E-05	9.80E-05
GO:0006950	P	response to stress	12	53	3059	39203	0.00066	0.0026
GO:0050896	P	response to stimulus	12	53	3421	39203	0.0018	0.0066
GO:0065007	P	biological regulation	12	53	6272	39203	0.13	0.46
GO:0008289	F	lipid binding	47	53	202	39203	1.10E-98	9.40E-98
GO:0005488	F	binding	52	53	24279	39203	3.20E-10	1.30E-09
GO:0050825	F	ice binding	12	53	1758	39203	3.20E-06	6.80E-06
GO:0050824	F	water binding	12	53	1758	39203	3.20E-06	6.80E-06

entries
// ZmLTPg4 // ZmLTP1.7.1 // ZmLTP1.7.2 // ZmLTPd3.1 // ZmLTPd3.2 // ZmLTPg19 // ZmLTPg20.2 // ZmLTPd1 // ZmLTPd2 // ZmLTPg9 // ZmLTPg21 // ZmLTPg24 // ZmLTPg3 // ZmLTPg7 // ZmLTP1.4 // ZmLTPg12 // ZmLTPg22 // ZmLTPg10 // ZmLTPg17.1 // ZmLTPg17.2 // ZmLTPg5 // ZmLTPd10 // ZmLTPg2 // ZmLTPg16 // ZmLTPg8.1 // ZmLTP1.3.1 // ZmLTP1.3.2 // ZmLTPg8.2 // ZmLTPg20.1 // ZmLTPg14 // ZmLTPg25 // ZmLTPg18 // ZmLTPd4 // ZmLTPd8 // ZmLTP1.2.1 // ZmLTP1.2.2 // ZmLTPg6 // ZmLTPd11 // ZmLTPg23 // ZmLTP1.8 // ZmLTP1.6 // ZmLTP1.1 // ZmLTPg1.1 // ZmLTPg1.2 // ZmLTPg1.3 // ZmLTP1.5.2 // ZmLTP1.5.1
// ZmLTPg4 // ZmLTP1.7.1 // ZmLTP1.7.2 // ZmLTPd3.1 // ZmLTPd3.2 // ZmLTPg19 // ZmLTPg20.2 // ZmLTPd1 // ZmLTPd2 // ZmLTPg9 // ZmLTPg21 // ZmLTPg24 // ZmLTPg3 // ZmLTPg7 // ZmLTP1.4 // ZmLTPg12 // ZmLTPg22 // ZmLTPg10 // ZmLTPg17.1 // ZmLTPg17.2 // ZmLTPg5 // ZmLTPd10 // ZmLTPg2 // ZmLTPg16 // ZmLTPg8.1 // ZmLTP1.3.1 // ZmLTP1.3.2 // ZmLTPg8.2 // ZmLTPg20.1 // ZmLTPg14 // ZmLTPg25 // ZmLTPg18 // ZmLTPd4 // ZmLTPd8 // ZmLTP1.2.1 // ZmLTP1.2.2 // ZmLTPg6 // ZmLTPd11 // ZmLTPg23 // ZmLTP1.8 // ZmLTP1.6 // ZmLTP1.1 // ZmLTPg1.1 // ZmLTPg1.2 // ZmLTPg1.3 // ZmLTP1.5.2 // ZmLTP1.5.1
// ZmLTPg4 // ZmLTP1.7.1 // ZmLTP1.7.2 // ZmLTPd3.1 // ZmLTPd3.2 // ZmLTPg19 // ZmLTPg20.2 // ZmLTPd1 // ZmLTPd2 // ZmLTPg9 // ZmLTPg21 // ZmLTPg24 // ZmLTPg3 // ZmLTPg7 // ZmLTP1.4 // ZmLTPg12 // ZmLTPg22 // ZmLTPg10 // ZmLTPg17.1 // ZmLTPg17.2 // ZmLTPg5 // ZmLTPd10 // ZmLTPg2 // ZmLTPg16 // ZmLTPg8.1 // ZmLTP1.3.1 // ZmLTP1.3.2 // ZmLTPg8.2 // ZmLTPg20.1 // ZmLTPg14 // ZmLTPg25 // ZmLTPg18 // ZmLTPd4 // ZmLTPd8 // ZmLTP1.2.1 // ZmLTP1.2.2 // ZmLTPg6 // ZmLTPd11 // ZmLTPg23 // ZmLTP1.8 // ZmLTP1.6 // ZmLTP1.1 // ZmLTPg1.1 // ZmLTPg1.2 // ZmLTPg1.3 // ZmLTP1.5.2 // ZmLTP1.5.1
// ZmLTPg4 // ZmLTP1.7.1 // ZmLTP1.7.2 // ZmLTPd3.1 // ZmLTPd3.2 // ZmLTPg19 // ZmLTPg20.2 // ZmLTPd1 // ZmLTPd2 // ZmLTPg9 // ZmLTPg21 // ZmLTPg24 // ZmLTPg3 // ZmLTPg7 // ZmLTP1.4 // ZmLTPg12 // ZmLTPg22 // ZmLTPg10 // ZmLTPg17.1 // ZmLTPg17.2 // ZmLTPg5 // ZmLTPd10 // ZmLTPg2 // ZmLTPg16 // ZmLTPg8.1 // ZmLTP1.3.1 // ZmLTP1.3.2 // ZmLTPg8.2 // ZmLTPg20.1 // ZmLTPg14 // ZmLTPg25 // ZmLTPg18 // ZmLTPd4 // ZmLTPd8 // ZmLTP1.2.1 // ZmLTP1.2.2 // ZmLTPg6 // ZmLTPd11 // ZmLTPg23 // ZmLTP1.8 // ZmLTP1.6 // ZmLTP1.1 // ZmLTPg1.1 // ZmLTPg1.2 // ZmLTPg1.3 // ZmLTP1.5.2 // ZmLTP1.5.1
// ZmLTPg4 // ZmLTP1.7.1 // ZmLTP1.7.2 // ZmLTPd3.1 // ZmLTPd3.2 // ZmLTPg19 // ZmLTPg20.2 // ZmLTPd1 // ZmLTPd2 // ZmLTPg9 // ZmLTPg21 // ZmLTPg24 // ZmLTPg3 // ZmLTPg7 // ZmLTP1.4 // ZmLTPg12 // ZmLTPg22 // ZmLTPg10 // ZmLTPg17.1 // ZmLTPg17.2 // ZmLTPg5 // ZmLTPd10 // ZmLTPg2 // ZmLTPg16 // ZmLTPg8.1 // ZmLTP1.3.1 // ZmLTP1.3.2 // ZmLTPg8.2 // ZmLTPg20.1 // ZmLTPg14 // ZmLTPg25 // ZmLTPg18 // ZmLTPd4 // ZmLTPd8 // ZmLTP1.2.1 // ZmLTP1.2.2 // ZmLTPg6 // ZmLTPd11 // ZmLTPg23 // ZmLTP1.8 // ZmLTP1.6 // ZmLTP1.1 // ZmLTPg1.1 // ZmLTPg1.2 // ZmLTPg1.3 // ZmLTP1.5.2 // ZmLTP1.5.1
// ZmLTPg4 // ZmLTP1.7.1 // ZmLTP1.7.2 // ZmLTPd3.1 // ZmLTPd3.2 // ZmLTPg19 // ZmLTPg20.2 // ZmLTPd1 // ZmLTPd2 // ZmLTPg9 // ZmLTPg21 // ZmLTPg24 // ZmLTPg3 // ZmLTPg7 // ZmLTP1.4 // ZmLTPg12 // ZmLTPg22 // ZmLTPg10 // ZmLTPg17.1 // ZmLTPg17.2 // ZmLTPg5 // ZmLTPd10 // ZmLTPg2 // ZmLTPg16 // ZmLTPg8.1 // ZmLTP1.3.1 // ZmLTP1.3.2 // ZmLTPg8.2 // ZmLTPg20.1 // ZmLTPg14 // ZmLTPg25 // ZmLTPg18 // ZmLTPd4 // ZmLTPd8 // ZmLTP1.2.1 // ZmLTP1.2.2 // ZmLTPg6 // ZmLTPd11 // ZmLTPg23 // ZmLTP1.8 // ZmLTP1.6 // ZmLTP1.1 // ZmLTPg1.1 // ZmLTPg1.2 // ZmLTPg1.3 // ZmLTP1.5.2 // ZmLTP1.5.1
// ZmLTP2.8 // ZmLTP2.1 // ZmLTPg1.2 // ZmLTP1.4 // ZmLTP2.4 // ZmLTPg22 // ZmLTPg16 // ZmLTPg18 // ZmLTPd5 // ZmLTPg6 // ZmLTPg13 // ZmLTPg3
// ZmLTP2.8 // ZmLTP2.1 // ZmLTPg1.2 // ZmLTP1.4 // ZmLTP2.4 // ZmLTPg22 // ZmLTPg16 // ZmLTPg18 // ZmLTPd5 // ZmLTPg6 // ZmLTPg13 // ZmLTPg3
// ZmLTP2.8 // ZmLTP2.1 // ZmLTPg1.2 // ZmLTP1.4 // ZmLTP2.4 // ZmLTPg22 // ZmLTPg16 // ZmLTPg18 // ZmLTPd5 // ZmLTPg6 // ZmLTPg13 // ZmLTPg3
// ZmLTP2.8 // ZmLTP2.1 // ZmLTPg1.2 // ZmLTP1.4 // ZmLTP2.4 // ZmLTPg22 // ZmLTPg16 // ZmLTPg18 // ZmLTPd5 // ZmLTPg6 // ZmLTPg13 // ZmLTPg3
// ZmLTP2.8 // ZmLTP2.1 // ZmLTPg1.2 // ZmLTP1.4 // ZmLTP2.4 // ZmLTPg22 // ZmLTPg16 // ZmLTPg18 // ZmLTPd5 // ZmLTPg6 // ZmLTPg13 // ZmLTPg3
// ZmLTP2.8 // ZmLTP2.1 // ZmLTPg1.2 // ZmLTP1.4 // ZmLTP2.4 // ZmLTPg22 // ZmLTPg16 // ZmLTPg18 // ZmLTPd5 // ZmLTPg6 // ZmLTPg13 // ZmLTPg3
// ZmLTP2.8 // ZmLTP2.1 // ZmLTPg1.2 // ZmLTP1.4 // ZmLTP2.4 // ZmLTPg22 // ZmLTPg16 // ZmLTPg18 // ZmLTPd5 // ZmLTPg6 // ZmLTPg13 // ZmLTPg3
// ZmLTP2.8 // ZmLTP2.1 // ZmLTPg1.2 // ZmLTP1.4 // ZmLTP2.4 // ZmLTPg22 // ZmLTPg16 // ZmLTPg18 // ZmLTPd5 // ZmLTPg6 // ZmLTPg13 // ZmLTPg3
// ZmLTP2.8 // ZmLTP2.1 // ZmLTPg1.2 // ZmLTP1.4 // ZmLTP2.4 // ZmLTPg22 // ZmLTPg16 // ZmLTPg18 // ZmLTPd5 // ZmLTPg6 // ZmLTPg13 // ZmLTPg3
// ZmLTP2.8 // ZmLTP2.1 // ZmLTPg1.2 // ZmLTP1.4 // ZmLTP2.4 // ZmLTPg22 // ZmLTPg16 // ZmLTPg18 // ZmLTPd5 // ZmLTPg6 // ZmLTPg13 // ZmLTPg3
// ZmLTP2.8 // ZmLTP2.1 // ZmLTPg1.2 // ZmLTP1.4 // ZmLTP2.4 // ZmLTPg22 // ZmLTPg16 // ZmLTPg18 // ZmLTPd5 // ZmLTPg6 // ZmLTPg13 // ZmLTPg3
// ZmLTP2.8 // ZmLTP2.1 // ZmLTPg1.2 // ZmLTP1.4 // ZmLTP2.4 // ZmLTPg22 // ZmLTPg16 // ZmLTPg18 // ZmLTPd5 // ZmLTPg6 // ZmLTPg13 // ZmLTPg3
// ZmLTP2.8 // ZmLTP2.1 // ZmLTPg1.2 // ZmLTP1.4 // ZmLTP2.4 // ZmLTPg22 // ZmLTPg16 // ZmLTPg18 // ZmLTPd5 // ZmLTPg6 // ZmLTPg13 // ZmLTPg3
// ZmLTPg4 // ZmLTP1.7.1 // ZmLTP1.7.2 // ZmLTPd3.1 // ZmLTPd3.2 // ZmLTPg19 // ZmLTPg20.2 // ZmLTPd1 // ZmLTPd2 // ZmLTPg9 // ZmLTPg21 // ZmLTPg24 // ZmLTPg3 // ZmLTPg7 // ZmLTP1.4 // ZmLTPg12 // ZmLTPg22 // ZmLTPg10 // ZmLTPg17.1 // ZmLTPg17.2 // ZmLTPg5 // ZmLTPd10 // ZmLTPg2 // ZmLTPg16 // ZmLTPg8.1 // ZmLTP1.3.1 // ZmLTP1.3.2 // ZmLTPg8.2 // ZmLTPg20.1 // ZmLTPg14 // ZmLTPg25 // ZmLTPg18 // ZmLTPd4 // ZmLTPd8 // ZmLTP1.2.1 // ZmLTP1.2.2 // ZmLTPg6 // ZmLTPd11 // ZmLTPg23 // ZmLTP1.8 // ZmLTP1.6 // ZmLTP1.1 // ZmLTPg1.1 // ZmLTPg1.2 // ZmLTPg1.3 // ZmLTP1.5.2 // ZmLTP1.5.1
// ZmLTPg4 // ZmLTP1.7.1 // ZmLTP1.7.2 // ZmLTPd3.1 // ZmLTPd3.2 // ZmLTPg19 // ZmLTPg20.2 // ZmLTPd1 // ZmLTPd2 // ZmLTPg9 // ZmLTPg21 // ZmLTPg24 // ZmLTPg13 // ZmLTPg3 // ZmLTP2.8 // ZmLTP2.1 // ZmLTPg7 // ZmLTP1.4 // ZmLTPg12 // ZmLTPg22 // ZmLTPg10 // ZmLTPg17.1 // ZmLTPg17.2 // ZmLTPg5 // ZmLTPd10 // ZmLTPg2 // ZmLTPg16 // ZmLTPg8.1 // ZmLTPg1.2 // ZmLTP1.3.2 // ZmLTPg8.2 // ZmLTPg20.1 // ZmLTP2.4 // ZmLTPg25 // ZmLTPg18 // ZmLTPd4 // ZmLTPd8 // ZmLTP1.2.1 // ZmLTP1.2.2 // ZmLTPg6 // ZmLTPg14 // ZmLTPd11 // ZmLTPg23 // ZmLTP1.8 // ZmLTP1.6 // ZmLTP1.3.1 // ZmLTP1.1 // ZmLTPg1.1 // ZmLTPd5 // ZmLTPg1.3 // ZmLTP1.5.2 // ZmLTP1.5.1
// ZmLTP2.8 // ZmLTP2.1 // ZmLTPg1.2 // ZmLTP1.4 // ZmLTP2.4 // ZmLTPg22 // ZmLTPg16 // ZmLTPg18 // ZmLTPd5 // ZmLTPg6 // ZmLTPg13 // ZmLTPg3
// ZmLTP2.8 // ZmLTP2.1 // ZmLTPg1.2 // ZmLTP1.4 // ZmLTP2.4 // ZmLTPg22 // ZmLTPg16 // ZmLTPg18 // ZmLTPd5 // ZmLTPg6 // ZmLTPg13 // ZmLTPg3
