# Supplementary material for: Non-specific lipid transfer proteins in maize
Source: BMC Plant Biol. 2014 Oct 28;14:281. doi: 10.1186/s12870-014-0281-8 (PMC4226865; doi:10.1186/s12870-014-0281-8)
Supplement: Additional file 12: Table S8. — Genes list corresponding to the GO enrichment analysis by the AgriGO program. [file 12870_2014_281_MOESM12_ESM.pdf]

Table S8. Genes list corresponding to the GO enrichment analysis by the AgriGO program.

GO_acc	term_type	Term	query item	query total	bgitem	bgtotal	pvalue	FDR
GO:0006869	P	lipid transport	47	53	104	39203	########	########
GO:0010876	P	lipid localization	47	53	104	39203	########	########
GO:0033036	P	macromolecule localization	47	53	997	39203	6.50E-68	1.50E-66
GO:0051234	P	establishment of localization	47	53	3841	39203	6.20E-41	8.40E-40
GO:0006810	P	transport	47	53	3841	39203	6.20E-41	8.40E-40
GO:0051179	P	localization	47	53	3887	39203	1.10E-40	1.20E-39
GO:0048871	P	multicellular organismal homeostasis	12	53	1758	39203	3.20E-06	1.90E-05
GO:0042309	P	homoiothermy	12	53	1758	39203	3.20E-06	1.90E-05
GO:0050826	P	response to freezing	12	53	1758	39203	3.20E-06	1.90E-05
GO:0009409	P	response to cold	12	53	1758	39203	3.20E-06	1.90E-05
GO:0001659	P	temperature homeostasis	12	53	1758	39203	3.20E-06	1.90E-05
GO:0009266	P	response to temperature stimulus	12	53	1765	39203	3.40E-06	1.90E-05
GO:0009628	P	response to abiotic stimulus	12	53	1856	39203	5.60E-06	2.90E-05
GO:0032501	P	multicellular organismal process	12	53	1983	39203	1.10E-05	5.30E-05
GO:0042592	P	homeostatic process	12	53	2047	39203	1.50E-05	6.80E-05
GO:0065008	P	regulation of biological quality	12	53	2139	39203	2.30E-05	9.80E-05
GO:0006950	P	response to stress	12	53	3059	39203	0.00066	0.0026
GO:0050896	P	response to stimulus	12	53	3421	39203	0.0018	0.0066
GO:0065007	P	biological regulation	12	53	6272	39203	0.13	0.46
GO:0008289	F	lipid binding	47	53	202	39203	1.10E-98	9.40E-98
GO:0005488	F	binding	52	53	24279	39203	3.20E-10	1.30E-09
GO:0050825	F	ice binding	12	53	1758	39203	3.20E-06	6.80E-06
GO:0050824	F	water binding	12	53	1758	39203	3.20E-06	6.80E-06

entries
// ZmLTPg4 // ZmLTP1.7.1 // ZmLTP1.7.2 // ZmLTPd3.1 // ZmLTPd3.2 // ZmLTPg19 // ZmLTPg20.2 // ZmLTPd1 // ZmLTPd2 // ZmLTPg9 // ZmLTPg21 // ZmLTPg24 // ZmLTPg3 // ZmLTPg7 // ZmLTP1.4 // ZmLTPg12 // ZmLTPg22 // ZmLTPg10 // ZmLTPg17.1 // ZmLTPg17.2 // ZmLTPg5 // ZmLTPd10 // ZmLTPg2 // ZmLTPg16 // ZmLTPg8.1 // ZmLTP1.3.1 // ZmLTP1.3.2 // ZmLTPg8.2 // ZmLTPg20.1 // ZmLTPg14 // ZmLTPg25 // ZmLTPg18 // ZmLTPd4 // ZmLTPd8 // ZmLTP1.2.1 // ZmLTP1.2.2 // ZmLTPg6 // ZmLTPd11 // ZmLTPg23 // ZmLTP1.8 // ZmLTP1.6 // ZmLTP1.1 // ZmLTPg1.1 // ZmLTPg1.2 // ZmLTPg1.3 // ZmLTP1.5.2 // ZmLTP1.5.1
// ZmLTPg4 // ZmLTP1.7.1 // ZmLTP1.7.2 // ZmLTPd3.1 // ZmLTPd3.2 // ZmLTPg19 // ZmLTPg20.2 // ZmLTPd1 // ZmLTPd2 // ZmLTPg9 // ZmLTPg21 // ZmLTPg24 // ZmLTPg3 // ZmLTPg7 // ZmLTP1.4 // ZmLTPg12 // ZmLTPg22 // ZmLTPg10 // ZmLTPg17.1 // ZmLTPg17.2 // ZmLTPg5 // ZmLTPd10 // ZmLTPg2 // ZmLTPg16 // ZmLTPg8.1 // ZmLTP1.3.1 // ZmLTP1.3.2 // ZmLTPg8.2 // ZmLTPg20.1 // ZmLTPg14 // ZmLTPg25 // ZmLTPg18 // ZmLTPd4 // ZmLTPd8 // ZmLTP1.2.1 // ZmLTP1.2.2 // ZmLTPg6 // ZmLTPd11 // ZmLTPg23 // ZmLTP1.8 // ZmLTP1.6 // ZmLTP1.1 // ZmLTPg1.1 // ZmLTPg1.2 // ZmLTPg1.3 // ZmLTP1.5.2 // ZmLTP1.5.1
// ZmLTPg4 // ZmLTP1.7.1 // ZmLTP1.7.2 // ZmLTPd3.1 // ZmLTPd3.2 // ZmLTPg19 // ZmLTPg20.2 // ZmLTPd1 // ZmLTPd2 // ZmLTPg9 // ZmLTPg21 // ZmLTPg24 // ZmLTPg3 // ZmLTPg7 // ZmLTP1.4 // ZmLTPg12 // ZmLTPg22 // ZmLTPg10 // ZmLTPg17.1 // ZmLTPg17.2 // ZmLTPg5 // ZmLTPd10 // ZmLTPg2 // ZmLTPg16 // ZmLTPg8.1 // ZmLTP1.3.1 // ZmLTP1.3.2 // ZmLTPg8.2 // ZmLTPg20.1 // ZmLTPg14 // ZmLTPg25 // ZmLTPg18 // ZmLTPd4 // ZmLTPd8 // ZmLTP1.2.1 // ZmLTP1.2.2 // ZmLTPg6 // ZmLTPd11 // ZmLTPg23 // ZmLTP1.8 // ZmLTP1.6 // ZmLTP1.1 // ZmLTPg1.1 // ZmLTPg1.2 // ZmLTPg1.3 // ZmLTP1.5.2 // ZmLTP1.5.1
// ZmLTPg4 // ZmLTP1.7.1 // ZmLTP1.7.2 // ZmLTPd3.1 // ZmLTPd3.2 // ZmLTPg19 // ZmLTPg20.2 // ZmLTPd1 // ZmLTPd2 // ZmLTPg9 // ZmLTPg21 // ZmLTPg24 // ZmLTPg3 // ZmLTPg7 // ZmLTP1.4 // ZmLTPg12 // ZmLTPg22 // ZmLTPg10 // ZmLTPg17.1 // ZmLTPg17.2 // ZmLTPg5 // ZmLTPd10 // ZmLTPg2 // ZmLTPg16 // ZmLTPg8.1 // ZmLTP1.3.1 // ZmLTP1.3.2 // ZmLTPg8.2 // ZmLTPg20.1 // ZmLTPg14 // ZmLTPg25 // ZmLTPg18 // ZmLTPd4 // ZmLTPd8 // ZmLTP1.2.1 // ZmLTP1.2.2 // ZmLTPg6 // ZmLTPd11 // ZmLTPg23 // ZmLTP1.8 // ZmLTP1.6 // ZmLTP1.1 // ZmLTPg1.1 // ZmLTPg1.2 // ZmLTPg1.3 // ZmLTP1.5.2 // ZmLTP1.5.1
// ZmLTPg4 // ZmLTP1.7.1 // ZmLTP1.7.2 // ZmLTPd3.1 // ZmLTPd3.2 // ZmLTPg19 // ZmLTPg20.2 // ZmLTPd1 // ZmLTPd2 // ZmLTPg9 // ZmLTPg21 // ZmLTPg24 // ZmLTPg3 // ZmLTPg7 // ZmLTP1.4 // ZmLTPg12 // ZmLTPg22 // ZmLTPg10 // ZmLTPg17.1 // ZmLTPg17.2 // ZmLTPg5 // ZmLTPd10 // ZmLTPg2 // ZmLTPg16 // ZmLTPg8.1 // ZmLTP1.3.1 // ZmLTP1.3.2 // ZmLTPg8.2 // ZmLTPg20.1 // ZmLTPg14 // ZmLTPg25 // ZmLTPg18 // ZmLTPd4 // ZmLTPd8 // ZmLTP1.2.1 // ZmLTP1.2.2 // ZmLTPg6 // ZmLTPd11 // ZmLTPg23 // ZmLTP1.8 // ZmLTP1.6 // ZmLTP1.1 // ZmLTPg1.1 // ZmLTPg1.2 // ZmLTPg1.3 // ZmLTP1.5.2 // ZmLTP1.5.1
// ZmLTPg4 // ZmLTP1.7.1 // ZmLTP1.7.2 // ZmLTPd3.1 // ZmLTPd3.2 // ZmLTPg19 // ZmLTPg20.2 // ZmLTPd1 // ZmLTPd2 // ZmLTPg9 // ZmLTPg21 // ZmLTPg24 // ZmLTPg3 // ZmLTPg7 // ZmLTP1.4 // ZmLTPg12 // ZmLTPg22 // ZmLTPg10 // ZmLTPg17.1 // ZmLTPg17.2 // ZmLTPg5 // ZmLTPd10 // ZmLTPg2 // ZmLTPg16 // ZmLTPg8.1 // ZmLTP1.3.1 // ZmLTP1.3.2 // ZmLTPg8.2 // ZmLTPg20.1 // ZmLTPg14 // ZmLTPg25 // ZmLTPg18 // ZmLTPd4 // ZmLTPd8 // ZmLTP1.2.1 // ZmLTP1.2.2 // ZmLTPg6 // ZmLTPd11 // ZmLTPg23 // ZmLTP1.8 // ZmLTP1.6 // ZmLTP1.1 // ZmLTPg1.1 // ZmLTPg1.2 // ZmLTPg1.3 // ZmLTP1.5.2 // ZmLTP1.5.1
// ZmLTP2.8 // ZmLTP2.1 // ZmLTPg1.2 // ZmLTP1.4 // ZmLTP2.4 // ZmLTPg22 // ZmLTPg16 // ZmLTPg18 // ZmLTPd5 // ZmLTPg6 // ZmLTPg13 // ZmLTPg3
// ZmLTP2.8 // ZmLTP2.1 // ZmLTPg1.2 // ZmLTP1.4 // ZmLTP2.4 // ZmLTPg22 // ZmLTPg16 // ZmLTPg18 // ZmLTPd5 // ZmLTPg6 // ZmLTPg13 // ZmLTPg3
// ZmLTP2.8 // ZmLTP2.1 // ZmLTPg1.2 // ZmLTP1.4 // ZmLTP2.4 // ZmLTPg22 // ZmLTPg16 // ZmLTPg18 // ZmLTPd5 // ZmLTPg6 // ZmLTPg13 // ZmLTPg3
// ZmLTP2.8 // ZmLTP2.1 // ZmLTPg1.2 // ZmLTP1.4 // ZmLTP2.4 // ZmLTPg22 // ZmLTPg16 // ZmLTPg18 // ZmLTPd5 // ZmLTPg6 // ZmLTPg13 // ZmLTPg3
// ZmLTP2.8 // ZmLTP2.1 // ZmLTPg1.2 // ZmLTP1.4 // ZmLTP2.4 // ZmLTPg22 // ZmLTPg16 // ZmLTPg18 // ZmLTPd5 // ZmLTPg6 // ZmLTPg13 // ZmLTPg3
// ZmLTP2.8 // ZmLTP2.1 // ZmLTPg1.2 // ZmLTP1.4 // ZmLTP2.4 // ZmLTPg22 // ZmLTPg16 // ZmLTPg18 // ZmLTPd5 // ZmLTPg6 // ZmLTPg13 // ZmLTPg3
// ZmLTP2.8 // ZmLTP2.1 // ZmLTPg1.2 // ZmLTP1.4 // ZmLTP2.4 // ZmLTPg22 // ZmLTPg16 // ZmLTPg18 // ZmLTPd5 // ZmLTPg6 // ZmLTPg13 // ZmLTPg3
// ZmLTP2.8 // ZmLTP2.1 // ZmLTPg1.2 // ZmLTP1.4 // ZmLTP2.4 // ZmLTPg22 // ZmLTPg16 // ZmLTPg18 // ZmLTPd5 // ZmLTPg6 // ZmLTPg13 // ZmLTPg3
// ZmLTP2.8 // ZmLTP2.1 // ZmLTPg1.2 // ZmLTP1.4 // ZmLTP2.4 // ZmLTPg22 // ZmLTPg16 // ZmLTPg18 // ZmLTPd5 // ZmLTPg6 // ZmLTPg13 // ZmLTPg3
// ZmLTP2.8 // ZmLTP2.1 // ZmLTPg1.2 // ZmLTP1.4 // ZmLTP2.4 // ZmLTPg22 // ZmLTPg16 // ZmLTPg18 // ZmLTPd5 // ZmLTPg6 // ZmLTPg13 // ZmLTPg3
// ZmLTP2.8 // ZmLTP2.1 // ZmLTPg1.2 // ZmLTP1.4 // ZmLTP2.4 // ZmLTPg22 // ZmLTPg16 // ZmLTPg18 // ZmLTPd5 // ZmLTPg6 // ZmLTPg13 // ZmLTPg3
// ZmLTP2.8 // ZmLTP2.1 // ZmLTPg1.2 // ZmLTP1.4 // ZmLTP2.4 // ZmLTPg22 // ZmLTPg16 // ZmLTPg18 // ZmLTPd5 // ZmLTPg6 // ZmLTPg13 // ZmLTPg3
// ZmLTP2.8 // ZmLTP2.1 // ZmLTPg1.2 // ZmLTP1.4 // ZmLTP2.4 // ZmLTPg22 // ZmLTPg16 // ZmLTPg18 // ZmLTPd5 // ZmLTPg6 // ZmLTPg13 // ZmLTPg3
// ZmLTPg4 // ZmLTP1.7.1 // ZmLTP1.7.2 // ZmLTPd3.1 // ZmLTPd3.2 // ZmLTPg19 // ZmLTPg20.2 // ZmLTPd1 // ZmLTPd2 // ZmLTPg9 // ZmLTPg21 // ZmLTPg24 // ZmLTPg3 // ZmLTPg7 // ZmLTP1.4 // ZmLTPg12 // ZmLTPg22 // ZmLTPg10 // ZmLTPg17.1 // ZmLTPg17.2 // ZmLTPg5 // ZmLTPd10 // ZmLTPg2 // ZmLTPg16 // ZmLTPg8.1 // ZmLTP1.3.1 // ZmLTP1.3.2 // ZmLTPg8.2 // ZmLTPg20.1 // ZmLTPg14 // ZmLTPg25 // ZmLTPg18 // ZmLTPd4 // ZmLTPd8 // ZmLTP1.2.1 // ZmLTP1.2.2 // ZmLTPg6 // ZmLTPd11 // ZmLTPg23 // ZmLTP1.8 // ZmLTP1.6 // ZmLTP1.1 // ZmLTPg1.1 // ZmLTPg1.2 // ZmLTPg1.3 // ZmLTP1.5.2 // ZmLTP1.5.1
// ZmLTPg4 // ZmLTP1.7.1 // ZmLTP1.7.2 // ZmLTPd3.1 // ZmLTPd3.2 // ZmLTPg19 // ZmLTPg20.2 // ZmLTPd1 // ZmLTPd2 // ZmLTPg9 // ZmLTPg21 // ZmLTPg24 // ZmLTPg13 // ZmLTPg3 // ZmLTP2.8 // ZmLTP2.1 // ZmLTPg7 // ZmLTP1.4 // ZmLTPg12 // ZmLTPg22 // ZmLTPg10 // ZmLTPg17.1 // ZmLTPg17.2 // ZmLTPg5 // ZmLTPd10 // ZmLTPg2 // ZmLTPg16 // ZmLTPg8.1 // ZmLTPg1.2 // ZmLTP1.3.2 // ZmLTPg8.2 // ZmLTPg20.1 // ZmLTP2.4 // ZmLTPg25 // ZmLTPg18 // ZmLTPd4 // ZmLTPd8 // ZmLTP1.2.1 // ZmLTP1.2.2 // ZmLTPg6 // ZmLTPg14 // ZmLTPd11 // ZmLTPg23 // ZmLTP1.8 // ZmLTP1.6 // ZmLTP1.3.1 // ZmLTP1.1 // ZmLTPg1.1 // ZmLTPd5 // ZmLTPg1.3 // ZmLTP1.5.2 // ZmLTP1.5.1
// ZmLTP2.8 // ZmLTP2.1 // ZmLTPg1.2 // ZmLTP1.4 // ZmLTP2.4 // ZmLTPg22 // ZmLTPg16 // ZmLTPg18 // ZmLTPd5 // ZmLTPg6 // ZmLTPg13 // ZmLTPg3
// ZmLTP2.8 // ZmLTP2.1 // ZmLTPg1.2 // ZmLTP1.4 // ZmLTP2.4 // ZmLTPg22 // ZmLTPg16 // ZmLTPg18 // ZmLTPd5 // ZmLTPg6 // ZmLTPg13 // ZmLTPg3
